# NMR Hydrophilic Metabolomic Analysis of Bacterial Resistance Pathways Using Multivalent Antimicrobials with Challenged and Unchallenged Wild Type and Mutated Gram-Positive Bacteria

**DOI:** 10.3390/ijms222413606

**Published:** 2021-12-19

**Authors:** Michelle L. Aries, Mary J. Cloninger

**Affiliations:** Department of Chemistry and Biochemistry, Montana State University, Bozeman, MT 59717, USA; ariesm.data@outlook.com

**Keywords:** metabolomics, quaternary ammonium compounds, nuclear magnetic resonance, dendrimers, DABCO, antibiotic resistance, Gram-positive bacteria, *Bacillus cereus*, membrane disruption

## Abstract

Multivalent membrane disruptors are a relatively new antimicrobial scaffold that are difficult for bacteria to develop resistance to and can act on both Gram-positive and Gram-negative bacteria. Proton Nuclear Magnetic Resonance (^1^H NMR) metabolomics is an important method for studying resistance development in bacteria, since this is both a quantitative and qualitative method to study and identify phenotypes by changes in metabolic pathways. In this project, the metabolic differences between wild type *Bacillus cereus* (*B. cereus)* samples and *B. cereus* that was mutated through 33 growth cycles in a nonlethal dose of a multivalent antimicrobial agent were identified. For additional comparison, samples for analysis of the wild type and mutated strains of *B. cereus* were prepared in both challenged and unchallenged conditions. A C_16_-DABCO (1,4-diazabicyclo-2,2,2-octane) and mannose functionalized poly(amidoamine) dendrimer (DABCOMD) were used as the multivalent quaternary ammonium antimicrobial for this hydrophilic metabolic analysis. Overall, the study reported here indicates that *B. cereus* likely change their peptidoglycan layer to protect themselves from the highly positively charged DABCOMD. This membrane fortification most likely leads to the slow growth curve of the mutated, and especially the challenged mutant samples. The association of these sample types with metabolites associated with energy expenditure is attributed to the increased energy required for the membrane fortifications to occur as well as to the decreased diffusion of nutrients across the mutated membrane.

## 1. Introduction

The number of bacteria that develop resistance to known antibiotics is rapidly increasing. Antibiotic resistant bacteria are a universal health concern, especially since the majority of bacteria in hospitals are already resistant to at least one class of antimicrobial [1,2,3,4,5]. Moreover, the number of new antibiotics being developed every year decreases due to the challenges of effectively dispatching both antibiotic resistant bacteria and novel infectious bacteria. Both Gram-negative and Gram-positive bacteria have become drug resistant [3]. Therefore, synthesizing an antimicrobial agent that works on both Gram negative and Gram-positive infectious bacteria is generally preferred.

*Bacillus cereus* (*B. cereus*) is a Gram-positive bacterium that infects humans, and is an appropriate bacterium on which to conduct antimicrobial studies [6,7]. Humans encounter *B. cereus* throughout the environment, and this strain of bacteria has been shown to produce a variety of compounds toxic to humans [5,7,8,9]. *B. cereus* can lead to a variety of infectious outcomes ranging from food poisoning to fatal meningitis, with food poisoning comprising the majority of infections [5,6,8,9]. It also has a short generation time, is easily mutable, has high mutation rates, and has a wide body of prior research reports [6,7,8,9]. Mutations in *B. cereus* that result in antibiotic resistance occur in the usual ways: randomly occurring genetic mutations, horizontal gene transfer, fortification of the cell wall (alterations in composition of the peptidoglycan layer), changes to the efflux pumps, and antibiotic degrading or altering enzymes [5,10,11,12,13,14]). Bacteria are proficient at DNA uptake from the environment, viral phage DNA injection, and bacterial plasmid swapping resulting in horizontal gene transfer. Antibiotic resistant bacterial communities can protect even distantly related antibiotic susceptible bacteria from antibiotics [15].

Fortification of bacterial cell walls is a common mutation which results in changes to the composition of the peptidoglycan layer since a significant quantity of antibiotics must pass through the cell wall for the antibiotic to be effective [12,16,17,18,19]. The pentapeptide side chains linked to N-acetylglucosamine and N-acetylmuramic acid in the peptidoglycan are responsible for crosslinking [16,17,19,20,21]. Changing the composition of the pentapeptide side chains is a relatively easy way for bacteria to attempt to repel environmental stressors, such as being grown in the presence of an antimicrobial agent, since these changes alter the cell wall’s permeability, charge ratios, and patterns of noncovalent interactions [16,17,19]. Products from aminoacyl-tRNA biosynthesis can be diverted away from protein synthesis and toward peptidoglycan synthesis when alterations to the pentapeptide side chain would offer additional protection against antimicrobials [17]. Glycine, for example, is involved in the crosslinking of the pentapeptide side chains of the peptidoglycan [17,22], and a decrease in crosslinking has been shown to decrease overall bacterial survivability [23]. In addition, when alanine is converted to lactate in the pentapeptide side chain that truncates the D-ala-D-ala tail, resistance to vancomycin occurs [19]. Lysine is a positively charged amino acid that is incorporated into the pentapeptide side chain to create a greater overall positive charge, which can aid in repelling positively charged antimicrobials [17]. Thus, changing hydrophobicity, charge, permeability, rigidity and crosslinking by switching components of pentapeptide side chains have been shown to increase bacterial survival [10,17,19,23]. These mutations have been shown to increase bacterial resistance to antimicrobial agents, thus decreasing the availability of effective antibiotics to treat infections [4,11,19].

It is of the upmost importance to develop innovative antibiotics based upon new scaffolds such that resistant and novel bacteria are less likely to have automatic resistance, or develop resistance upon exposure, in comparison to conventional antibiotics. One way to increase the potency of an antibiotic is by using a multivalent framework such as a dendrimer, which can serve as a multivalent delivery agent by presenting a concentrated dose of the antibiotic. Dendrimers can interact with multiple cellular receptors simultaneously, since multiple antibiotic units are bound to each dendrimer [1,24,25]. The multivalent presentation can lower the total requisite concentration of antibiotic since dendrimers have increased local concentrations of the antimicrobial endgroups relative to the monomeric antimicrobials [26,27,28]. Dendrimers are attractive scaffolds to use for multivalent antimicrobials because a plethora of antibiotic types can be readily attached to the scaffold in a variety of densities, and the size of the dendrimer can be systematically altered by varying the dendrimer generation [1,24,25,29,30]. The poly(amidoamine) (PAMAM) dendrimers used in this report can be functionalized with multiple units of amino acids, antibiotics, carbohydrates, and other bioactive assemblages [28,31,32,33].

Carbohydrate functionalized PAMAM dendrimers were previously shown to act as antimicrobial agents [34,35]. Further functionalization of glycodendrimers with antibiotic moieties were shown to increase the effectiveness of the antibiotics relative to their monomeric counterparts by increasing activity and/or making it more difficult for the bacteria to develop resistance. In addition, the carbohydrates can increase the system’s solubility [1,30,36,37].

Quite a few of the conventual small molecule inhibitors target one pathway and require specific substrate binding affinity. Therefore, if the bacteria can alter that specific binding interaction, they develop (or start to develop) resistance [38,39]. An advantage of some membrane disruptors is that the antibiotic does not have to be taken into the cell where it could be degraded, pumped out, or sequestered [39]. Causing membrane disruption while presenting a multivalent barrage makes it unlikely that the bacteria can quickly and effectively develop resistance, since they would have to change their membrane to such a degree it would entail numerous mutations [39,40,41]. An added befit of membrane disruptors is that they also cause energy and nutrient disruption since they damage the membrane and induce membrane rigidity [39].

**Figure 1:**
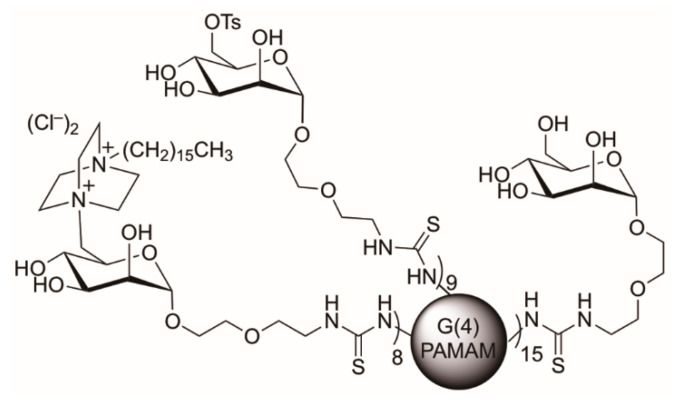
C_16_-DABCO Mannose Functionalized Dendrimer (DABCOMD) Structure.

Quaternary ammonium compounds (QACs) are common membrane disruptors. QACs have a positively charged ammonium group that is attracted to the bacterial phospholipid membrane, enabling membrane disruption and/or lysis [1,42,43,44]. QACs are used as common surfactants and disinfectants [1,42,43,44,45]. The QAC of interest in this study is 1,4-diazabicyclo-2,2,2-octane (DABCO) [46] with an attached 16-carbon alkyl chain (C_16_-DABCO). DABCO kills both Gram positive and Gram-negative bacteria [43]. A multivalent display of C_16_-DABCO was appended to the mannosides on a mannose functionalized PAMAM dendrimer (DABCOMD) to create a multivalent antimicrobial (Figure 1) [27]. DABCOMD presents multiple positively charged units and was used against both Gram positive and Gram-negative bacteria to ascertain the minimum inhibitory concentration (MIC) by VanKoten et al. in his 2016 study. After the MIC study was completed for 33 growth cycles, DABCOMD was 100-fold more potent against *B. cereus* than its monomeric C_16_-DABCO counterpart. After the MIC study, *B. cereus* became very resistant to monomeric antimicrobials such as ampicillin and monomeric DABCO, but MIC values for multivalent DABCOMD remained relatively unchanged [27]. The *B. cereus* sample arising after 33 growth cycles was collected and labeled as mutant (Mut) for metabolomic resistance pathway analysis. The mutant was compared to the wild type (WT, commercially available bacterial stock that was used to start the MIC study) using nuclear magnetic resonance (NMR) hydrophilic metabolomics to better understand why *B. cereus* was unable to develop protective resistance against DABCOMD over the 33 growth cycles of the MIC study. A DABCOMD-challenged (denoted Mut D or WT D) sample set of all sample types was also conducted by including a small amount of DABCOMD in the growth media while the samples were grown for sample collection. The unchallenged sample sets did not have anything added to the growth media (see the Materials and Methods section for additional details). With the challenged and unchallenged sample comparisons, we could compare how the mutant and wild type samples respond to being grown in an annoying but unlethal amount of DABCOMD. A summary of the sample sets that were used for this study, and the abbreviations that are used throughout the manuscript, are provided in Table 1.

Metabolomics, or the study of the small molecule biochemical byproducts of cellular metabolism (metabolites), is both a quantitative and qualitative method that can be used to compare two or more sample types by analyzing differences in their metabolite composition and concentration [47,48,49,50,51,52]. Metabolomic profiling enables the study of the interrelationship between an organism and its environment by measuring the mutations that occur, as well as the changes to the organism’s metabolic pathways that are incurred, upon mutation [3,47,48,51,52,53]. Metabolomics studies have been used in applications including determination of phenotypes, distinguishing biomarkers for diseases, and studying biofilms and other environmental factors affecting organisms, because they change rapidly with response environmental changes [3,47,50,53,54]. Untargeted metabolomics can give a more complete picture of the mutated pathways involved in an altered metabolism than a targeted approach, since unexpected changes frequently occur in nature [54]. NMR is a quantitative and reliable technique used to study differences between a wild type and mutated metabolism for over 40 years because it is a nondestructive method that does not require separation of metabolites and is capable of observing certain molecules (sugars, amines, and nonreactive species) more readily than mass spectrometry [3,48,49,50,54,55]. Here, we report an untargeted proton (^1^H) NMR hydrophilic metabolomics study that was used to observe metabolomic pathway alterations that occurred upon exposure of *B. cereus* to the multivalent antimicrobial agent DABCOMD. The goal of this study was to obtain increased comprehension of important mutations and major resistance strategies of Gram-positive bacteria upon exposure to multivalent cationic antimicrobial compounds.

## 2. Results

^1^H NMR spectra were obtained for the eight sample types listed in Table 1. Chenomx NMR Suite 8.4 software was used to profile each ^1^H NMR spectrum to obtain the identity and concentration of the metabolites found in each sample. MetaboAnalyst 5.0 was used for 2D and 3D sPLS–DA, ortho PLS–DA, and PCA to eliminate outliers in each sample type. Outliers were apparent as they did not cluster with the rest of their group. The unchallenged wild type mid log and stationary phases had five nonoutlier samples; the DABCOMD challenged wild type mid log phase had six nonoutlier samples; the DABCOMD challenged wild type stationary phase had five nonoutlier samples; the unchallenged mutant mid log phase had seven nonoutliers; the unchallenged mutant stationary phase had five nonoutlier samples; the DABCOMD challenged mutant mid log phase had six nonoutlier samples, and the DABCOMD challenged mutant stationary phase had seven nonoutlier samples.

**Figure 2:**
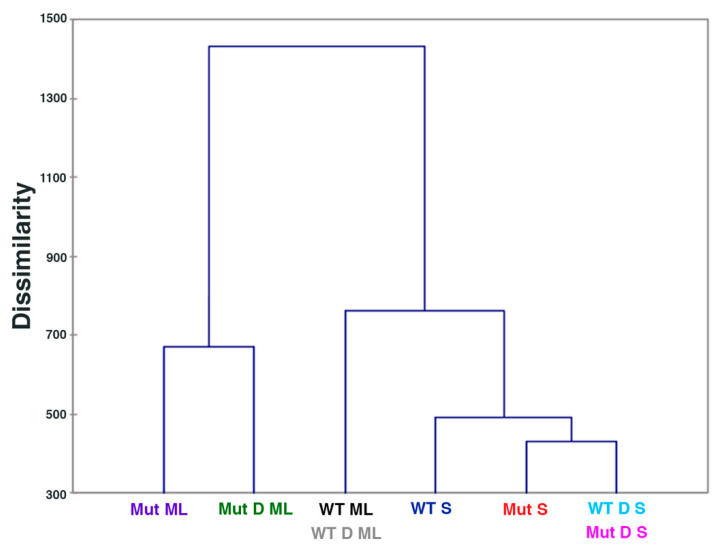
Hierarachical Clustering showing that WT ML and WT D ML cluster together, WT D S and Mut D S cluster together, and all other sample types are separate. Full names of sample groups are given in Table 1.

The metabolite identities and concentrations obtained from Chenomx NMR Suite 8.4 Software were input into XLSTAT Hierarchical Clustering with all sample types or with the following smaller groups of sample sets: mid log phase, stationary phase, challenged samples, and unchallenged samples. XLSTAT Hierarchical Clustering of all the sample types showed that WT ML and WT D ML were clustered together, Mut D S and WT D S were clustered together, and all other sample types were separated (Figure 2). Clustering of WT ML and WT D ML, and clustering of Mut D S and WT D S, were also observed in smaller groupings as shown in Figure S4 of the Supplementary Materials. Hierarchical Clustering of the unchallenged samples showed complete separation of all sample types (Figure S4 of the Supplementary Materials).

**Figure 3:**
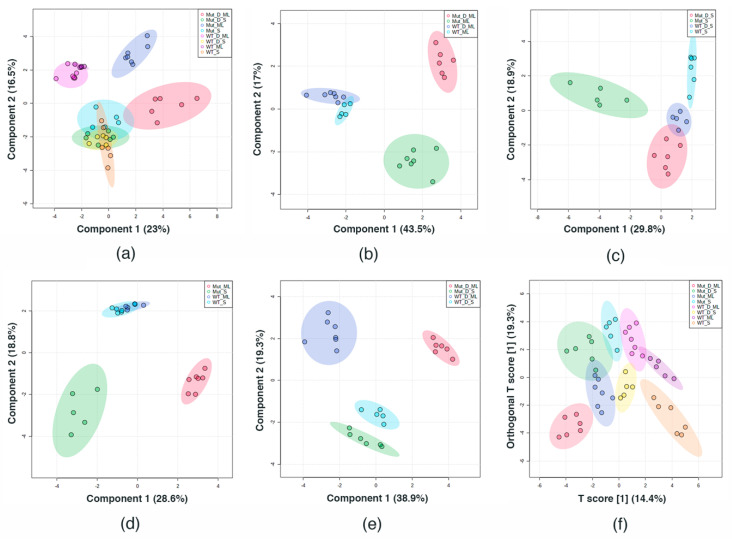
2D sPLS–DA (**a**) Contains all sample types: showing overlap of WT ML with WT D ML and the overlap of all stationary phases. Mut ML is separate, and Mut D ML is separate. (**b**) Contains only mid log samples, showing slight overlap of WT ML with WT D ML and complete separation of Mut ML and Mut D ML. (**c**) Contains only stationary phase samples, showing slight overlap of WT D S and Mut D S, and complete separation of WT S and Mut S. (**d**) Contains unchallenged samples, showing a complete separation of Mut ML and Mut S samples and a 2D overlap of the WT ML with WT S (overlap not present in the 3D plot (Figure S7d)). (**e**) Contains only DABCOMD challenged samples, showing complete separation of all sample types. (**f**) 2D ortho PLS–DA **c**ontaining all sample types: demonstrates the greatest degree of separation with all sample types together, with only an overlap of the oval of WT ML with the oval of WT D ML, and an overlap of the oval of Mut D S with the ovals of WT S and Mut ML.

XLSTAT and MetaboAnalyst 5.0 multivariate statistical analyses, PCA biplots, 2D and 3D sPLS–DA, and ortho PLS–DA were used to determine the statistical significance for each metabolite, the metabolite to sample correlation, and sample clustering. Figure 3 shows the 2D sPLS–DA for all the samples together (a), only the mid log phase samples (b), only the stationary phase samples (c), only unchallenged samples (d), only the DABCOMD challenged samples (e), and ortho PLS–DA for all the samples together (f). Three-dimensional sPLS–DA plots are provided in the Supplementary Materials. Figure 3a shows the overlap of samples when all samples are plotted together. The observed overlap is likely due to the greater difference between mid log phase samples; because the stationary phase samples are more similar, they cluster together in plots that include the mid log phase. Therefore, the data was also observed using the smaller groups shown in Figure 3b–e containing the comparison sets listed above to render the differences between sample types more readily observable.

When only the mid log phase samples were plotted, the wild type samples (WT ML and WT D ML) had a small overlap while the mutant samples had a large separation (Figure 3b). This is due to the challenged and control wild type samples being more similar to each other, while the mutant samples were more different in the presence of DABCOMD than when they were unchallenged. As expected, WT D S clusters between WT S and Mut D S, whereas Mut S clusters farther away (Figure 3c). Figure 3d indicates that the wild type samples (WT ML and WT S) are similar to each other, whereas the mutant sample types (Mut ML and Mut S) are significantly different from each other, causing the wild type samples to cluster. Figure 3e shows that WT D S and Mut D S cluster closer together than the mid log phase samples, but sample overlaps were not observed. Ortho PLS–DA uses component one as a predictor of class, and component two is the variation perpendicular to the first component. The observable separation is improved when all of the sample types are plotted together using ortho PLS–DA relative to 2D sPLS–DA (Figure 3f). In addition, complete separation of the smaller sample groups shown in Figure 3b–e was observable when ortho PLS–DA was used (graphs are available in the Supplementary Materials).

**Figure 4:**
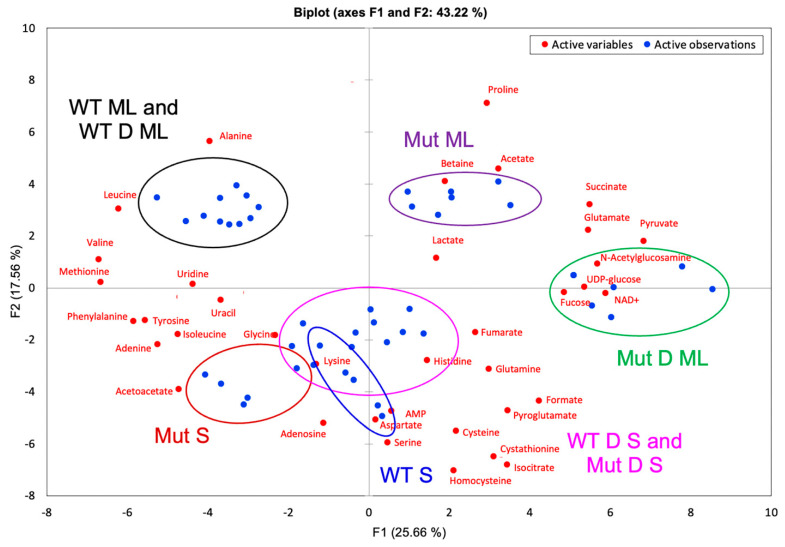
PCA biplot showing the distribution samples (blue dots) with color coordinated sample labels and circles and the distribution of metabolites (red dots).

Figure 4 shows the PCA plot of sample separation when all the samples are plotted together, accounting for 43.22% of variation. The percent variation indicates that only the first two components are needed for analysis. As expected from the hierarchical clustering (Figure 2), WT ML and WT D ML cluster together in the PCA plot, as do WT D S and Mut D S. In addition, the PCA plot shows overlap of all stationary phase clusters. The observed overlap of stationary phase clusters is due to the greater difference in the sample sets from the mid log phase. In particular, the two mutant sample types, Mut ML and Mut D ML, are sufficiently separated to cause the stationary phases to cluster closer together by comparison. The corresponding metabolites are also plotted in Figure 4. For example, N-acetylglucosamine, a peptidoglycan component, can be seen to have the strongest correlation to the DABCOMD-challenged mutant mid log phase sample type due to its significantly higher concentration in that sample type.

**Figure 5:**
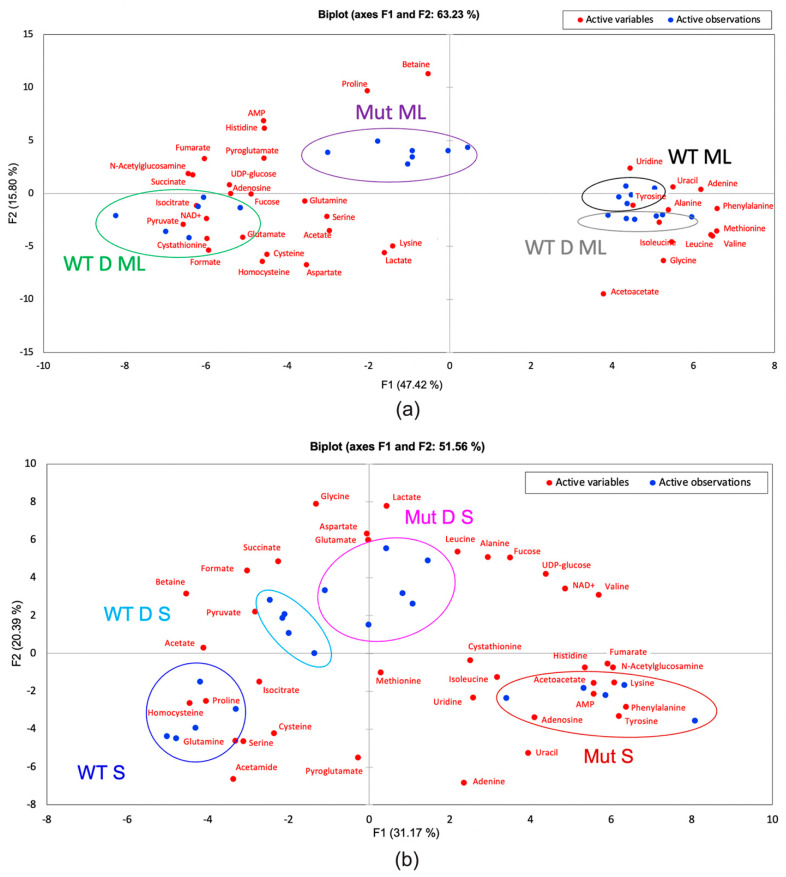
PCA biplots showing the distribution samples (blue dots) with color coordinated sample labels and circles and the distribution of metabolites (red dots). (**a**) Mid log phase samples showing WT ML and WT D ML very close together, Mut ML separated, and Mut D ML separated. (**b**) Stationary phase samples showing complete separation.

PCA biplots showing only the mid log phase (Figure 5a) or the stationary phase (Figure 5b) were quite useful to show sample type and metabolite clustering. Since the first two components account for 63.23% and 51.56% of the variance for mid log and stationary phase data comparisons, respectively, only the first two components are needed for analysis. Figure 5a shows a slight separation between the challenged and unchallenged wild type samples (WT D ML and WT ML, respectively) and a large separation between the mutant sample types Mut D ML and Mut ML. When considering metabolites, N-acetylglucosamine, for example, is indicative of the mutants because it can be seen between Mut ML and Mut D ML and is the most indicative of the DABCOMD-challenged mutant mid log phase sample type because it is closest to this sample cluster (Figure 5a). Figure 5b shows complete separation of all stationary phase sample types, with WT D S and Mut D S being clustered the closest. N-acetylglucosamine is closest to the mutant stationary phase cluster, indicating that this compound is most indicative of this sample type in the stationary phase.

**Figure 6:**
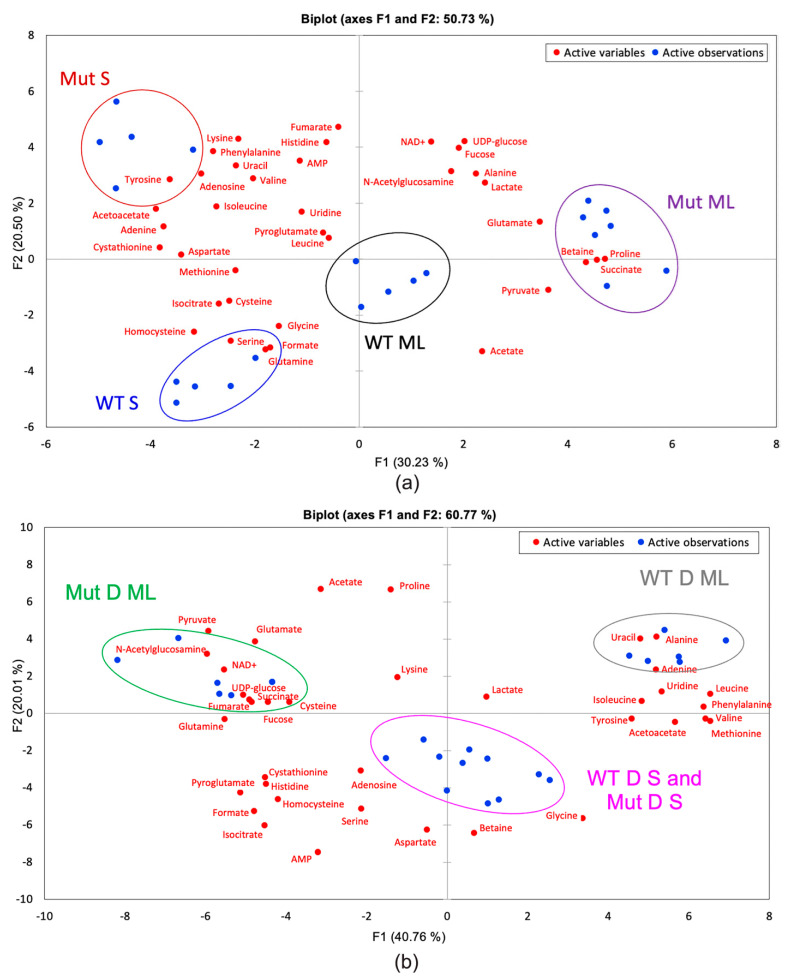
PCA biplots showing the distribution samples (blue dots) with color coordinated sample labels and circles and the distribution of metabolites (red dots). (**a**) Mut ML, Mut S, WT ML, and WT S samples showing complete separation. (**b**) DABCOMD-challenged samples showing complete separation of WT D ML and Mut D ML, and the overlap of WT D S and Mut D S.

Figure 6 shows the PCA biplot for (a) only unchallenged sample types and (b) only DABCOMD challenged sample types, accounting for 50.73% and 60.77% of the variance, respectively. Again, only the first two components are needed for analysis. Figure 6a shows complete separation of sample types (WT ML, WT S, Mut ML and Mut S) and reveals the metabolites that are most closely associated with each sample type.

For example, N-acetylglucosamine is located between the mutant sample types but is located closer to the Mut ML sample type than the Mut S sample type. This indicates that N-acetylglucosamine is important and in higher concentrations in both mutant samples in comparison to the wild type samples, but this metabolite is the most indicative of the Mut ML sample type. Figure 6b shows the overlap of the two stationary phase DABCOMD-challenged sample types, indicating that the mutant and wild type samples are more different in the mid log phase and more similar in the stationary phase. One reasonable explanation for this observation is that the organisms have settled into similar stationary phases as they have adjusted to the low levels of DABCOMD present in the media.

The metabolite identities and concentrations were entered into MetaboAnalyst 5.0 to create volcano plots of all the metabolites for pairs of sample types (Shown in Figures S8 and S9 of the Supplementary Materials). The volcano plots reveal metabolites with a *p*-value of 0.05 or less and a fold change of 1.25 or more. The volcano plots, when comparing sample sets in the mid log phase, showed that the WT ML and WT D ML sample types are the most similar, whereas Mut ML and Mut D ML sample types show significantly more differences from each other. One possible rationale for this is that the mutants react more to the low concentrations of DABCOMD in the media than the wild type, which are experiencing DABCOMD for the first time. The volcano plots using stationary phase samples overall showed less differences between sample sets. This is as expected since the samples should logically show more of a difference in the growth phase, where the bacteria need to accomplish more tasks to survive.

Very important feature scores, or variable importance projections (VIPs), in conjunction with other statistical analyses such as PCA biplots, volcano plots, pattern hunter and pathway hunter, were used to generate Table 2, Table 3, Table 4, Table 5, Table 6, Table 7, Table 8, Table 9, Table 10 and Table 11 showing important and statistically significant metabolites between paired sample types for easier analysis of the significant differences, and to display the most likely pathways where the metabolite changes occurred. Pattern Hunter was used to check the correlation of a metabolite with other metabolites found in the same pathway. If a metabolite did not correlate it was not listed in that pathway. Uracil and aspartate were listed in the alanine pathway only if Pattern Hunter showed a correlation between alanine and these amino acids (shown in the Supplementary Materials). Betaine is shown in the glycine pathway only if Pattern Hunter showed a correlation between betaine and glycine (Shown in the Supplementary Materials). Metabolites found to be in, and correlating to, more than one pathway are shown in multiple pathways, since these metabolites are probably used in multiple pathways.

### 2.1. Tables of Statistically Significant Metabolites and Their Corresponding Metabolic Pathways

Table 2, Table 3, Table 4, Table 5, Table 6, Table 7, Table 8, Table 9, Table 10 and Table 11 show pairs of sample groups for mid log and stationary phase data. Pathways involving both energy and peptidoglycan synthesis are present. An in-depth discussion of the results shown in Table 2, Table 3, Table 4, Table 5, Table 6, Table 7, Table 8, Table 9, Table 10 and Table 11 is provided in the following sections. Additional tables are provided in the Supplementary Materials, and a discussion of the implications arising from the broad trends observed when all sample sets are considered is provided in the Discussion section.

#### 2.1.1. Unchallenged Mutant versus Unchallenged Wild Types and Challenged Mutant versus Challenged Wild Types Comparisons

Table 2, Table 3, Table 4 and Table 5 provide the comparisons between the mutant and wild type and also between the DABCOMD-challenged and unchallenged sample types. Statistically significant *p*-values and fold changes in metabolites commonly found in cell membrane composition and energy production pathways were observed when comparing these sample types. Components of peptidoglycan synthesis had very large fold change differences. When comparing Mut ML to WT ML, N-acetylglucosamine had a 61.5-fold increase in concentration in the Mut ML sample type. Comparing Mut D ML to WT D ML, there was a 94.2-fold higher concentration in the Mut D ML sample type. In the stationary phase, the fold change difference of N-acetylglucosamine was lower but was still very high between the mutants and wild types, with Mut D S having a 19.4-fold increase and Mut S having a 48.7-fold increase in concentration when compared to their wild type counterparts. Mut ML displayed a 1.7-fold increase in lactate and a 3.0-fold increase in betaine when compared to WT ML, and correlated with glycine, methionine, cysteine, and N-acetylglucosamine. When comparing Mut D ML to WT D ML, there was not a significant change in betaine, but significant changes in the concentrations of aspartate, cysteine, homocysteine, methionine (VIP), and serine were observed and correlated with each other, cystathionine and N-acetylglucosamine. Mut S had a 2.4-fold decrease in betaine (VIP) and a 2.9-fold increase in lysine concentrations when compared to WT S. Glycine (VIP) and cystathionine were in higher concentrations, whereas glutamine and alanine were found in lower concentrations in Mut D S, when compared to WT D S. Nucleotide metabolism components in the mid log phase, such as adenine, uracil and uridine, had decreased concentrations, while adenosine had a higher concentration in the challenged mutant sample in comparison with the challenged wild type sample. The same was true of the unchallenged mutant samples in comparison to the unchallenged wild type samples, except changes in uridine were not observed. In the stationary phase, adenine and uracil were in higher concentrations in WT D in comparison to Mut D, whereas adenosine and uracil were at higher concentrations in WT in comparison to Mut samples. The aminoacyl-tRNA biosynthesis pathway in both the challenged and unchallenged groups contained the largest number of metabolites, with different concentrations when comparing the mutant samples with the wild type samples. A likely explanation is that aminoacyl-tRNA biosynthesis products were being diverted to peptidoglycan synthesis, since DABCOMD is a cationic quaternary ammonium compound that causes a hole to form in the membrane. It is interesting to note that, when comparing the differences between WT and Mut to WT D and Mut D, the same pathways undergo mutations, except that betaine (involved in glycine and methionine metabolism) did not change significantly in the DABCOMD-challenged samples. However, betaine can be used to make a variety of metabolites in methionine metabolism that did show significant changes, such as serine, cystathionine, cysteine, homocysteine, and methionine, as well as glycine and pyruvate. The challenged sample type comparisons (Mut D and WT D) had a greater number of changes in concentration of more metabolites than the unchallenged sample types (Mut and WT). This is reasonable since betaine would be expected to be converted and consumed to make more of the metabolite differences observed in the challenged mutant samples. Challenged mutant samples had many significant increases in the concentrations of metabolites closely correlated with betaine in comparison to the unchallenged mutant samples. More research is needed to determine what additional pathway changes may be causing the betaine concentration changes.

The DABCOMD-challenged samples (Mut D vs. WT D) showed changes in a greater number of metabolites per pathway when compared to the unchallenged samples (Mut vs. WT). When comparing Mut ML to WT ML and Mut D ML to WT ML, energy-related metabolites found in the citric acid cycle and pyruvate metabolism, such as fumarate, isocitrate, succinate, formate and pyruvate, were found to be in excess in the mutant sample types. Acetoacetate, the exception, was in excess in the wild type samples. Energy metabolites such as AMP and NAD^+^ were in higher concentrations in both the challenged and unchallenged mutant sample types relative to their wild type counterparts (6.8-fold and 37-fold increases for AMP and NAD^+^, respectively, for Mut D ML relative to wild type WT D ML). In the unchallenged samples, the mutant samples (Mut ML) had a 12.3-fold increase in NAD^+^ and a 9.1-fold increase in AMP concentrations when compared the unchallenged wild type samples (WT ML). This increase in the concentration of NAD^+^ in the challenged samples was observed in the stationary phase as well.

#### 2.1.2. Challenged Wild Type versus Unchallenged Wild Type Comparisons

The sample comparison set that included the fewest observable changes was the comparison between the wild type samples grown in the presence and absence of DABCOMD, which is shown in Table 6 and Table 7. The main differences were an increase in alanine, glutamate, and lactate concentrations, and a decrease in cystathionine and glutamine. These metabolites are commonly found components of peptidoglycan synthesis. Adding a small of amount of the DABCOMD to the wild type (which had never been exposed to DABCOMD before) likely caused enhanced mutations for protection of their peptidoglycan layer through common substitutions to the pentapeptide side chains. The growth curves were comparable for these two sample types (shown in the Supplementary Materials), again suggesting that although changes to the peptidoglycan composition in response exposure to low levels of DABCOMD by the wild type samples did cost some energy and cause some membrane disruption, dramatic metabolic changes did not occur. The only component of nucleotide metabolism that had a significant observable change was the decreased concentration of uridine in the challenged wild type samples in comparison with the unchallenged wild types in the stationary phase. In the stationary phase, the greatest number of observable changes were in amino acids involved in aminoacyl-tRNA biosynthesis, and changes in the concentrations of metabolites involved in the same types of pathways (energy production and peptidoglycan synthesis) as were observed in the mid log phase.

#### 2.1.3. Challenged Mutant versus Unchallenged Mutant Comparisons

Adding low levels of DABCOMD to the mutant samples created a significant difference in metabolite concentrations in multiple pathways associated with peptidoglycan synthesis and energy related pathways, as shown in Table 8 and Table 9. In addition, the growth curve of the challenged samples was significantly slower than that of their unchallenged mutant counterparts (Figure S2 in the Supplementary Materials). Since the mutants had been previously exposed to DABCOMD, when DABCOMD was added to their media again, even in low levels, it caused them to fortify their defenses against the cationic antimicrobial agent to such an extent that their rate of growth was slow. The slower growth rate and the identification of spent energy molecules in the metabolite analysis can both be attributed to increased membrane disruption and fortification. The import of essential nutrients is reduced while extra energy is also needed to induce membrane fortifications. In the mid log phase, the challenged samples had higher concentrations of the following energy metabolites: NAD^+^, succinate, isocitrate, acetate, formate and pyruvate. Multiple metabolites that can be used in peptidoglycan synthesis also had higher concentrations in the challenged mutant samples, including cystathionine, glycine, glutamate, and glutamine. Alanine, on the other hand, had a higher concentration in the unchallenged mutant samples. Betaine had a negative correlation with glycine, aspartate, cystathionine, cysteine, homocysteine and pyruvate in the mid log phase, while exhibiting a positive correlation to glycine and aspartate and a negative correlation to N-acetylglucosamine levels in the stationary phase. Alanine had a positive correlation with uracil and methionine, and a negative correlation with N-acetylglucosamine, cysteine and cystathionine levels; aspartate, cysteine and homocysteine concentrations had a positive correlation with cystathionine levels. Nucleotide metabolism components in the mid log phase, such as adenine and uracil had decreased concentrations in the challenged mutants in comparison with the unchallenged mutant sample types. The same is true of the stationary phase, except adenosine was also observed to be in lower concentrations in the challenged mutant samples in comparison to the unchallenged mutant samples. In the stationary phase, glycine (VIP), glutamate (VIP) and lactate were found in higher concentrations in the challenged samples, whereas lysine and N-acetylglucosamine were observed in higher concentrations in the unchallenged samples. Energy pathway related metabolites such as succinate, acetate and formate were VIPs and were found in higher concentrations in the challenged samples. Changes in the concentrations of components of aminoacyl-tRNA biosynthesis were observed in both the mid log and stationary phases as well.

#### 2.1.4. Challenged Wild Type versus Unchallenged Mutants Comparisons

The comparison of the DABCOMD-challenged wild type (never been exposed to DABCOMD before) to the unchallenged mutant samples (mutated in the presence of DABCOMD) is shown in Table 10 and Table 11. These samples always cluster separately in the mid log phase (Figure 2a, Figure 3a,b, Figure 5, Figures S4a,d, S5a,b, S6 and S7a,b) and almost cluster separately in the stationary phase (Figure 2a, Figure 3c, Figure 5b, Figures S4a,e, S5a,c and S7a,c). The only instances where these sample groups cluster together is in the stationary phase was when all eight sample types are plotted together (Figure 3a, Figure 4 and Figure S6). The differences between the stationary phases are smaller than those in the mid log phase, thus causing the stationary phases to cluster closer together when they are all plotted on the same graph. The unchallenged mutant samples and the challenged wild type samples clustering into unique sample types with many metabolite concentration differences (24 and 23) and large fold change differences (61.5, 12.4 and 9.1-fold in the mid log and 48.7, 11.1 and 8.9-fold in the stationary in the mutant samples), strongly suggests that the mutant samples truly are mutated variants of the original bacterial strain. Challenged wild type samples and unchallenged mutant samples, or the challenged wild type samples and challenged mutant samples, would likely cluster together if the DABCOMD only temporarily activated different pathways. Unchallenged mutant mid log samples had higher concentrations of NAD^+^, AMP, fumarate, succinate, and pyruvate than the mid log challenged wild type samples. The unchallenged mutant samples in the mid log phase also had higher concentrations in N-acetylglucosamine and cystathionine when compared to challenged wild type samples in the mid log phase. Nucleotide metabolism components in the mid log phase had decreased concentrations of adenosine and higher concentrations of adenine and uracil in the challenged wild type samples, in comparison with the unchallenged mutant samples. In the stationary phase, the unchallenged mutant samples had higher concentrations of these metabolites relative to the metabolite concentrations found in the challenged wild type samples. These changes were also observed in the stationary phase, with the unchallenged mutant samples having a fold increase of 11.1 in NAD^+^, 8.9 in fumarate, 2.3 in AMP and 48.7 in N-acetylglucosamine relative to the challenged wild type samples. Lysine, N-acetylglucosamine and cystathionine were in excess in Mut S. Betaine had a 3.6-fold change decrease in WT D ML in comparison to Mut ML and negatively correlated to glycine, aspartate, cystathionine, cysteine, homocysteine, and pyruvate, while betaine had a 3.2-fold increase in WT D S when compared to Mut S and negatively correlated to cystathionine and N-acetylglucosamine. A positive correlation to homocysteine, glycine and pyruvate was also observed for betaine in WT D S compared to Mut S. In both the mid log and the stationary phases, both WT D and Mut had significant changes in the metabolites for energy pathways, aminoacyl-tRNA biosynthesis, peptidoglycan synthesis and associated pathways.

## 3. Discussion

When *B. cereus* was grown in the presence of DABCOMD and compared to wild type *B. cereus* to obtain the eight sample sets described above (Table 1), statistically significant concentration changes in metabolites likely involved in energy-related and cell wall composition-related pathways were observed. The largest fold changes occurred in metabolites found in peptidoglycan synthesis and energy-related pathways. The most compelling outcomes from this metabolomics study and the most likely implications of the observed trends are described below.

### 3.1. Comparisons between Metabolite Levels

Since in the stationary phase the cells are not rapidly dividing any more, the bacteria are likely settled into a fortified membrane structure and have decreased metabolism. For this reason, smaller differences between all the sample type metabolite concentrations were observed in the stationary phase than in the mid log phase [56].

In the mid log phase, glycine levels were significantly different between all sample type comparisons, except between the DABCOMD-challenged and unchallenged wild type samples. In the stationary phase, glycine levels were different between all sample type comparisons, except between the challenged and unchallenged wild type samples and between the unchallenged mutant and unchallenged wild type samples. Betaine concentrations differed in the mid log phase between the challenged and unchallenged mutant samples, between unchallenged mutant and wild type, and between the challenged wild type and unchallenged mutant samples. In the stationary phase, betaine concentration levels differed between the WT D and WT samples, the WT D and Mut samples, and between the Mut D and Mut samples. Betaine was correlated not only with pyruvate (energy related molecule), glycine and cystathionine (peptidoglycan related molecules), but was also correlated with many components of methionine metabolism. When comparing any of the mutant samples to the wild type samples in the mid log phase, a significant change in the lactate concentration was observable. In the stationary phase, the concentration of lactate changed when comparing Mut D to Mut, Mut D to WT, and WT D to Mut. In the mid log phase, cystathionine was in higher concentrations in the mutant sample types in comparison to the wild type samples. Interestingly, the highest concentration of cystathionine was detected in the challenged mutant samples, and the lowest was found in the challenged wild type. In the stationary phase, there was a difference between the challenged wild type samples and both types of mutant samples (challenged and unchallenged), with the mutant samples having the higher concentration of cystathionine. Cysteine followed the same pattern as cystathionine, with the mutant samples having a higher concentration than the wild type, and the challenged mutant samples having the highest concentration in the mid log phase. In the stationary phase, the challenged mutant samples had a lower concentration of cysteine than the challenged wild type samples, and significant changes in cysteine levels between other paired sample types were not observed. In the mid log phase, methionine had a higher concentration in mutant samples in comparison to their wild type counterparts, except challenged wild type samples were higher in concentration in comparison to the unchallenged mutant samples. There was not an observed difference in the concentration of methionine in the stationary phase. For homocysteine in the mid log phase, the only sample type comparisons that showed significant differences in concentration were between the challenged mutant samples and all other sample types they were compared against, with the challenged mutant samples having the highest concentration of homocysteine. In the stationary phase, the only difference in homocysteine levels was between the challenged wild type samples and the unchallenged mutant samples, with the unchallenged mutant samples having a lower concentration of homocysteine in comparison to the challenged wild type samples. In the mid log phase, aspartate had a higher concentration when comparing the challenged mutant samples to all sample types they were compared against. In the stationary phase, aspartate had a significantly higher concentration in the challenged mutant samples than in their challenged and unchallenged wild type sample counterparts. In the mid log phase, the glutamate concentration was higher in all the mutant samples than their wild type counterparts, with all challenged samples having higher concentrations of glutamate than their unchallenged counterparts. This differed from the stationary phase, where a difference in glutamate levels was only observed when comparing the challenged sample types to the unchallenged samples, with the challenged sample types having the higher glutamate concentration. With glutamine in the mid log phase, the challenged mutant samples had the highest concentration while the challenged wild type samples had the lowest concentration. The mutant samples had a higher concentration of glutamine than the wild type samples. In the stationary phase, however, the wild type samples had a higher concentration of glutamine than the challenged mutant samples did, and the unchallenged wild type samples had a higher concentration of glutamine than was present in the challenged wild type samples. Significant increases in lysine concentration were observed in the stationary phase in the unchallenged mutants in comparison to the unchallenged wild type samples. There was not an observable difference between the wild type sample types (WT D to WT) or between the DABCOMD-challenged sample types (Mut D to WT D).

**Figure 7:**
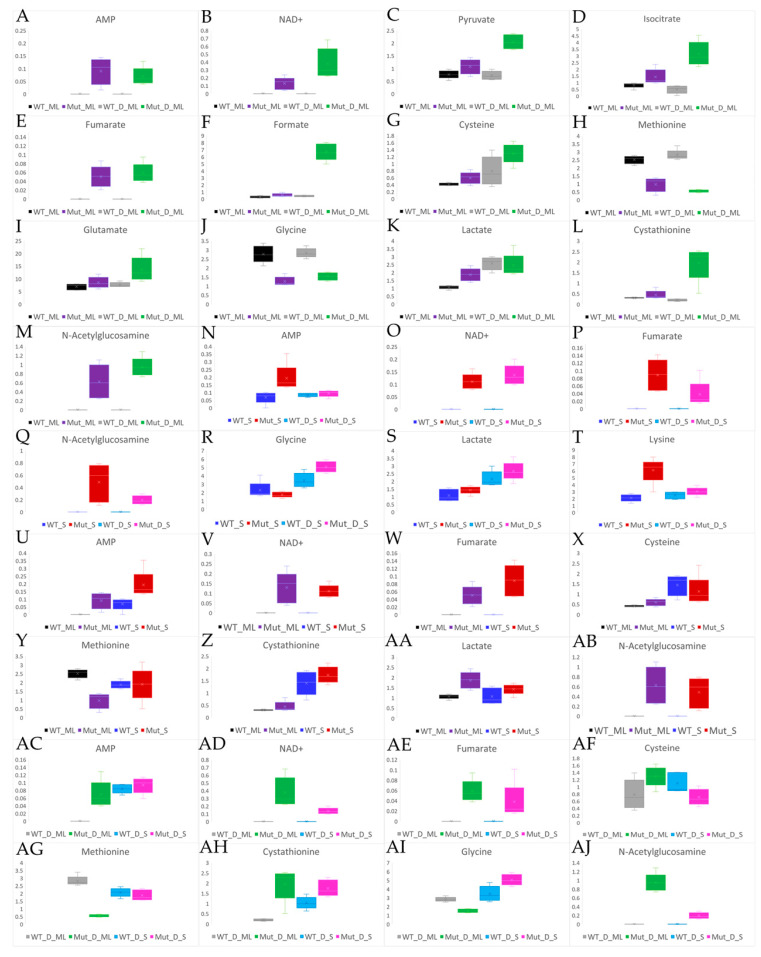
Box and whisker plots to show the differences in fold changes and the fold change ranges for example metabolites involved in peptidoglycan synthesis, membrane permeability and energy associated pathways. (**A**–**M**) Mid Log Phase Sample Comparisons. (**N**–**T**) Stationary Phase Sample Comparisons. (**U**–**AB**) Unchallenged Sample Comparisons. (**AC**–**AJ**) Challenged Sample Comparisons.

Pyruvate concentrations displayed a significant change during the mid log phase for all sample type comparisons, except when the challenged and unchallenged wild type samples were compared. When comparing low energy metabolites such as NAD^+^ and AMP in the mid log phase, the mutant samples had a significantly higher concentration of these metabolites than the wild type samples, and the challenged mutant samples had a higher concentration than their unchallenged mutant counterparts. In the mid log phase, the mutant samples had higher concentrations of isocitrate than their wild type counterparts. In the unchallenged wild type samples, isocitrate had a higher concentration than was found in the challenged wild type samples, whereas unchallenged mutant samples had a lower concentration of isocitrate than the challenged mutant samples. In the stationary phase, the unchallenged wild type samples had a higher concentration of isocitrate than the unchallenged mutant samples, and challenged wild type samples had higher concentrations than the challenged mutant samples. Formate in the mid log phase had higher concentrations in the all the mutant sample types in comparison to the wild type samples. The largest difference in formate concentrations was seen when comparing the challenged mutant samples to the challenged wild type samples. This is because there was not a significant difference between the wild type samples (challenged and unchallenged), but the challenged mutant samples had a higher concentration than the unchallenged mutant samples. There was less of a difference between the formate levels in the stationary phase sample types than in the mid log phase sample types. Figure 7 displays the average and range of fold changes in key energy metabolites in comparisons between the wild type, the mutants, the unchallenged and the challenged *B. cereus* samples.

### 3.2. Significance of Metabolite Changes

Energy pathway-related metabolites such as succinate, acetate and formate were VIPs found in higher concentrations in the challenged samples. Low energy metabolites, such as NAD^+^ and AMP, were found in higher concentrations between the mutant samples than their wild type counterparts, and higher between the challenged mutant samples than the unchallenged mutant samples, while there was not a significant difference between the challenged and unchallenged wild type samples. The mutant samples, especially the DABCOMD-challenged mutant samples, produce more metabolites to adapt to being grown in low levels of the antimicrobial compound. Growth of the mutants in DABCOMD results in increased energy requirements and a slower growth curve since the level of nutrients imported into the cell is reduced because of membrane fortification and disruption [39].

Significant changes in metabolites likely involved in aminoacyl-tRNA biosynthesis were observed when comparing the wild type and mutant samples, DABCOMD-challenged and unchallenged, in both the mid log and stationary phases. These changes were most likely due to diversion of components of aminoacyl-tRNA biosynthesis to peptidoglycan synthesis and antibiotic resistance [17]. The mutant sample types generally had a lower concentration of nucleotide metabolites than their wild type counterparts. Some of these components were likely used in aminoacyl-tRNA biosynthesis. Changes were observed in the concentrations of many components of peptidoglycan synthesis that are involved in cell wall composition and frequently change in response to environmental stressors (like an antimicrobial being added to their growth media), such as lactate, glutamate, lysine, glycine, and cystathionine. Changing the composition of the pentapeptide side chain of peptidoglycan is a common method for increasing bacterial resistance to antimicrobials [10,16,19,21,57]. Lysine, a positively charged amino acid, is commonly associated with incorporation into the peptidoglycan layer to increase the overall positive charge. Lysine substituted into the pentapeptide side chain of peptidoglycan has been shown to decrease the permeability and effectiveness of cationic antimicrobials [17]. Incorporation of glycine into peptidoglycan causes crosslinking and increases rigidity [17,22]. It has been shown that increased crosslinking (glycine incorporation into peptidoglycan) is directly related to an increase in bacterial survival rates [23]. Mengin-Lecreulx et al. have previously shown that bacteria incorporate the cystathionine that was added to their media into their peptidoglycan layer. When cystathionine is incorporated into peptidoglycan, this truncates the pentapeptide side chain by binding in the number three spot and the D-ala-D-ala tail is never attached [57,58]. Another metabolite that truncates the polyalanine tail is lactate [16,19], which forms a D-ala-D-lac bond to change the terminal residue from alanine to lactate. If more lactate is needed, pyruvate can be reduced to D-lactate [16,19]. Lacking the polyalanine tail of the peptidoglycan by incorporating different metabolites is a common method used by bacteria to develop antimicrobial resistance [16,17,19,23]. The largest change observed in this study occurred in the levels of N-acetylglucosamine, with the mutant samples having the highest concentration. These changes were observed in the mid log phase when comparing any of the mutant samples to any of the wild type samples (DABCOMD challenged and unchallenged), but not when comparing the wild type samples to each other (DABCOMD-challenged and unchallenged) or the mutant samples with each other (DABCOMD-challenged and unchallenged). In the stationary phase, there was less of a difference in the concentration of N-acetylglucosamine when comparing any of the mutant sample types to any of the wild type samples and comparing Mut D S to Mut S than there was in the mid log phase. Figure 7 displays the fold change average and range for key observed metabolites involved in protecting *B. cereus* from DABCOMD. The significant changes in N-acetylglucosamine and other metabolites involved in peptidoglycan synthesis adds support to the theory that the mutants change the composition of their peptidoglycan layer to protect themselves from the positive charge on DABCOMD, thus reducing their likelihood that catastrophic membrane hole formation can occur.

### 3.3. Metabolomic Comparison of Gram-Positive and Gram-Negative Bacterial Exposure to DABCOMD

DABCOMD, a multivalent membrane disruptor, has been shown to work on both Gram-positive and Gram-negative bacteria without acquisition of resistance [27]. Membrane disruptors, especially multivalent structures are especially intriguing because they also inadvertently cause energy and nutrient import disruption [39].

Bacteria cannot easily change their membranes to such an extent that they become resistant to multivalent membrane disruptors, since membrane disruptors are generally attracted to the large negative charge of the membrane [40,41]. The effects of DABCOMD on the Gram-negative bacteria, *Escherichia coli* (*E. coli)*, have been previously reported [59]. No differences in growth curves were observed when comparing the wild type and mutated *E. coli*, whereas the mutated *B. cereus* samples had a slower growth and did not reach as high of an optical density in the stationary phase. Not as many upstream pathways to peptidoglycan synthesis and aminoacyl-tRNA biosynthesis were observed in the *E. coli* samples in comparison to the *B. cereus* samples. The fold changes for the concentrations in observed metabolites were also larger in the *B. cereus* results in comparison to the *E. coli* results, especially for metabolites associated with peptidoglycan synthesis and energy related pathways. This makes sense because Gram-negative bacteria contain about 10% peptidoglycan in their cell membranes, whereas Gram-positive bacteria contain up to 70% peptidoglycan in their cell walls [20]. Since Gram-positive bacteria (*B. cereus)* have a thicker peptidoglycan layer, more changes can be observed in comparison to the Gram-negative bacteria (*E. coli*). Larger differences in observed energy related metabolites are also reasonable for Gram-positive bacteria, since changes to the higher concentration of peptidoglycan in the phospholipid membrane (Gram-positive) would require more energy, more rigidity, and more membrane disruption with nutrient import. The greater observed disruption of nutrient import is as expected for *B. cereus* due to its cell wall and larger concentration of peptidoglycan, and this is likely responsible for the slower growth curve that also does not reach the regular stationary phase optical density. The membrane disruption properties of DABCOMD are especially evident when observing that the challenged mutant *B. cereus* samples had the slowest overall growth curve, had the lowest stationary phase density, and was associated with higher N-acetylglucosamine production and spent energy molecules in comparison to the unchallenged mutant *B. cereus* samples (which did not have as slow of a growth curve or as much of a fold change for peptidoglycan synthesis and spent energy pathways) and especially in comparison to the mutated *E. coli* samples (which did not have any observable change in growth curve and did not have as many peptidoglycan synthesis and spent energy changes). Overall, DABCOMD is a multivalent membrane disruptor that is difficult for either Gram-positive or Gram-negative bacteria to develop resistance to, since this antimicrobial compound contains multiple attached positive charges that are attracted to the negatively charged phospholipids of bacteria. In the attempt to develop resistance to DABCOMD, both Gram-positive (*B. cereus*) and Gram-negative (*E. coli*) bacteria were observed to fortify their membranes by altering their peptidoglycan layers, consequently incurring more energy costs and disruption of nutrient import.

## 4. Materials and Methods

### 4.1. Samples

**Figure 8:**
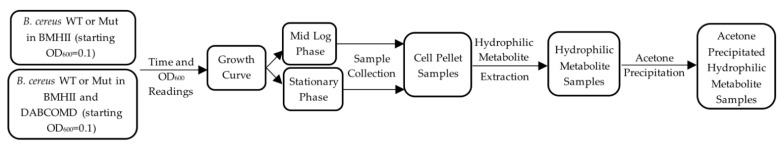
Overview of the protocol from the start of the cultures to the hydrophilic metabolite samples ready to be pelleted, frozen at −80 °C, and put in NMR buffer. The processes for unchallenged and challenged samples are the same except for the inclusion of DABCOMD in the challenged sample media.

FDA strain PC1 213 *B. cereus* (ATCC 11778) was used for all the wild type (WT) samples and was mutated in the presence of DABCOMD as previously described in VanKoten et al. 2016 to create the mutant (Mut) samples. Unchallenged samples of both WT and Mut bacteria were grown in Brodo Mueller Hinton II Media (BMHII), the challenged samples (WT D and Mut D) were grown in BMHII with 0.37 μM/L of DABCOMD added to the media (this is 33% of the MIC value [27]). All other procedures were the same between the two groups. There were eight different sample types: unchallenged wild type mid log phase samples (WT ML), challenged wild type mid log phase samples (WT D ML), unchallenged wild type stationary phase (WT S), challenged wild type stationary phase (WT D S), unchallenged mutant mid log phase (Mut ML), challenged mutant mid log phase (Mut D ML), unchallenged mutant stationary phase (Mut S), and challenged mutant stationary phase (Mut D S). A detailed description of how the samples were grown, collected, and standardized is provided in the Supplementary Materials. A visual overview for the procedures is shown in Figure 8.

### 4.2. Metabolite Extraction Procedures

#### 4.2.1. Hydrophilic Extraction

Note: All H_2_O used was Millipore and all reagents used were cold (kept in the refrigerator).

Sample cell pellets were thawed on ice and in glass test tubes. Methanol (800 μL) and Millipore water (170 μL) were pipetted into each test tube and the samples were vortexed. The sonicator was used on each test tube for 5 min followed by vortexing. After chloroform (800 μL) and Millipore water (400 μL) were added to each test tube, they were vortexed and incubated for 15 min on ice. The samples were centrifuged for 15 min at 3500 rpms. After the aqueous layer was pipetted into a sterile Eppendorf tube, the samples were dried on a speed vacuum. The hydrophilic pellets were frozen at −80 °C [55,59,60] and references therein.

#### 4.2.2. Acetone Precipitation

The frozen metabolite pellets were thawed on ice. D_2_O (250 μL) and 1250 μL acetone were added to the pellets, then they were frozen overnight at −80 °C. The pellets were thawed on ice, causing them to become cloudy with precipitated proteins. After the samples were thawed, they were centrifuged for 30 min at 2000 rpms. The liquid was pipetted into capped Eppendorf tubes, and the protein pellet was discarded. A speed vacuum dried the samples, and the resulting hydrophilic pellets were frozen at −80 °C [55,59,60] and references therein.

### 4.3. NMR Sample Preparation and Data Processing

Note: NMR grade D_2_O was always used.

#### 4.3.1. NMR Buffer Preparation

Imidazole (27.230 g) was added to 4.0 mL of D_2_O to make an imidazole stock solution. A DSS stock solution was created by adding 21.23 mg of 3-(Trimethylsilyl)propane-1-sulfonate sodium salt (DSS) to 4.0 mL of D_2_O. A sodium phosphate buffer stock solution was made by adding 0.345 g NaH_2_PO_4_^.^H_2_O, 0.355 g Na_2_HPO_4_ (anhydrous) and 0.040 g NaN_3_ to 5.0 mL of D_2_O. The stock solutions of imidazole (60 µL), sodium phosphate (1500 µL), and DSS (300 µL) were added to 28,140 µL of D_2_O to create a 30 mL D_2_O buffer solution [55,59,60] and references therein.

#### 4.3.2. Sample Preparation

Metabolite samples were prepared by taking the cell pellets obtained after acetone precipitation, placing them on ice, and adding 700 µL of the NMR buffer. The mixture was vortexed and transferred to capped glass NMR tube. The Supplementary Materials contain additional details regarding sample preparation.

#### 4.3.3. Data Acquisition

Spectra were collected for each hydrophilic metabolite sample by using Topspin software (Bruker version 3.6) with a SampleJet^TM^ automatic sample loading system on a Bruker Avance III 600 MHz NMR equipped with a 5 mm triple resonance (^1^H, ^15^N, ^13^C) liquid-helium cooled TCI NMR CryoProbe at 298 K. A Bruker-supplied excitation sculpting (ES)-based ‘zgesgp’ pulse sequence was used to acquire 1D ^1^H NMR spectra for all samples. All the NMR spectra were recorded using a ^1^H spectral window of 7211.538 Hz, 256 scans, 64K data points and a dwell time interval of 69.33 µsec between points, resulting in a spectrum acquisition time of 4.54 s. A recovery delay (D1) time between acquisitions of 2 s was used, amounting to a total relaxation recovery time of 6.5 s between scans. A Fourier transformation was used on the spectra. H_2_O and DSS resonances were used to phase the spectra.

#### 4.3.4. Data Analysis

Chenomx NMR Suite 8.4 software [61] was used to profile the spectra. The identity and concentration of the metabolites in each sample were obtained by using Chenomx profiler’s list of metabolites to generate a fitted metabolite spectrum. Chenomx is unable at this point to identify all peaks observed in the NMR spectra. The constant sum method was used to standardize the concentrations of the metabolites identified in each sample [62]. The concentration of each individual metabolite was divided by the total concentration of all metabolites measured. This method works on the premise that the concentration of each individual metabolite changes relative to the concentration of the total sample [62]. XLSTAT hierarchical clustering, principal component analysis (PCA) and PCA biplot were used to analyze the standardized metabolite concentrations for each sample [63]. A comma delineated form (CSV) of the standardized metabolite data were uploaded to MetaboAnalyst 5.0 for statistical analysis [64] and references therein. MetaboAnalyst 5.0 was used to generate volcano plots, 2D and 3D sparse partial least squares discriminate analysis (sPLS–DA), orthogonal partial least squares discriminate analysis (ortho PLS–DA), Pattern Hunter and PLS–DA very important features (VIP) data to determine correlations, and statistical significance of observed metabolite changes in each sample type. PCA, 2D and 3D sPLS–DA, ortho PLS–DA were used to identify outlier samples, demonstrate sample separation, and identify metabolite correlations to sample types. Volcano plots were used to determine which metabolites had significant fold changes and *p*-values (0.5 or less), while Pattern Hunter was used to ascertain correlations between the metabolites in each paired sample type. The metabolites that were determined to be statistically significant were uploaded into the MetaboAnalyst Pathway Analysis tool and KEGG pathways [65] and references therein. All these statistical analyses were used in conjunction to ascertain the most probable metabolomic pathway changes that occurred in the mutant samples and with the DABCOMD-challenged sample types.

## 5. Conclusions

Novel antimicrobials with new scaffolds are needed to assuage the increasing tide of ever increasing antibiotic resistant bacteria. Multivalent antimicrobials have the potential to exhibit increased efficacy relative to their monovalent counterparts. The C_16_-DABCO and mannose functionalized dendrimer (DABCOMD) was used to study the effects of multivalent quaternary ammonium antimicrobials. The mutant samples were previously mutated in the presence of DABCOMD. In addition, samples were challenged with a low dose of DABCOMD in their growth media to generate samples for comparison to samples grown in unchallenged media. Metabolite profiles during both the mid log and stationary phases were undertaken using ^1^H NMR hydrophilic metabolite analysis to discern metabolite identifications and concentrations. Many metabolites commonly associated with energy associated pathways, peptidoglycan synthesis and related pathways were observed. The stationary phase had significantly less changes in metabolite concentrations between paired sample types than the mid log phase. An example of a stationary phase significant change is in the concentration of lysine, of which higher concentrations were associated with the mutant sample types. Since lysine is a positively charged amino acid associated with decreasing the efficacy of positively charged antimicrobials, the observed changes in lysine concentrations are highly consistent with cell wall alternations that would be expected for these studies. The largest observed metabolite concentration change (up to 94.2-fold) was in N-acetylglucosamine. Higher N acetylglucosamine levels were associated with the mutant sample type, especially the challenged mutant sample type in comparison to their wild type counterparts. N-acetylglucosamine is a major component of peptidoglycan synthesis and is involved in cell membrane fortification and survivability. The mutant sample type, especially the challenged mutant samples, were associated with significantly more spent energy molecules (up to 37-fold increase in NAD^+^), and a slower growth curve. The slower growth and association with spent energy molecules of the mutants, especially the challenged mutants, was most likely due to membrane fortification slowing the rate of nutrient entry into the cell. Moreover, making these fortifications would be energetically costly. The differences in energy requirements between the mutant and wild type samples and DABCOMD challenged and unchallenged sample types were likely less significant in the stationary phase than the mid log phase, because the bacteria were no longer rapidly dividing, were changing their peptidoglycan layers less frequently, and were decreasing their metabolism.

Overall, there was a larger difference between the challenged wild type/mutant sample paring than the unchallenged wild type/mutant sample paring. The smallest difference was between the challenged and unchallenged wild type samples, while there was a large difference between the challenged and unchallenged mutant samples. The data from analyzing NMR hydrophilic metabolite concentrations from challenged versus unchallenged sample types demonstrated that the challenged mutant samples grew more slowly, had more spent energy related metabolites and had more and larger concentration changes in metabolites associated with peptidoglycan synthesis and related pathways than their unchallenged counterparts. The mutant *B. cereus* samples, especially those re-exposed to the antimicrobial, likely change their peptidoglycan composition to protect their cell walls from the large positive charge on DABCOMD. This change in the peptidoglycan cause them to require more energy, have a greater extent of membrane disruption of nutrient import, and grow more slowly than the wild type samples.

## Figures and Tables

**Table 1:** Sample sets used in this study.

Sample Group ^1^	Abbreviation
Wild Type Mid Log	WT ML
Wild Type Stationary	WT S
Wild Type DABCOMD Mid Log	WT D ML
Wild Type DABCOMD Stationary	WT D S
Mutant Mid Log	Mut ML
Mutant Stationary	Mut S
Mutant DABCOMD Mid Log	Mut D ML
Mutant DABCOMD Stationary	Mut D S

^1^ The color-coding of the sample sets shown here is used throughout this manuscript.

**Table 2:** Fold change of statistically significant metabolites in their corresponding metabolic pathways for the mid log phase of mutant versus wild type.

Mut ML versus WT ML					
Indicated Pathway	Metabolite	Fold Change ^1,2^	Indicated Pathway	Metabolite	Fold Change
Citric Acid Cycle	Acetoacetate	−2.8	Alanine Metabolism	Uracil ^4^	−1.2
	Fumarate ^3^	+4.9			
	Isocitrate	+1.7	Nucleotide Metabolism	Adenine	−1.5
	NAD+ ^3^	+12.3		Adenosine	+2.9
	Succinate	+1.4		Uracil ^4^	−1.2
Pyruvate Metabolism	Formate	+1.7	Aminoacyl-tRNA Biosynthesis	AMP ^3^	+9.1
	Pyruvate	+1.4		Glutamine ^4^	−1.6
Peptidoglycan Synthesis	Glutamine ^4^	−1.6		Histidine	+6.2
	Glycine ^4^	−2.2		Isoleucine	−4.6
	Lactate	+1.7		Leucine	−3.4 (VIP)
	N-Acetylglucosamine ^3^	+61.5		Phenylalanine	−2.0 (VIP)
Methionine Metabolism	Betaine ^4^	+3.0 (VIP)		Proline	+1.8 (VIP)
	Cysteine	+1.4		Tyrosine	−1.5
	Methionine	−2.6		Valine	−3.8 (VIP)
Glycine Metabolism	Betaine ^4^	+3.0 (VIP)			
				

^1^ A positive fold change is indicative of a higher concentration in the mutant. ^2^ The metabolites with VIP next to them were determined to be very important features by the PSL-DA. ^3^ These metabolites had concentrations lower than 0.01 for the sample with the lowest concentration. ^4^ These metabolites are shown in multiple pathways where a correlation was shown by Pattern Hunter.

**Table 3:** Fold change of statistically significant metabolites in their corresponding metabolic pathways for the mid log phase of DABCOMD challenged mutant versus DABCOMD challenged wild type.

Mut D ML versus WT D ML					
Indicated Pathway	Metabolite	Fold Change ^1,2^	Indicated Pathway	Metabolite	Fold Change
Citric Acid Cycle	Acetoacetate	−1.6	Alanine Metabolism	Aspartate ^4^	+1.8
	Fumarate ^3^	+5.8		Uracil ^4^	−7.7 (VIP)
	Isocitrate	+6.1 (VIP)	Nucleotide Metabolism	Adenine	−2.1
	NAD+ ^3^	+37.0		Adenosine	+3.0
	Succinate	+2.2 (VIP)		Uracil ^4^	−7.7 (VIP)
Pyruvate Metabolism	Acetate	+1.3 (VIP)		Uridine	−4.4
	Formate	+16.3 (VIP)	Aminoacyl-tRNA Biosynthesis	Alanine ^4^	−2.1 (VIP)
	Pyruvate	+2.9		AMP ^3^	+6.8
Peptidoglycan Synthesis	Alanine ^4^	−2.1 (VIP)		Aspartate ^4^	+1.8
	Glutamate ^4^	+1.7 (VIP)		Glutamate ^4^	+1.7 (VIP)
	Glutamine ^4^	+4.1 (VIP)		Glutamine ^4^	+4.1 (VIP)
	Glycine ^4^	−1.6		Glycine ^4^	−1.6
	N-Acetylglucosamine ^3^	+94.2		Histidine ^3^	+8.7
Methionine Metabolism	Aspartate ^4^	+1.8		Isoleucine	−5.5
	Cysteine	+1.8		Leucine	−11.3 (VIP)
	Homocysteine	+2.7		Phenylalanine	−4.0 (VIP)
	Methionine	−5.1 (VIP)		Tyrosine	−3.4
	Serine	+1.7		Valine	−11.2 (VIP)

^1^ A positive fold change is indicative of a higher concentration in the mutant. ^2^ The metabolites with VIP next to them were determined to be very important features by the PSL-DA. ^3^ These metabolites had concentrations lower than 0.01 for the sample with the lowest concentration. ^4^ These metabolites are shown in multiple pathways where a correlation was shown by Pattern Hunter.

**Table 4:** Fold change of statistically significant metabolites in their corresponding metabolic pathways for the stationary phase of mutant versus wild type.

Mut S versus WT S					
Indicated Pathway	Metabolite	Fold Change ^1,2^	Indicated Pathway	Metabolite	Fold Change
Citric Acid Cycle	Acetoacetate	+1.8	Nucleotide Metabolism	Adenosine	+3.2
	Fumarate ^3^	+8.9		Uracil ^4^	+2.2 (VIP)
	NAD+ ^3^	+11.1	Aminoacyl-tRNA Biosynthesis	Alanine ^4^	+1.4 (VIP)
Pyruvate Metabolism	Acetate	−3.9 (VIP)		AMP	+3.3
	Formate	−7.3 (VIP)		Histidine ^3^	+21.6
Peptidoglycan Synthesis	Alanine ^4^	+1.4 (VIP)		Leucine	+1.8
	Lysine	+2.9 (VIP)		Phenylalanine	+3.1 (VIP)
	N-Acetylglucosamine ^3^	+48.7		Proline	−1.7 (VIP)
Glycine Metabolism	Betaine	−2.4 (VIP)		Tyrosine	+1.7
				Valine	+3.1 (VIP)

^1^ A positive fold change is indicative of a higher concentration in the mutant. ^2^ The metabolites with VIP next to them were determined to be very important features by the PSL-DA. ^3^ These metabolites had concentrations lower than 0.01 for the sample with the lowest concentration. ^4^ These metabolites are shown in multiple pathways where a correlation was shown by Pattern Hunter.

**Table 5:** Fold change of statistically significant metabolites in their corresponding metabolic pathways for the stationary phase of DABCOMD challenged mutant versus DABCOMD challenged wild type.

Mut D S versus WT D S					
Indicated Pathway	Metabolite	Fold Change ^1,2^	Indicated Pathway	Metabolite	Fold Change
Citric Acid Cycle	NAD+ ^3^	+13.5	Alanine Metabolism	Aspartate ^4,^^5^	+1.5 (VIP)
	Isocitrate ^5^	−1.3 (VIP)		Uracil ^4^	−3.0 (VIP)
	Succinate	−1.9 (VIP)	Nucleotide	Adenine	−1.4 (VIP)
Pyruvate Metabolism	Acetate	+1.3 (VIP)	Metabolism	Uracil ^4^	−3.0 (VIP)
	Formate ^5^	+1.3 (VIP)	Aminoacyl-tRNA Biosynthesis	Alanine ^4,^^5^	−1.2 (VIP)
Peptidoglycan Synthesis	Alanine ^4,5^	−1.2 (VIP)		Aspartate ^4,^^5^	+1.5 (VIP)
	Cystathionine	+1.7		Glutamine	−2.1 (VIP)
	Glutamine ^4,5^	−2.1 (VIP)		Glycine ^4^	+1.5 (VIP)
	Glycine ^4,5^	+1.5 (VIP)		Leucine ^5^	+1.4 (VIP)
	N-Acetylglucosamine ^3^	+19.4		Phenylalanine	+1.5 (VIP)
Methionine Metabolism	Aspartate ^4,^^5^	+1.5 (VIP)		Valine	+1.7 (VIP)
	Cysteine	−1.6			

^1^ A positive fold change is indicative of a higher concentration in the mutant. ^2^ The metabolites with VIP next to them were determined to be very important features by the PSL-DA. ^3^ These metabolites had concentrations lower than 0.01 for the sample with the lowest concentration. ^4^ These metabolites are shown in multiple pathways where a correlation was shown by Pattern Hunter. ^5^ These metabolites do not have significant *p*-values, but they are VIPs.

**Table 6:** Fold change of statistically significant metabolites in their corresponding metabolic pathways for the mid log phase of DABCOMD challenged wild type versus unchallenged wild type.

WT D ML versus WT ML		
Indicated Pathway	Metabolite	Fold Change ^1,2^
Citric Acid Cycle	Isocitrate	−1.6
Pyruvate Metabolism	Acetate	−1.2 (VIP)
Aminoacyl-tRNA Biosynthesis	Alanine ^3^	+1.3 (VIP)
	Glutamate ^3^	+1.2 (VIP)
	Glutamine ^3^	−3.5 (VIP)
	Valine	+1.2
Peptidoglycan Synthesis	Alanine ^3^	+1.3 (VIP)
	Cystathionine	−1.6
	Glutamate ^3^	+1.2 (VIP)
	Glutamine ^3^	−3.5 (VIP)
	Lactate	+2.8

^1^ A positive fold change is indicative of a higher concentration in the mutant. ^2^ The metabolites with VIP next to them were determined to be very important features by the PSL-DA. ^3^ These metabolites are shown in multiple pathways where a correlation was shown by Pattern Hunter.

**Table 7:** Fold change of statistically significant metabolites in their corresponding metabolic pathways for the stationary phase of DABCOMD challenged wild type versus unchallenged wild type.

WT D S versus WT S					
Indicated Pathway	Metabolite	Fold Change ^1,2^	Indicated Pathway	Metabolite	Fold Change
Citric Acid Cycle	Succinate	+3.0 (VIP)	Peptidoglycan Synthesis	Alanine ^4^	+1.6 (VIP)
Pyruvate Metabolism	Acetate	−1.9 (VIP)		Glutamate ^4^	+1.6 (VIP)
Aminoacyl-tRNA Biosynthesis	Alanine ^4^	+1.6 (VIP)		Glutamine ^4^	−1.2
	Glutamate ^4^	+1.6 (VIP)	Glycine Metabolism	Betaine	+1.4 (VIP)
	Glutamine ^4^	−1.2			
	Histidine ^3^	+9.3	Nucleotide Metabolism	Uridine	−1.6
	Leucine	+1.9 (VIP)			
	Proline	−1.4 (VIP)			
	Valine	+1.9			

^1^ A positive fold change is indicative of a higher concentration in the mutant. ^2^ The metabolites with VIP next to them were determined to be very important features by the PSL-DA. ^3^ These metabolites had concentrations lower than 0.01 for the sample with the lowest concentration. ^4^ These metabolites are shown in multiple pathways where a correlation was shown by Pattern Hunter.

**Table 8:** Fold change of statistically significant metabolites in their corresponding metabolic pathways for the mid log phase of DABCOMD challenged mutant versus unchallenged mutant.

Mut D ML versus Mut ML					
Indicated Pathway	Metabolite	Fold Change ^1,2^	Indicated Pathway	Metabolite	Fold Change
Citric Acid Cycle	Acetoacetate	+2.2	Alanine Metabolism	Uracil ^4^	−4.7 (VIP)
	Isocitrate	+2.2			
	NAD+ ^3^	+3.0	Nucleotide Metabolism	Adenine	−1.5
	Succinate	+1.3		Uracil ^4^	−4.7 (VIP)
Pyruvate Metabolism	Acetate	+1.3 (VIP)	Aminoacyl-tRNA Biosynthesis	Alanine ^4^	−1.4 (VIP)
	Formate	+12.1 (VIP)		Aspartate ^4^	+2.1
	Pyruvate	+1.9		Glutamate ^4^	+1.6 (VIP)
Peptidoglycan Synthesis	Alanine ^4^	−1.4 (VIP)		Glutamine ^4^	+1.8 (VIP)
	Cystathionine	+4.7		Glycine^4^	+1.2
	Glutamate ^4^	+1.6 (VIP)		Leucine	−2.7
	Glutamine ^4^	+1.8 (VIP)		Phenylalanine	−2.0
	Glycine ^4^	+1.2		Proline	−1.5 (VIP)
Methionine Metabolism	Aspartate ^4^	+2.1		Tyrosine	−1.8
	Betaine ^4^	−4.0 (VIP)			
	Cysteine	+2.2			
	Homocysteine	+5.4			
Glycine Metabolism	Betaine ^4^	−4.0 (VIP)			
	

^1^ A positive fold change is indicative of a higher concentration in the mutant. ^2^ The metabolites with VIP next to them were determined to be very important features by the PSL-DA. ^3^ These metabolites had concentrations lower than 0.01 for the sample with the lowest concentration. ^4^ These metabolites are shown in multiple pathways where a correlation was shown by Pattern Hunter.

**Table 9:** Fold change of statistically significant metabolites in their corresponding metabolic pathways for the stationary phase of DABCOMD-challenged mutant versus unchallenged mutant.

Mut D S versus Mut S					
Indicated Pathway	Metabolite	Fold Change ^1,2^	Indicated Pathway	Metabolite	Fold Change
Citric Acid Cycle	Succinate	+2.3 (VIP)	Nucleotide Metabolism	Adenine	−2.7 (VIP)
Pyruvate Metabolism	Acetate	+2.6 (VIP)		Adenosine	−4.4
	Formate	+10.1 (VIP)		Uracil	−8.6 (VIP)
Peptidoglycan Synthesis	Glutamate ^3,4^	+1.3 (VIP)	Aminoacyl-tRNA Biosynthesis	AMP	−2.1
	Glutamine ^3^	−1.7		Glutamate ^3,4^	+1.3 (VIP)
	Glycine ^3^	+2.9 (VIP)		Glutamine ^3^	−1.7
	Lactate	+1.9		Glycine ^3^	+2.9 (VIP)
	Lysine	−2.0		Histidine	−3.4
	N-Acetylglucosamine	−2.5		Isoleucine	−2.7 (VIP)
Glycine Metabolism	Betaine	+2.8 (VIP)		Leucine	+1.5
				Phenylalanine	−2.2 (VIP)
				Tyrosine	−1.5

^1^ A positive fold change is indicative of a higher concentration in the mutant. ^2^ The metabolites with VIP next to them were determined to be very important features by the PSL-DA. ^3^ These metabolites are shown in multiple pathways where a correlation was shown by Pattern Hunter. ^4^ These metabolites do not have significant *p*-values, but they are VIPs.

**Table 10:** Fold change of statistically significant metabolites in their corresponding metabolic pathways for the mid log phase of DABCOMD challenged wild type versus unchallenged mutant.

WT D ML versus Mut ML					
Indicated Pathway	Metabolite	Fold Change ^1,2^	Indicated Pathway	Metabolite	Fold Change
Citric Acid Cycle	Acetoacetate	+3.5	Alanine Metabolism	Uracil ^4^	+1.6 (VIP)
	Fumarate ^3^	−5.0			
	Isocitrate	−2.8	Nucleotide Metabolism	Adenine	+1.4
	NAD+ ^3^	−12.4		Adenosine	−2.0
	Succinate	−1.7 (VIP)		Uracil ^4^	+1.6 (VIP)
Pyruvate Metabolism	Pyruvate	−1.5	Aminoacyl-tRNA Biosynthesis	Alanine ^4^	+1.5 (VIP)
				AMP ^3^	−9.1
Peptidoglycan Synthesis	Alanine ^4^	+1.5 (VIP)		Glutamine ^4^	−2.3
	Cystathionine	−2.3		Glycine ^4^	+2.0
	Glutamine ^4^	−2.3		Histidine ^3^	−11.3
	Glycine ^4^	+2.0		Isoleucine	+5.7 (VIP)
	Lactate	+1.4		Leucine	+4.3 (VIP)
	N-Acetylglucosamine ^3^	−61.5		Phenylalanine	+2.0 (VIP)
Methionine Metabolism	Betaine ^4^	−3.6 (VIP)		Tyrosine	+1.9 (VIP)
	Methionine	+2.9 (VIP)		Valine	+4.6 (VIP)
Glycine Metabolism	Betaine ^4^	−3.6 (VIP)			
			

^1^ A positive fold change is indicative of a higher concentration in the mutant. ^2^ The metabolites with VIP next to them were determined to be very important features by the PSL-DA. ^3^ These metabolites had concentrations lower than 0.01 for the sample with the lowest concentration. ^4^ These metabolites are shown in multiple pathways where a correlation was shown by Pattern Hunter.

**Table 11:** Fold change of statistically significant metabolites in their corresponding metabolic pathways for the stationary phase of DABCOMD challenged wild type versus unchallenged mutant.

WT D S versus Mut S					
Indicated Pathway	Metabolite	Fold Change ^1,2^	Indicated Pathway	Metabolite	Fold Change
Citric Acid Cycle	Acetoacetate	−1.6	Glycine Metabolism	Betaine ^4^	+3.2 (VIP)
	Fumarate ^3^	−8.9			
	NAD+ ^3^	−11.1	Alanine Metabolism	Uracil ^4^	−2.9 (VIP)
	Succinate	+4.4 (VIP)			
Pyruvate Metabolism	Acetate	+2.1 (VIP)	Nucleotide Metabolism	Adenine	−1.9 (VIP)
	Formate	+7.9 (VIP)		Adenosine	−4.3
	Pyruvate	+1.5		Uracil^4^	−2.9 (VIP)
Peptidoglycan Synthesis	Cystathionine	−1.7	Aminoacyl-tRNA Biosynthesis	AMP	−2.3
	Glutamate ^4,5^	+1.4 (VIP)		Glutamate ^4,5^	+1.4 (VIP)
	Glycine ^4^	+2.0 (VIP)		Glycine ^4^	+2.0 (VIP)
	Lactate	+1.5		Histidine	−2.3
	Lysine	−2.4 (VIP)		Phenylalanine	−3.2 (VIP)
	N-Acetylglucosamine ^3^	−48.7		Tyrosine	−1.7
Methionine Metabolism	Betaine ^4^	+3.2 (VIP)		Valine	−1.7 (VIP)
	Homocysteine	+1.5			

^1^ A positive fold change is indicative of a higher concentration in the mutant. ^2^ The metabolites with VIP next to them were determined to be very important features by the PSL-DA. ^3^ These metabolites had concentrations lower than 0.01 for the sample with the lowest concentration. ^4^ These metabolites are shown in multiple pathways. ^5^ These metabolites do not have significant *p*-values, but they are VIPs.

## Data Availability

The data presented in this study are openly available in NIH Common Fund’s National Metabolomics Data Repository (NMDR) website, the Metabolomics Workbench, where it has been assigned Study ID ST001966 [66].

## Supplementary Materials

The following are available online at https://www.mdpi.com/article/10.3390/ijms222413606/s1.
