# Supplementary material for: NMR Hydrophilic Metabolomic Analysis of Bacterial Resistance Pathways Using Multivalent Antimicrobials with Challenged and Unchallenged Wild Type and Mutated Gram-Positive Bacteria
Source: Int J Mol Sci. 2021 Dec 19;22(24):13606. doi: 10.3390/ijms222413606 (PMC8715671; doi:10.3390/ijms222413606)
Supplement: Supplementary file 1 [file ijms-22-13606-s001.zip › ijms-1485480-supplementary.pdf]

Supplementary Material

NMR Hydrophilic Metabolomic Analysis of Bacterial Resistance Pathways using Multivalent Antimicrobials with Challenged and Unchallenged Wild Type and Mutated Gram-Positive Bacteria

Michelle L. Aries and Mary J. Cloninger

Department of Chemistry and Biochemistry, Montana State University, Bozeman, MT, 59717 USA, mcloninger@montana.edu

Table of Contents

Detailed Methods Description	Pages S2-S4
Growth Curve	Page S4
Sample NMR Spectra	Page S4
Table S1 Significant Metabolites Identified	Page S5
Hierarchical Clustering	Pages S6
Ortho PLS-DA	Pages S7
PCA	Pages S8
3D sPLS-DA	Pages S9
Volcano Plots	Pages S10-S11
VIP Plots	Pages S12-S14
Challenged Mutant versus Unchallenged Wild Type Tables S2 and S3	Pages S15-S16
Pattern Hunter Metabolite Correlations	Pages S17-S33
Pathway Analysis Plots	Pages S34-S40
Box Plots	Page S40-S62
References	Page S63

Procedures

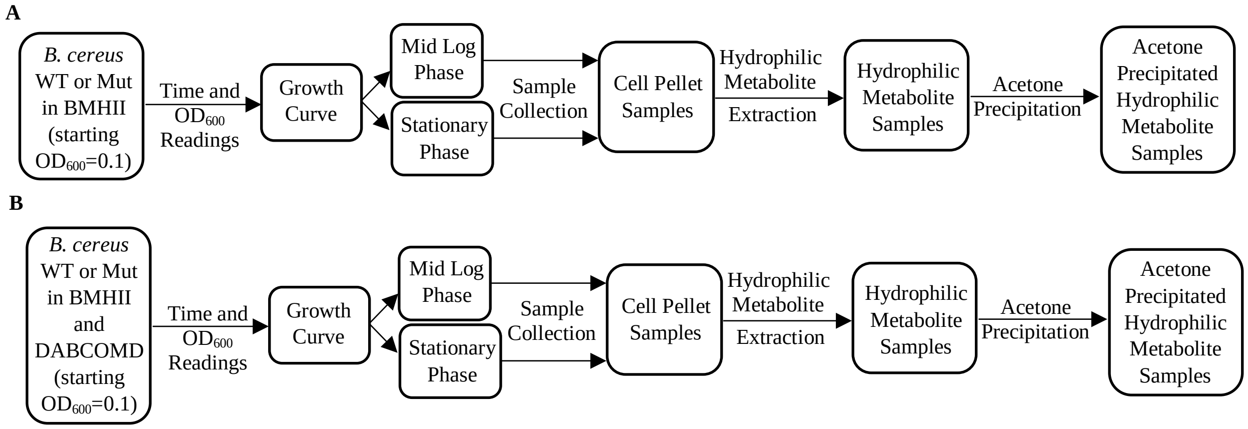

Figure S1. Procedure overview. Overview of the start of the cultures to the hydrophilic metabolite samples ready to be pelleted and frozen at -80 ºC then put into NMR buffer. (**a**) unchallenged sample procedures (**b**) challenged sample procedures.

Preparation and Sterilization of Media

Growth media (1 L) was prepared by adding 22 g of Brodo Mueller Hinton II Media (BMHII) and filling to 1 L with Millipore water in a 2 L Erlenmeyer flask, which was capped with aluminum foil with a strip of autoclave tape. The media was heated and stirred until homogeneous with a stir bar on a hot plate for 10-15 minutes, then autoclaved. Sterile methods were used whenever the aluminum foil lid was removed.

Sterile Technique

The autoclave was used on all supplies and solutions that needed to be sterilized and could withstand the autoclave. The door in the hood was never raised more than a foot. The hood, all equipment and supplies, my gloves and lab coat sleeves were sprayed down with 70% ethanol. No lids were removed until after the ethanol dried, only when necessary and for as short of a time as possible. A Bunsen burner was lit, and the rim of each flask was passed through the flame before and after any pouring occurred.

Overnight Culture Preparation

In a 250 mL Erlenmeyer flask, 80 mL of the already prepared and sterilized BMHII media was poured using sterile technique. Frozen stock and mutated *B. cereus* samples were kept on ice while separate sterile pipette tips were used to scrape the frozen bacterial stock. The pipette tips with frozen bacterial stock were ejected into separate media filled 250 mL Erlenmeyer flasks. These flasks were put in an incubator at 37 ºC with 250 revolutions per minute (rpms) overnight.

Starting the Culture

The overnight cultures were cloudy and used to start the sample cultures. Sterile technique was used to add 500 mL of sterile BMHII media to each 1 L flask, and overnight culture was added until the optical density at 600 nm (OD_{600}) reached 0.1. After each flask's OD_{600} was brought up 0.1, they were placed back into the 37 ºC incubator at 250 rpms.

OD_{600} Readings
A blank was created by filling a capped cuvette with sterile media. Sterile media (0.20 mL) and 0.80 mL of sterile Millipore water was used to create a 1:5 dilution blank. The OD_{600} readings were conducted on a SpectraMax plus ® SoftMax Pro 5 ® Molecular Devices Spectrometer set to 600 nm wavelength. Before any cuvette was placed into the spectrometer, it was wiped down with a Kimwipe. A blank spectrum was acquired prior to running a sample cuvette filled with 1 mL of culture. The OD_{600} was recorded and plotted versus time (min) to generate the sample growth plots.

Culture Growth
All cultures were kept in the 37 ºC incubator at 250 rpms, exception during sample collection. To generate the growth plot and track individual sample progress, the OD_{600} was taken every half of an hour for the first few cultures and every hour for the last few cultures of every sample type grown. When the sample's OD_{600} was going to be greater than 1, a 1:5 dilution of all samples was enacted in the OD cuvettes to keep them in the linear phase. Sample collection was conducted in the mid log (half of the ending OD_{600}) and stationary phases. After the stationary phase samples were collected, the cultures were left overnight and the OD_{600} was taken again, to ensure that the OD_{600} did not increase and that the cultures were in stationary phase the night before.

Sample Collection
Samples were collected in the mid log phase (half the ending OD_{600}) and the stationary phase. Two 150 mL aliquots per culture were combined to form one sample. Screw-cap conical centrifuge tubes were used to centrifuge the samples at 2,500 rpms for 12 minutes. The supernatant was discarded, and the cell pellets were washed with 15 mL of cold sterile 1x PBS. The samples were transferred to glass centrifuge tubes. A 1:10 dilution was used to obtain OD_{600} readings of for each sample. They were centrifuged at 2,500 rpms for 12 minutes for the wild type samples, and for 24 minutes for the mutant samples, since it took longer for the mutant samples to centrifuge down.

Dry Mass Measurements
At an OD_{600} of 1, 25 mL of sample culture was collected. They were centrifuged at 2500 rpms for 25 minutes and the supernatant was discarded. The cell pellets were rinsed with 5 mL of sterile 1x PBS and vortexed, then centrifuged at 2500 rpms for 25 minutes. The supernatant was discarded, and the cell pellet was scraped onto a sterile and pre-massed watch glass. The watch glasses were placed in the oven at 100 ºC for 10 hours. The masses of the dried samples were obtained. (Aries and Cloninger 2020; Wang)

Colony Forming Units (CFUs)
An YTP Agar solution was created from 4 g yeast extract, 8 g tryptone, 8 g glucose, 6.4 g agar and 400 mL Millipore water. After the solution was boiled, it was poured into petri dishes. A glass test tube labeled A contained 9.9 mL of sterile PBS and 100 µL of culture and was gently mixed with a pipette tip. A glass test tube labeled B contained 9 mL of sterile PBS and 1 mL from test tube A and was gently mixed with a pipette tip. A glass test tube labeled C contained 9 mL of sterile PBS and 1 mL from test tube B and was gently mixed with a pipette tip. A glass

test tube labeled D contained 9 mL of sterile PBS and 1 mL from test tube C and was gently mixed with a pipette tip. A 100 µL aliquot was taken from test tube D and pipetted onto an agar plate and a bacterial spreader was used. This was repeated nine times per sample type. The agar plates were incubated at 37 °C for 24 hours. Each plate was divided into sections and the CFUs were counted. The number of CFUs per milliliter was calculated. (Aries and Cloninger 2020; Wang; Ammons et al. 2014 and references therein)

Data

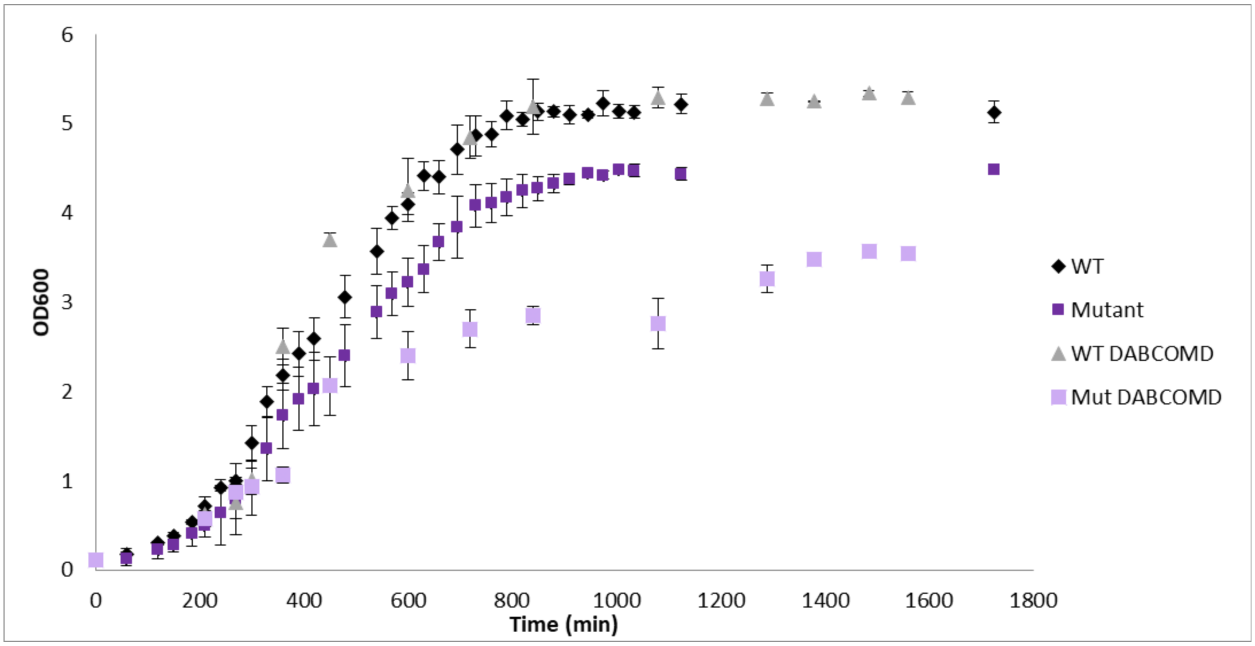

Figure S2. Growth Plot of Wild Type, DABCOMD Mutated *B. cereus*, DABCOMD challenged Wild Type and DABCOMD challenged DABCOMD Mutated *B. cereus*. The challenged groups are labeled with DABCOMD after the sample type.

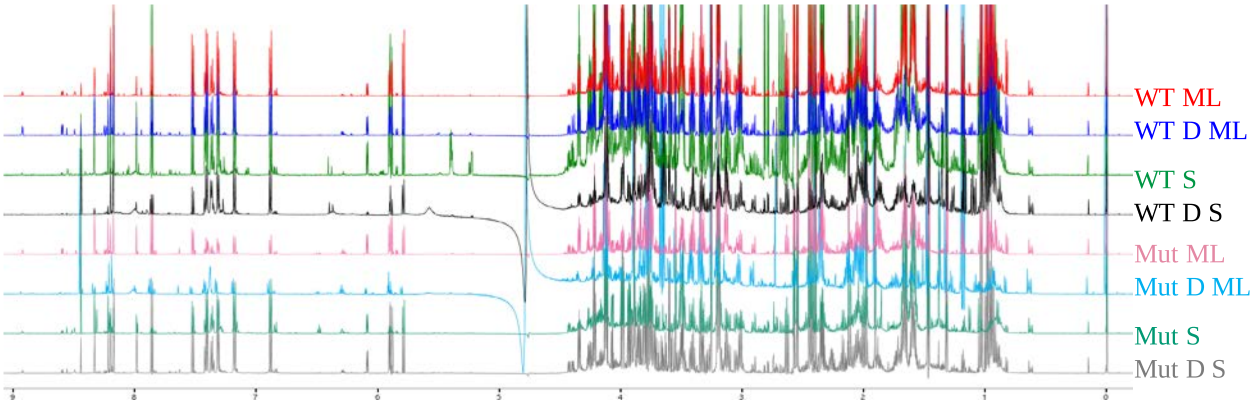

Figure S3. Spectra for each of the 8 sample types.

Table S1. All Metabolites Identified using the Chenomx NMR Suite 8.4 Software Profiling Program and Metabolite Library

All Identified Metabolites	CAS #
Acetate	71-50-1
Acetoacetate	541-50-4
Adenine	73-24-5
Adenosine	58-61-7
Alanine	56-41-7
AMP	61-19-8
Aspartate	56-84-8
Betaine	107-43-7
Cystathionine	56-88-2
Cysteine	54-90-4
Formate	141-53-7
Fucose	2438-80-4
Fumarate	142-42-7
Glutamate	56-86-0
Glutamine	56-85-9
Glycine	56-40-6
Histidine	71-00-1
Homocysteine	6027-13-0
Isocitrate	320-77-4
Isoleucine	73-32-5
Lactate	50-21-5
Leucine	61-90-5
Lysine	56-87-1
Methionine	59-51-8
N-Acetylglucosamine	7512-17-6
NAD+	53-84-9
Phenylalanine	63-91-2
Proline	609-36-9
Pyroglutamate	28874-51-3
Pyruvate	127-17-3
Serine	302-84-1
Succinate	110-15-6
Tyrosine	60-18-4
UDP-glucose	133-89-1
Uracil	66-22-8
Uridine	58-96-8
Valine	72-18-4

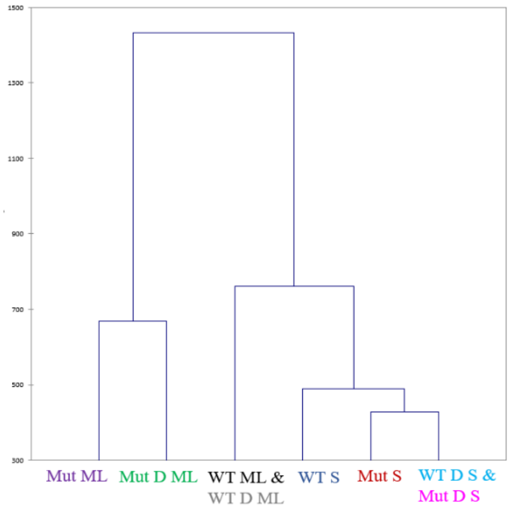

(a)

Key

Wild Type Mid Log	(WT ML)	WT_1
Wild Type Stationary	(WT S)	WT_2
Wild Type DABCOMD Mid Log	(WT D ML)	WT_3
Wild Type DABCOMD Stationary	(WT D S)	WT_4
Mutant Mid Log	(Mut ML)	Mut_1
Mutant Stationary	(Mut S)	Mut_2
Mutant DABCOMD Mid Log	(Mut D ML)	Mut_3
Mutant DABCOMD Stationary	(Mut D S)	Mut_4

(b)

Results by class:

Class	1	2	3	4	5	6
	WT_1_1	Mut_1_10	Mut_3_57	WT_2_22	WT_4_65	Mut_2_111
	WT_1_2	Mut_1_11	Mut_3_58	WT_2_23	WT_4_67	Mut_2_112
	WT_1_4	Mut_1_99	Mut_3_60	WT_2_25	WT_4_68	Mut_2_113
	WT_1_5	Mut_1_100	Mut_3_61	WT_2_27	WT_4_69	Mut_2_114
	WT_1_6	Mut_1_101	Mut_3_63	WT_2_173	WT_4_70	Mut_2_115
	WT_3_9	Mut_1_102	Mut_3_64		Mut_4_81	
	WT_3_42	Mut_1_175			Mut_4_82	
	WT_3_43				Mut_4_83	
	WT_3_44				Mut_4_84	
	WT_3_45				Mut_4_85	
	WT_3_46				Mut_4_86	

(c)

Mid Log Phase

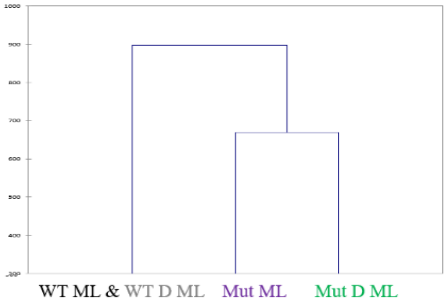

(d)

Stationary Phase

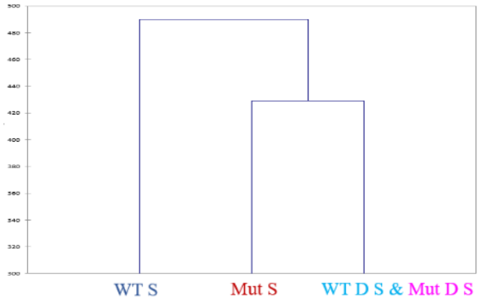

(e)

Unchallenged

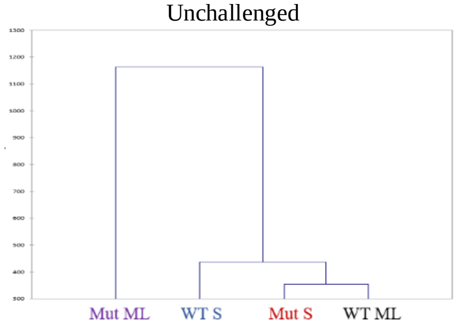

(f)

Challenged

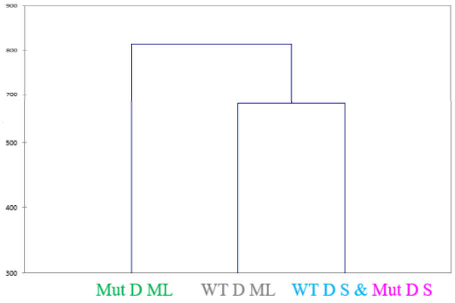

(g)

Figure S4. (a) Hierarachical Clustering of all sample types showing that WT ML and WT D ML cluster together, WT D S and Mut D S cluster together and all other sample types separate. (b) Sample abbreviation key. (c) Resulting classification of all input data showing the clustering of each individual sample. (d) Hierarchical Clustering of mid log phase samples showing that WT ML and WT D ML cluster together, and all other sample types separate. (e) Hierarchical Clustering of stationary phase samples showing that WT D S and Mut D S cluster together, and all other sample types separate. (f) Hierarchical Clustering of unchallenged sample types showing complete separation of sample types. (g) Hierarchical Clustering of DABCOMD challenged samples showing that the stationary phases cluster together, while the mid log phases separate.

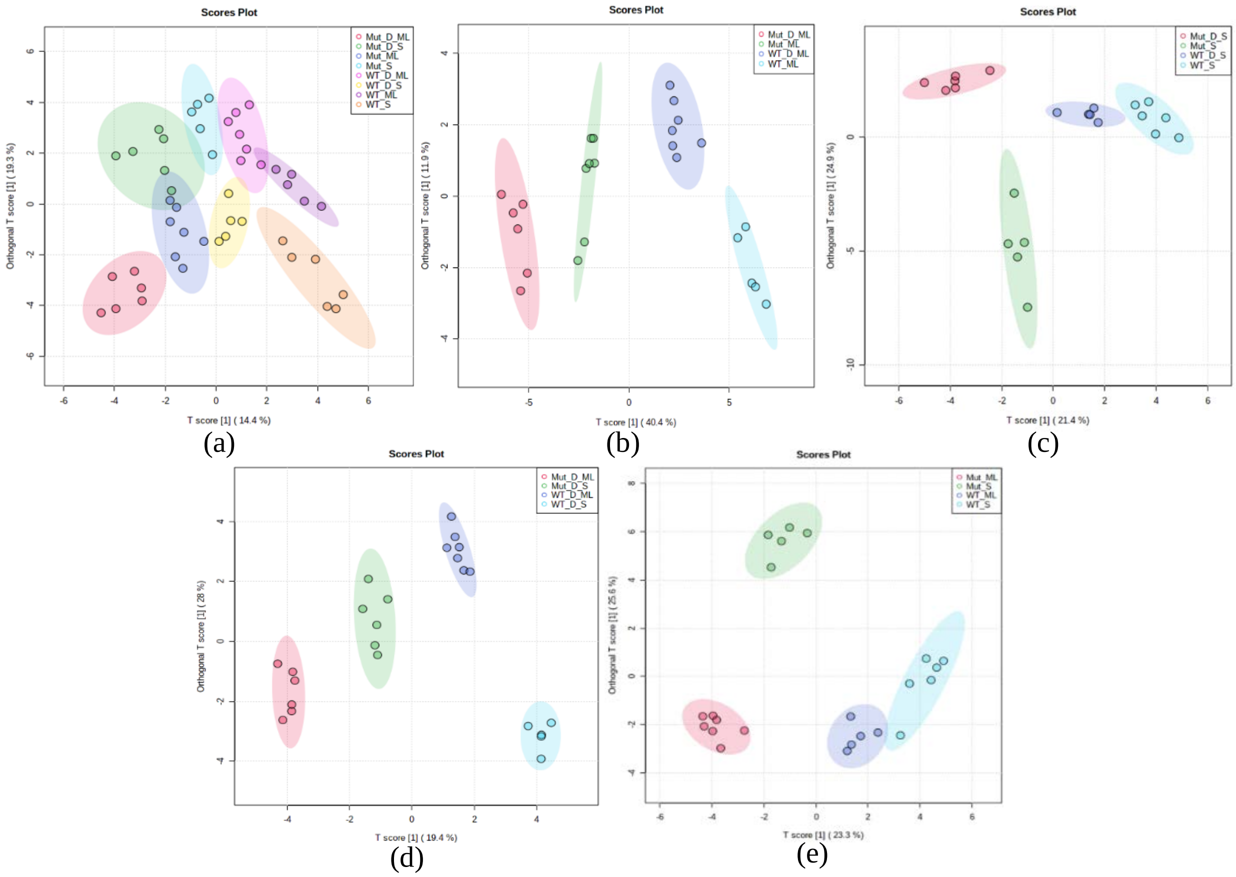

Figure S5. 2D ortho PLS-DA (**a**) Contains all sample types: demonstrates the greatest degree of separation with all sample types together, with only an overlap of the oval of WT ML with the oval of WT D ML, and a overlap of the oval of Mut D S with the ovals of WT S and Mut ML. (**b-e**) demonstrates complete separation of all sample types: (**b**) mid log phase samples. (**c**) stationary phase samples. (**d**) DABCOMD challenged samples. (**e**) unchallenged samples.

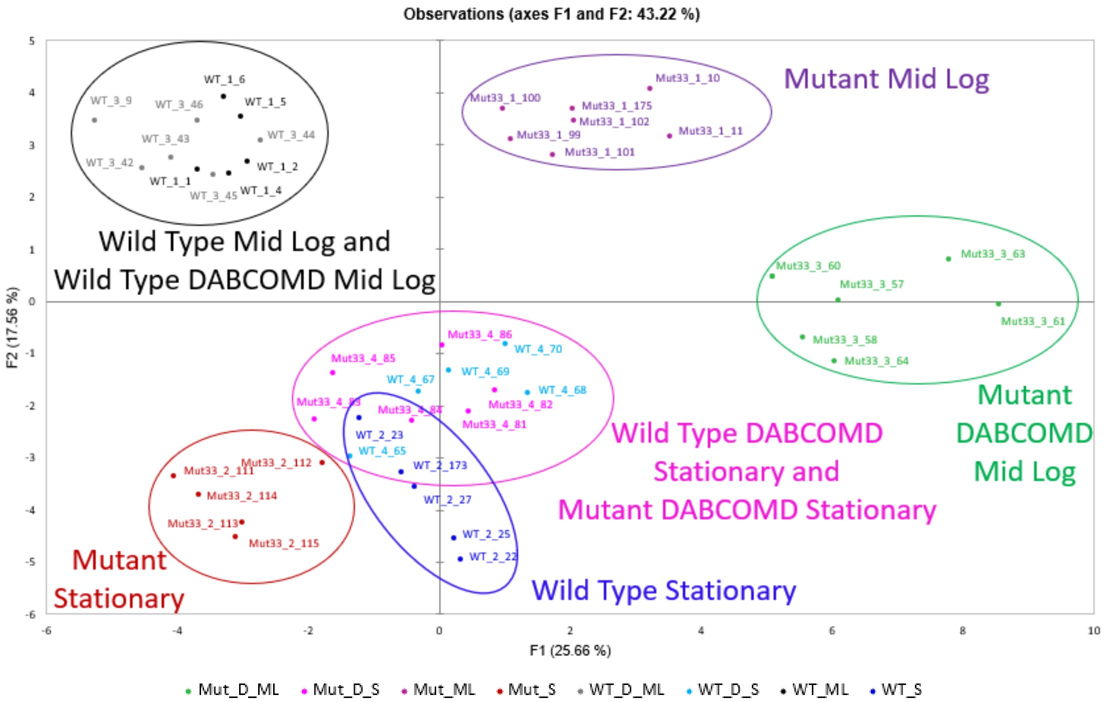

Figure S6. PCA plot showing the distribution of all sample types with sample labels corresponding to those listed in Figure S4c.

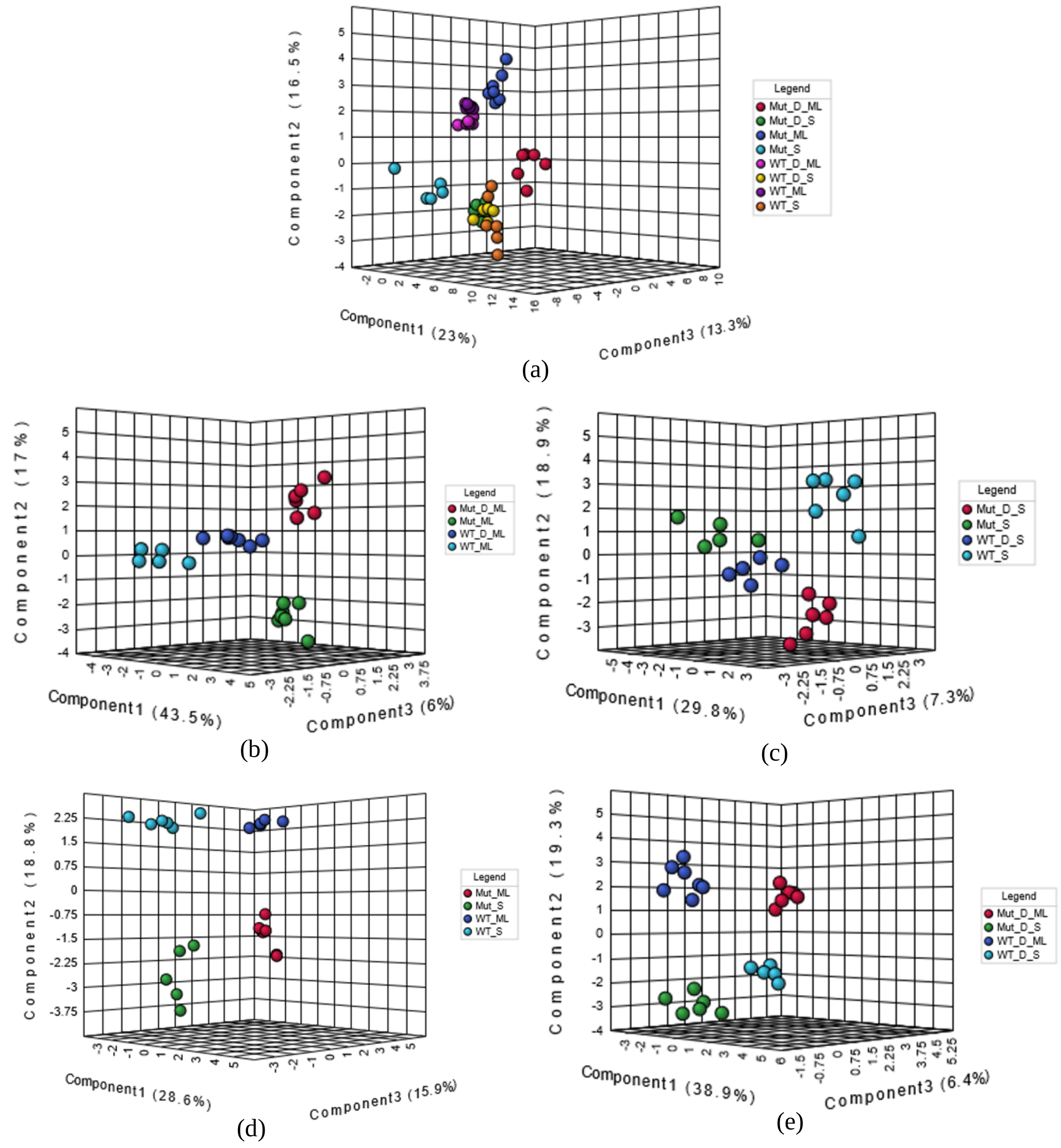

Figure S7. 3D sPLS-DA **a** Contains all sample types: showing a slight overlap of WT ML with WT D ML, Mut ML separated, Mut D ML separated, Mut S separated, while WT S, WT D S and Mut D S clustered. **b** Contains only mid log samples: showing WT ML clustering near WT D ML and complete separatation of Mut ML and Mut D ML. **c** Contains only stationary phase samples: showing WT D S clustering between Mut D S and Mut S, WT S being the most distinct group. **d** Contains only unchallenged samples: showing a complete separation of sample types **e** Contains only DABCOMD challenged samples: showing complete separation of all sample types.

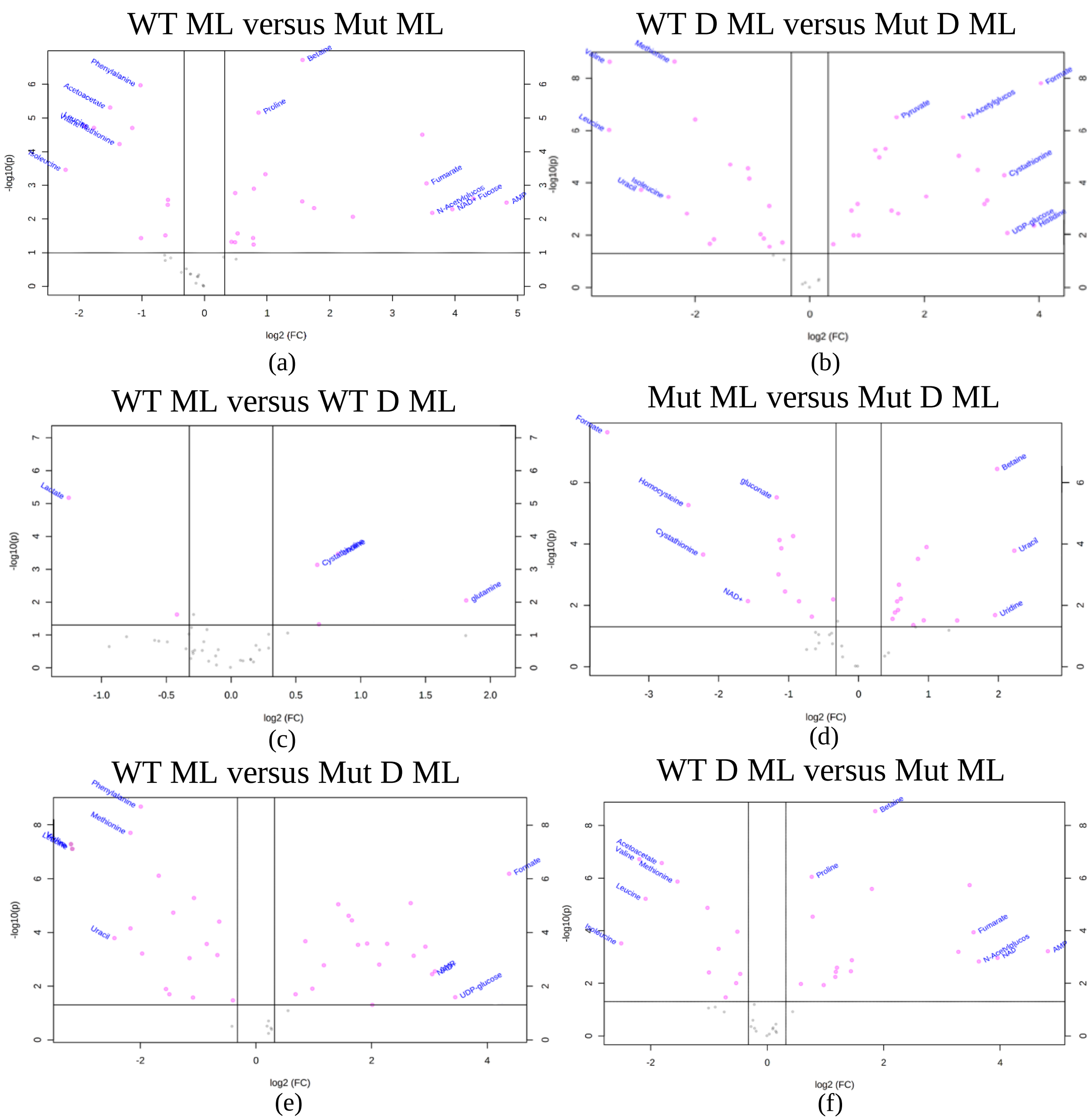

Figure S8. Volcano Plot showing significant p-values and fold changes for metabolites in the mid log phase paired up **a** WT and Mut **b** WT D and Mut D **c** WT and WT D **d** Mut and Mut D **e** WT and Mut D **f** WT D and Mut.

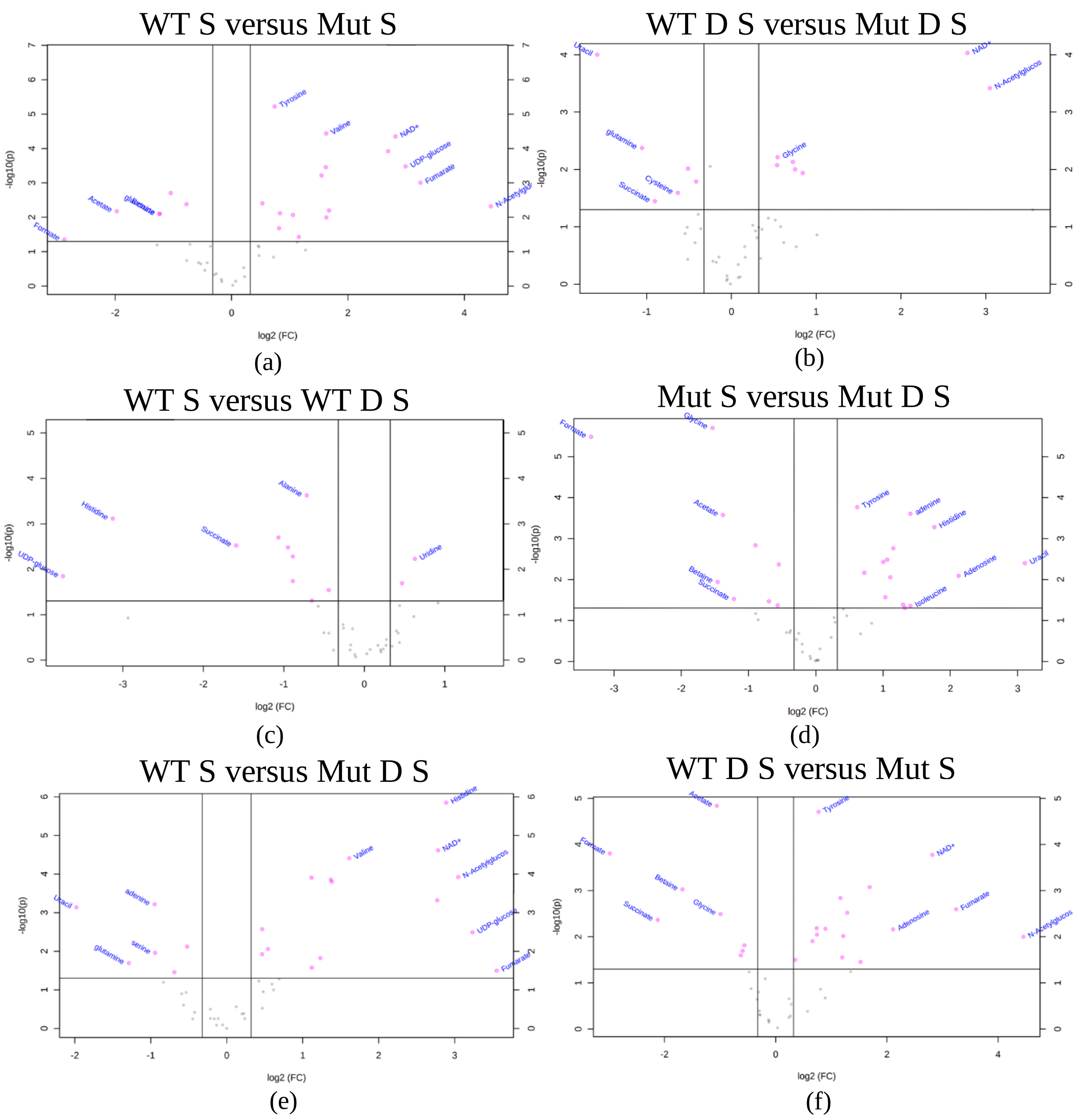

Figure S9. Volcano Plot showing significant p-values and fold changes for metabolites in the stationary phase paired up **a** WT and Mut **b** WT D and Mut D **c** WT and WT D **d** Mut and Mut D **e** WT and Mut D **f** WT D and Mut.

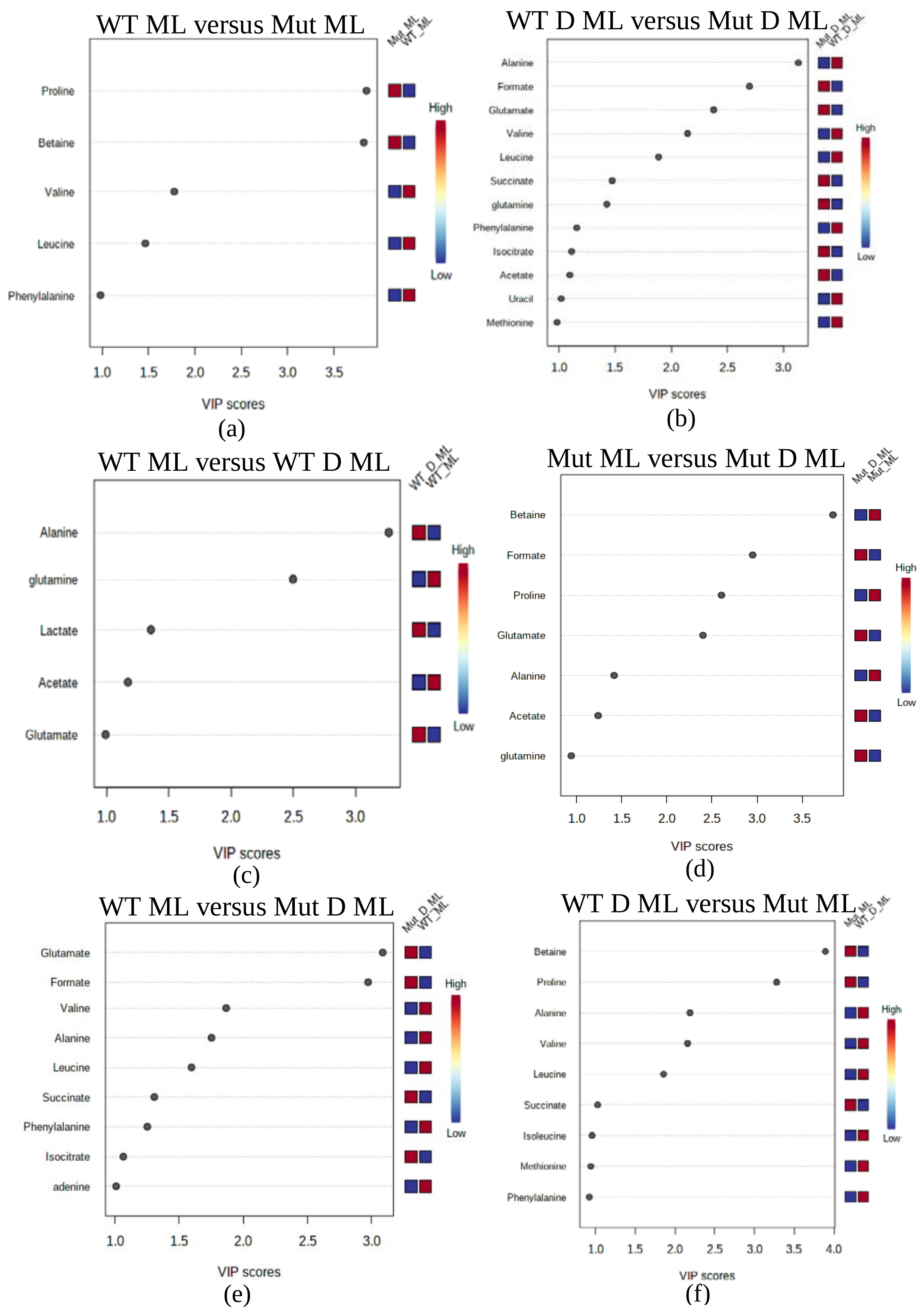

Figure S10. Very Important Features (VIP) scores obtained from the sPLS-DA which shows important metabolites in paired mid log phase samples **a** WT and Mut **b** WT D and Mut D **c** WT and WT D **d** Mut and Mut D **e** WT and Mut D **f** WT D and Mut.

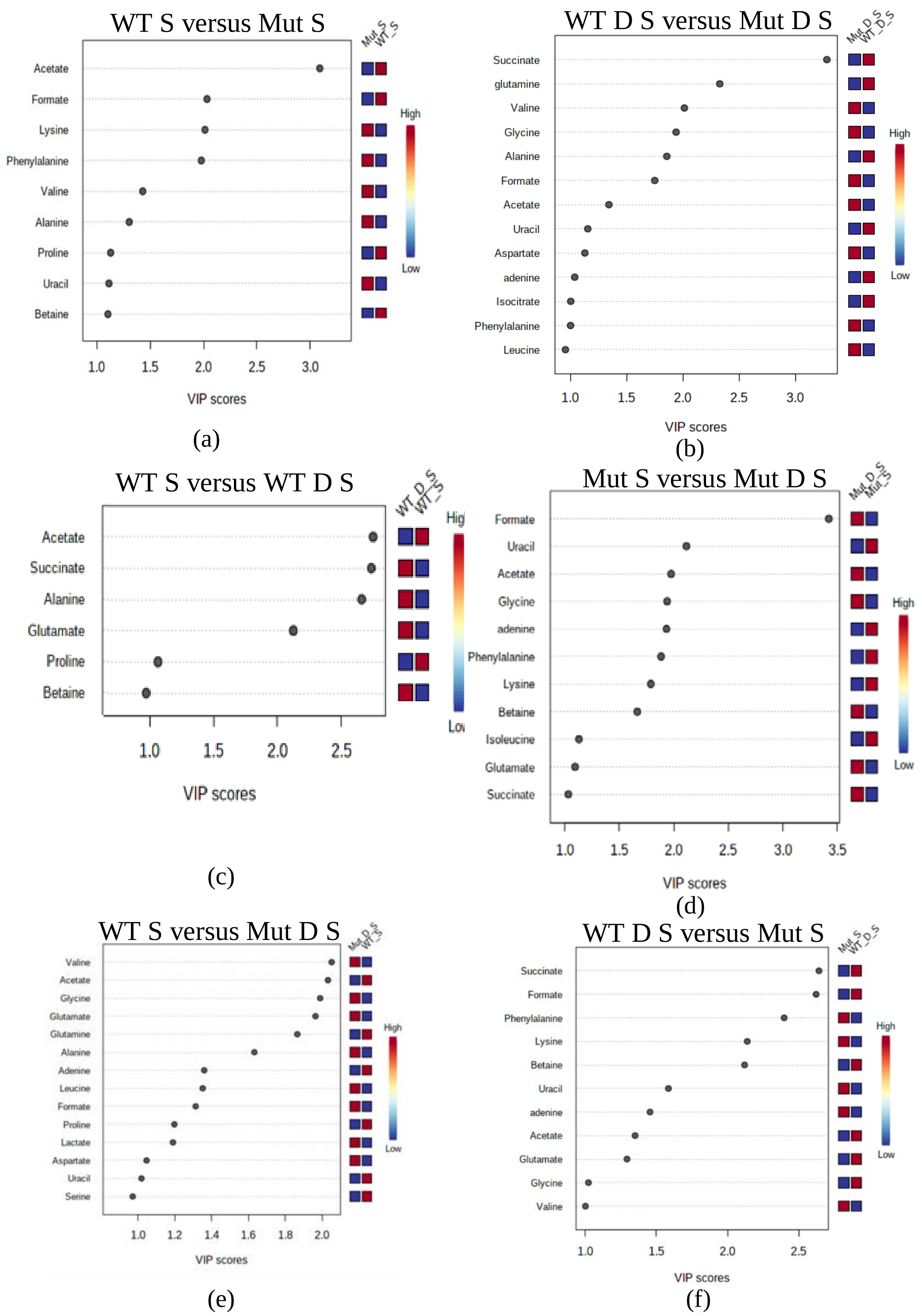

Figure S11. Very Important Features (VIP) scores obtained from the sPLS-DA which shows important metabolites in paired stationary phase samples **a** WT and Mut **b** WT D and Mut D **c** WT and WT D **d** Mut and Mut D **e** WT and Mut D **f** WT D and Mut.

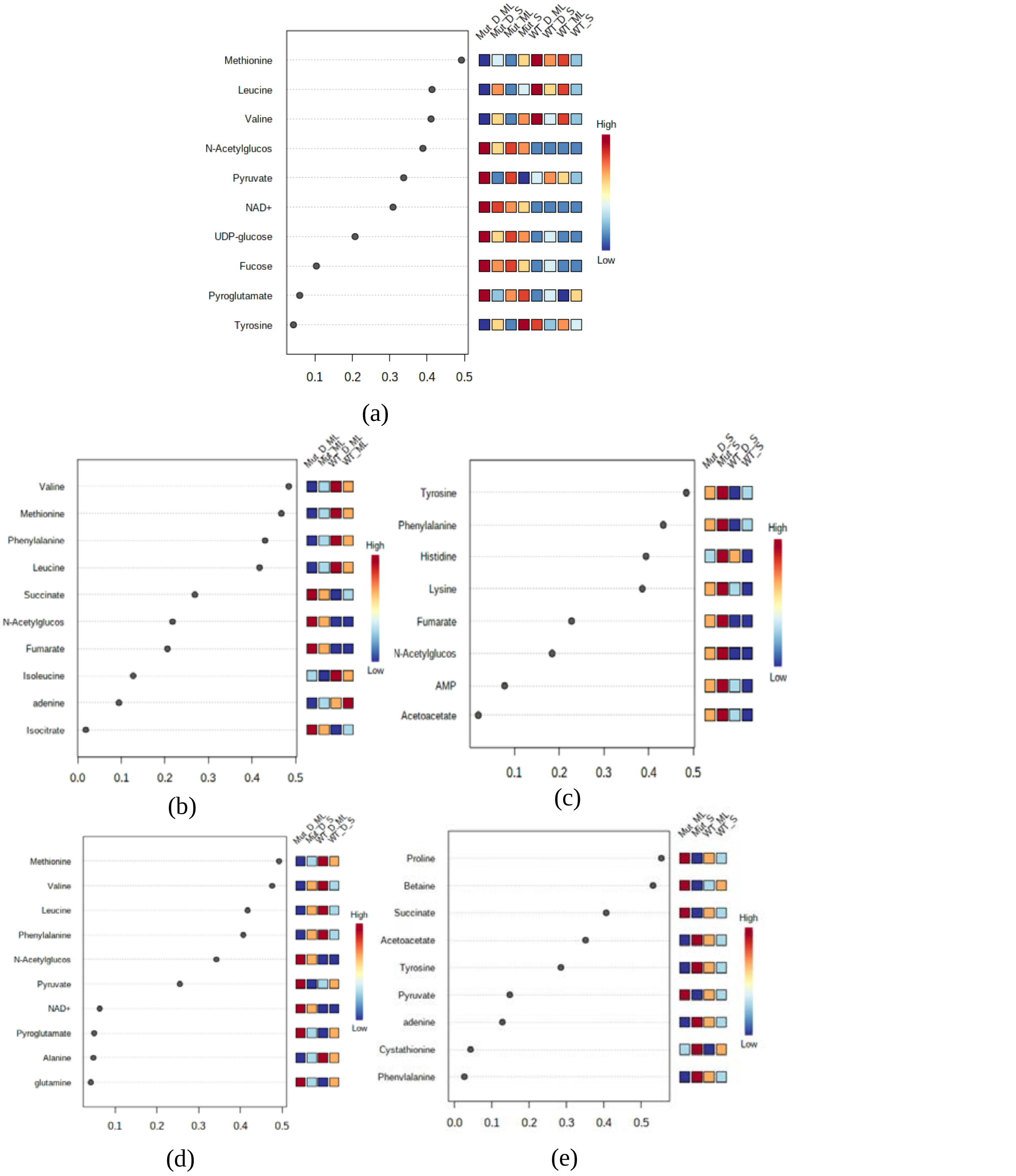

Figure S12. Very Important Features (VIP) scores obtained from the sPLS-DA which shows important metabolites in **a** all samples types **b** mid log samples **c** stationary phase samples **d** DABCOMD challenged samples **e** unchallenged samples

Table S2. Fold change of statistically significant metabolites in their corresponding metabolic pathways for the mid log phase of DABCOMD challenged mutant versus unchallenged wild type.

Mut D ML versus WT ML					
**Indicated Pathway**	**Metabolite**	**Fold Change[1,2]**	**Indicated Pathway**	**Metabolite**	**Fold Change**
Citric Acid Cycle	Acetoacetate	- 1.3	Alanine Metabolism	Uracil[4]	- 5.5 (VIP)
	Fumarate[3]	+ 5.8			
	Isocitrate	+ 3.8	Nucleotide Metabolism	Adenine	- 2.2 (VIP)
	NAD+[3]	+ 37.0 (VIP)		Uracil•	- 5.5 (VIP)
	Succinate	+ 1.8 (VIP)		Uridine	- 3.9
Pyruvate Metabolism	Formate	+ 20.8 (VIP)	Aminoacyl-tRNA Biosynthesis	Alanine[4]	- 1.6 (VIP)
	Pyruvate	+ 2.7		AMP[3]	+ 6.8
Peptidoglycan Synthesis	Alanine[4]	- 1.6 (VIP)		Aspartate[4]	+ 1.6
	Cystathionine	+ 6.6		Glutamate[4]	+ 2.0 (VIP)
	Glutamate[4]	+ 2.0 (VIP)		Glycine[4]	- 1.8
	Glycine[4]	- 1.8		Histidine	+ 4.8
	Lactate	+ 2.2		Isoleucine	- 4.5
	N-Acetylglucosamine[3]	+ 94.2		Leucine	- 9.0 (VIP)
Methionine Metabolism	Aspartate[4]	+ 1.6		Phenylalanine	- 4.0 (VIP)
	Cysteine	+ 3.2		Tyrosine	- 2.7
	Homocysteine	+ 5.1		Valine	- 9.2 (VIP)
	Methionine	- 4.5			

[1]A positive fold change is indicative of a higher concentration in the mutant. [2]The metabolites with VIP next to them were determined to be very important features by the PSL-DA. [3]These metabolites had concentrations lower than 0.01 for the sample with the lowest concentration. [4]These metabolites are shown in multiple pathways where a correlation was shown by Pattern Hunter.

Table S3. Fold change of statistically significant metabolites in their corresponding metabolic pathways for the stationary phase of DABCOMD challenged mutant versus unchallenged wild type.

Mut D S versus WT S					
**Indicated Pathway**	**Metabolite**	**Fold Change[1,2]**	**Indicated Pathway**	**Metabolite**	**Fold Change**
Citric Acid Cycle	Fumarate[3]	+ 3.6	Alanine Metabolism	Uracil[4]	- 3.9 (VIP)
	NAD+[3]	+ 13.5			
	Isocitrate[5]	- 1.2	Nucleotide Metabolism	Adenine	- 1.9 (VIP)
	Succinate[5]	+ 1.6		Uracil[4]	- 3.9 (VIP)
Pyruvate Metabolism	Formate	+ 1.4 (VIP)	Aminoacyl-tRNA Biosynthesis	Alanine[4]	+ 1.4 (VIP)
				Aspartate[4]	+ 2.2 (VIP)
Peptidoglycan Synthesis	Alanine[4]	+ 1.4 (VIP)		Glutamate[4,5]	+ 1.5 (VIP)
	Glutamate[4,5]	+ 1.5 (VIP)		Glycine[4]	+ 2.2
	Glycine[4]	+ 2.2 (VIP)		Histidine[3]	+ 6.4
	Lactate	+ 2.6 (VIP)		Leucine	+ 2.6 (VIP)
	Lysine	+ 1.5		Phenylalanine	+ 1.4 (VIP)
	N-Acetylglucosamine[3]	+ 19.4		Proline	- 1.4 (VIP)
Methionine Metabolism	Aspartate[4]	+ 2.2 (VIP)		Valine	+ 3.1 (VIP)
	Serine	- 1.9 (VIP)			

[1]A positive fold change is indicative of a higher concentration in the mutant. [2]The metabolites with VIP next to them were determined to be very important features by the PSL-DA. [3]These metabolites had concentrations lower than 0.01 for the sample with the lowest concentration. [4]These metabolites are shown in multiple pathways where a correlation was shown by Pattern Hunter. [5]These metabolites do not have significant p-values, but they are VIPs.

Challenged Mutant versus Unchallenged Wild Types Comparisons for Tables S2 and S3

When comparing challenged mutant mid log (Mut D ML) to unchallenged wild type (WT) ML, comparisons of Mut to WT and challenged to unchallenged are effectively being concurrently evaluated. This unsurprisingly leads to many observable differences in the concentrations of metabolites in energy production, aminoacyl-tRNA biosynthesis, peptidoglycan synthesis and related pathways. For example, the challenged mutant samples had higher concentrations of energy related metabolites, except for acetoacetate which was lower. It is particularly interesting that isocitrate was 3.8-fold higher and formate was 20.8-fold higher in the mutant samples. The challenged Mut samples had a 37-fold increase in NAD+ and a 6.8-fold increase in AMP when compared to the unchallenged WT. N-acetylglucosamine had a 94.2-fold increase in the challenged Mut samples when compared to the unchallenged WT samples. Aspartate correlated to cysteine, homocysteine, methionine, and cystathionine. Nucleotide metabolism components in the mid log phase had a decreased concentration in the challenged mutant samples, and multiple components of aminoacyl-tRNA biosynthesis changed. When comparing Mut D S to WT S, the same types of pathways changed as with the mid log phase, although the fold change differences were not as large. N-acetylglucosamine, lysine, lactate, glycine, glutamate (VIP) and alanine (VIP), all components of peptidoglycan synthesis, were in higher concentration in the challenged mutant stationary phase, in comparison to the unchallenged wild type stationary phase.

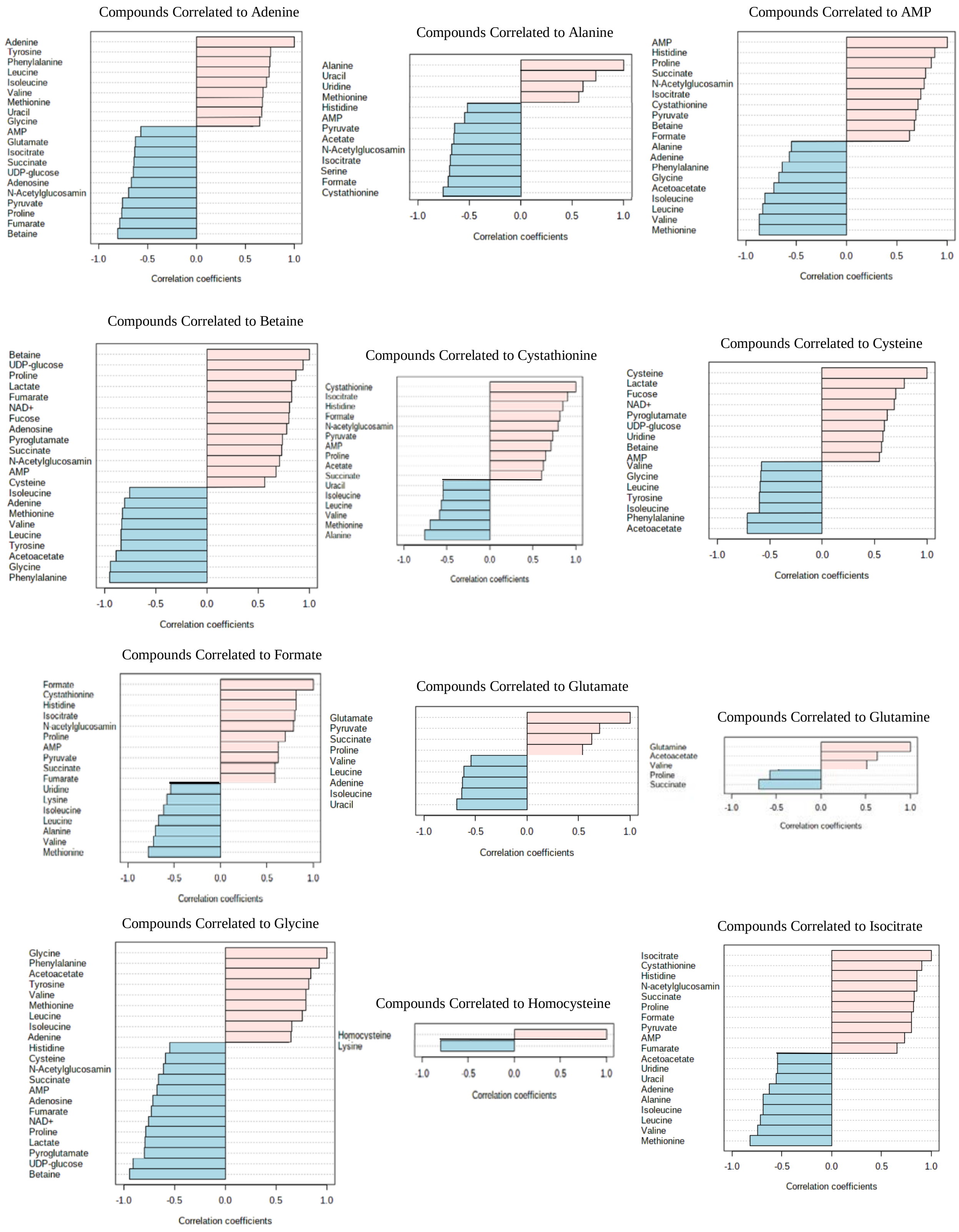
Compounds Correlated to Adenine
Adenine
Tyrosine
Phenylalanine
Leucine
Isoleucine
Valine
Methionine
Uracil
Glycine
AMP
Glutamate
Isocitrate
Succinate
UDP-glucose
Adenosine
N-Acetylglucosamin
Pyruvate
Proline
Fumarate
Betaine
-1.0 -0.5 0.0 0.5 1.0
Correlation coefficients
Compounds Correlated to Alanine
Alanine
Uracil
Uridine
Methionine
Histidine
AMP
Pyruvate
Acetate
N-Acetylglucosamin
Isocitrate
Serine
Formate
Cystathionine
-1.0 -0.5 0.0 0.5 1.0
Correlation coefficients
Compounds Correlated to AMP
AMP
Histidine
Proline
Succinate
N-Acetylglucosamin
Isocitrate
Cystathionine
Pyruvate
Betaine
Formate
Alanine
Adenine
Phenylalanine
Glycine
Acetoacetate
Isoleucine
Leucine
Valine
Methionine
-1.0 -0.5 0.0 0.5 1.0
Correlation coefficients
Compounds Correlated to Betaine
Betaine
UDP-glucose
Proline
Lactate
Fumarate
NAD+
Fucose
Adenosine
Pyroglutamate
Succinate
N-Acetylglucosamin
AMP
Cysteine
Isoleucine
Adenine
Methionine
Valine
Leucine
Tyrosine
Acetoacetate
Glycine
Phenylalanine
-1.0 -0.5 0.0 0.5 1.0
Correlation coefficients
Compounds Correlated to Cystathionine
Cystathionine
Isocitrate
Histidine
Formate
N-acetylglucosamin
Pyruvate
AMP
Proline
Acetate
Succinate
Uracil
Isoleucine
Leucine
Valine
Methionine
Alanine
-1.0 -0.5 0.0 0.5 1.0
Correlation coefficients
Compounds Correlated to Cysteine
Cysteine
Lactate
Fucose
NAD+
Pyroglutamate
UDP-glucose
Uridine
Betaine
AMP
Valine
Glycine
Leucine
Tyrosine
Isoleucine
Phenylalanine
Acetoacetate
-1.0 -0.5 0.0 0.5 1.0
Correlation coefficients
Compounds Correlated to Formate
Formate
Cystathionine
Histidine
Isocitrate
N-acetylglucosamin
Proline
AMP
Pyruvate
Succinate
Fumarate
Uridine
Lysine
Isoleucine
Leucine
Alanine
Valine
Methionine
-1.0 -0.5 0.0 0.5 1.0
Correlation coefficients
Compounds Correlated to Glutamate
Glutamate
Pyruvate
Succinate
Proline
Valine
Leucine
Adenine
Isoleucine
Uracil
-1.0 -0.5 0.0 0.5 1.0
Correlation coefficients
Compounds Correlated to Glutamine
Glutamine
Acetoacetate
Valine
Proline
Succinate
-1.0 -0.5 0.0 0.5 1.0
Correlation coefficients
Compounds Correlated to Glycine
Glycine
Phenylalanine
Acetoacetate
Tyrosine
Valine
Methionine
Leucine
Isoleucine
Adenine
Histidine
Cysteine
N-Acetylglucosamin
Succinate
AMP
Adenosine
Fumarate
NAD+
Proline
Lactate
Pyroglutamate
UDP-glucose
Betaine
-1.0 -0.5 0.0 0.5 1.0
Correlation coefficients
Compounds Correlated to Homocysteine
Homocysteine
Lysine
-1.0 -0.5 0.0 0.5 1.0
Correlation coefficients
Compounds Correlated to Isocitrate
Isocitrate
Cystathionine
Histidine
N-acetylglucosamin
Succinate
Proline
Formate
Pyruvate
AMP
Fumarate
Acetoacetate
Uridine
Uracil
Adenine
Alanine
Isoleucine
Leucine
Valine
Methionine
-1.0 -0.5 0.0 0.5 1.0
Correlation coefficients

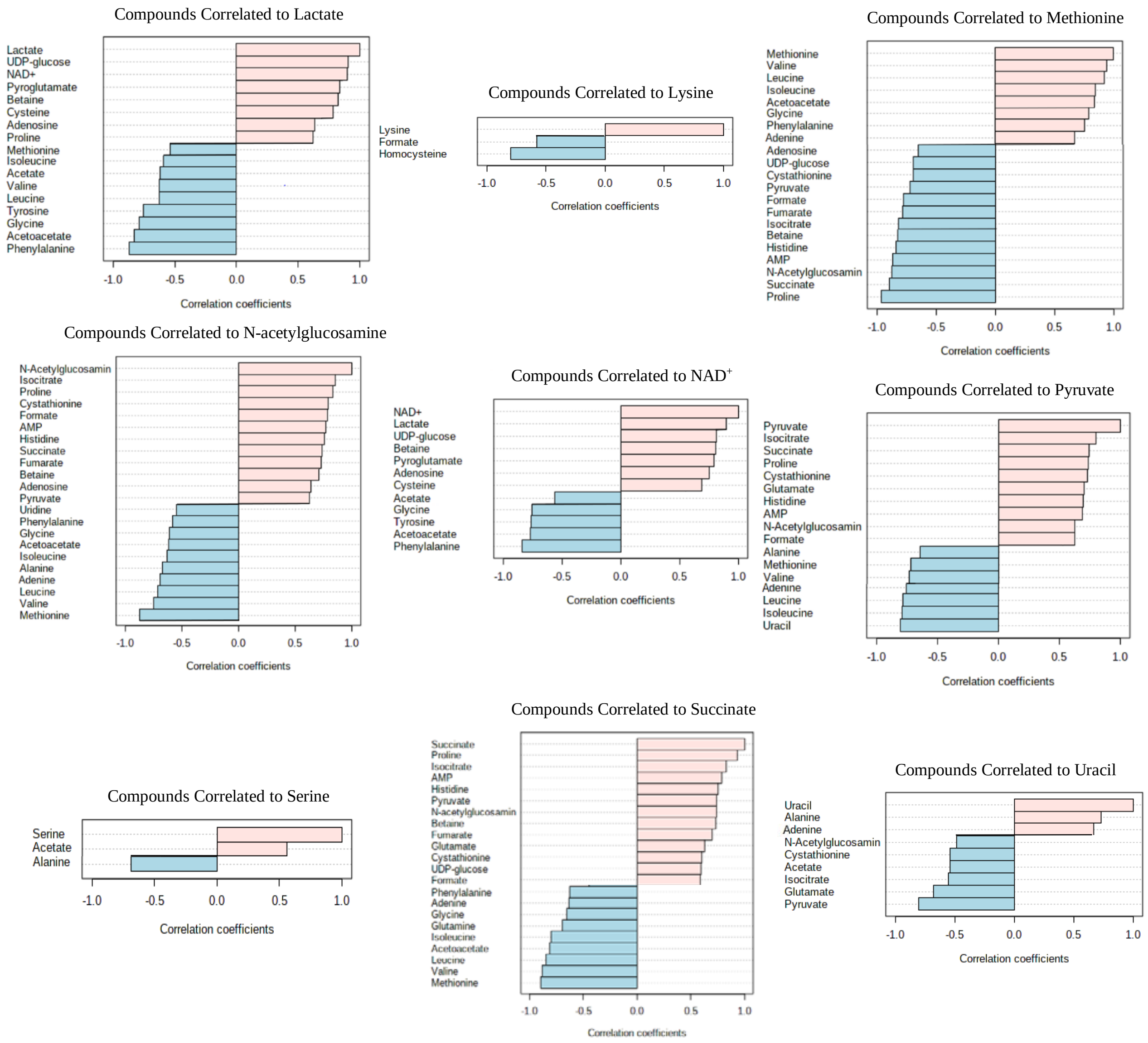

Figure S13. Pattern Hunter Correlations between Metabolites Observed in the Mid Log Phase Comparison of Unchallenged Mutant and Unchallenged Wild Type Samples.

Compounds Correlated to Adenine

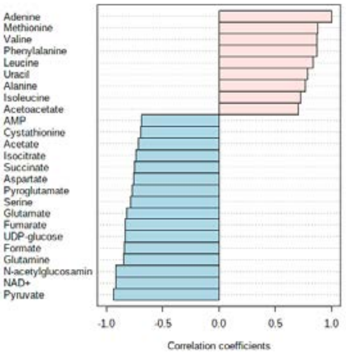

Compounds Correlated to Alanine

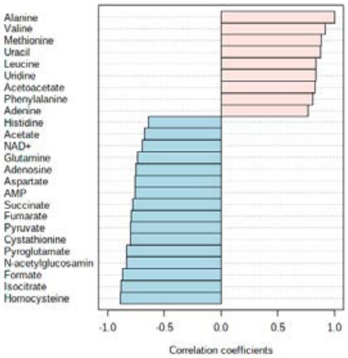

Compounds Correlated to AMP

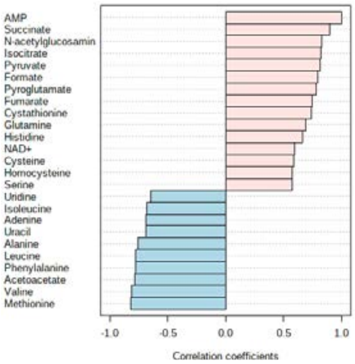

Compounds Correlated to Aspartate

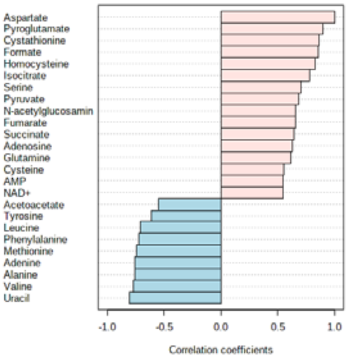

Compounds Correlated to Betaine

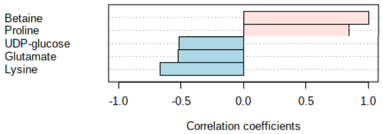

Compounds Correlated to Cystathionine

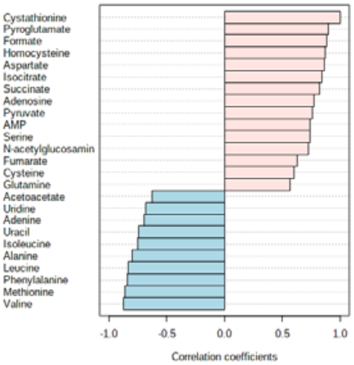

Compounds Correlated to Cysteine

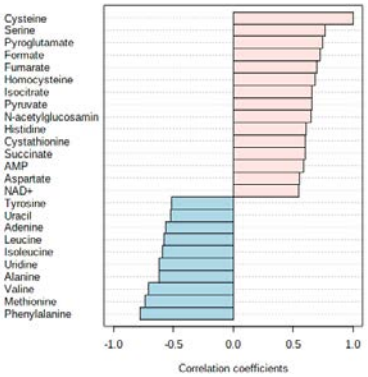

Compounds Correlated to Formate

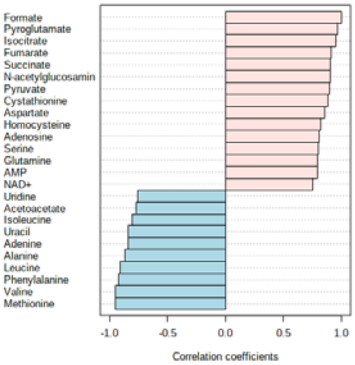

Compounds Correlated to Glutamate

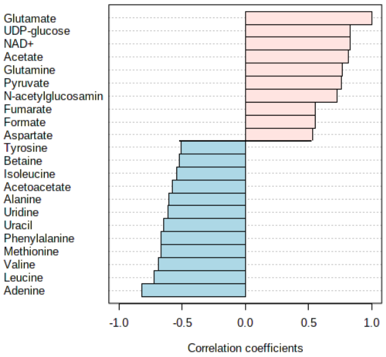

Compounds Correlated to Glutamine

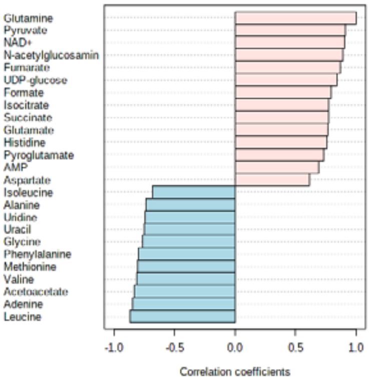

Compounds Correlated to Glycine

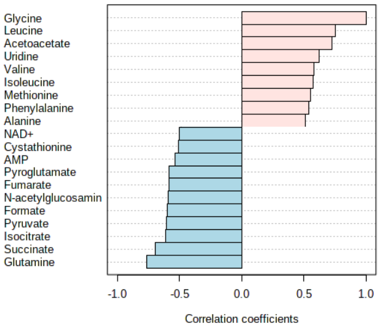

Compounds Correlated to Homocysteine

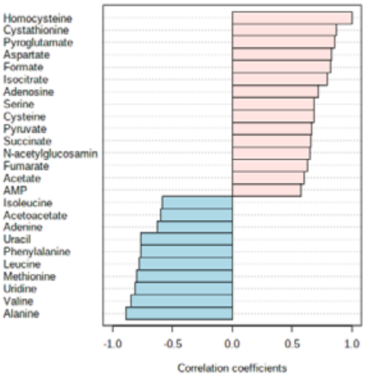

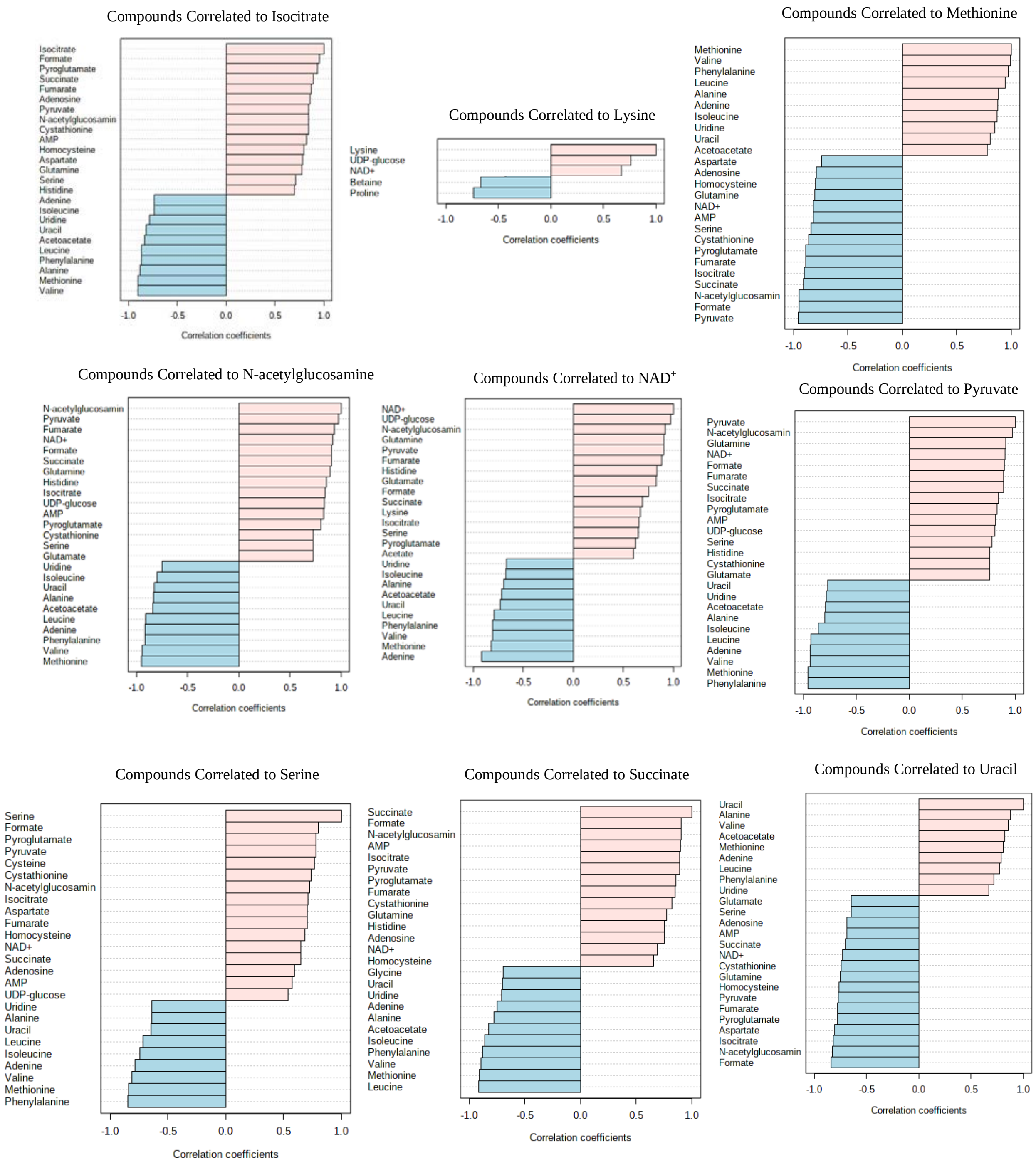

Figure S14. Pattern Hunter Correlations between Metabolites Observed in the Mid Log Phase Comparison of Challenged Mutant and Challenged Wild Type Samples.

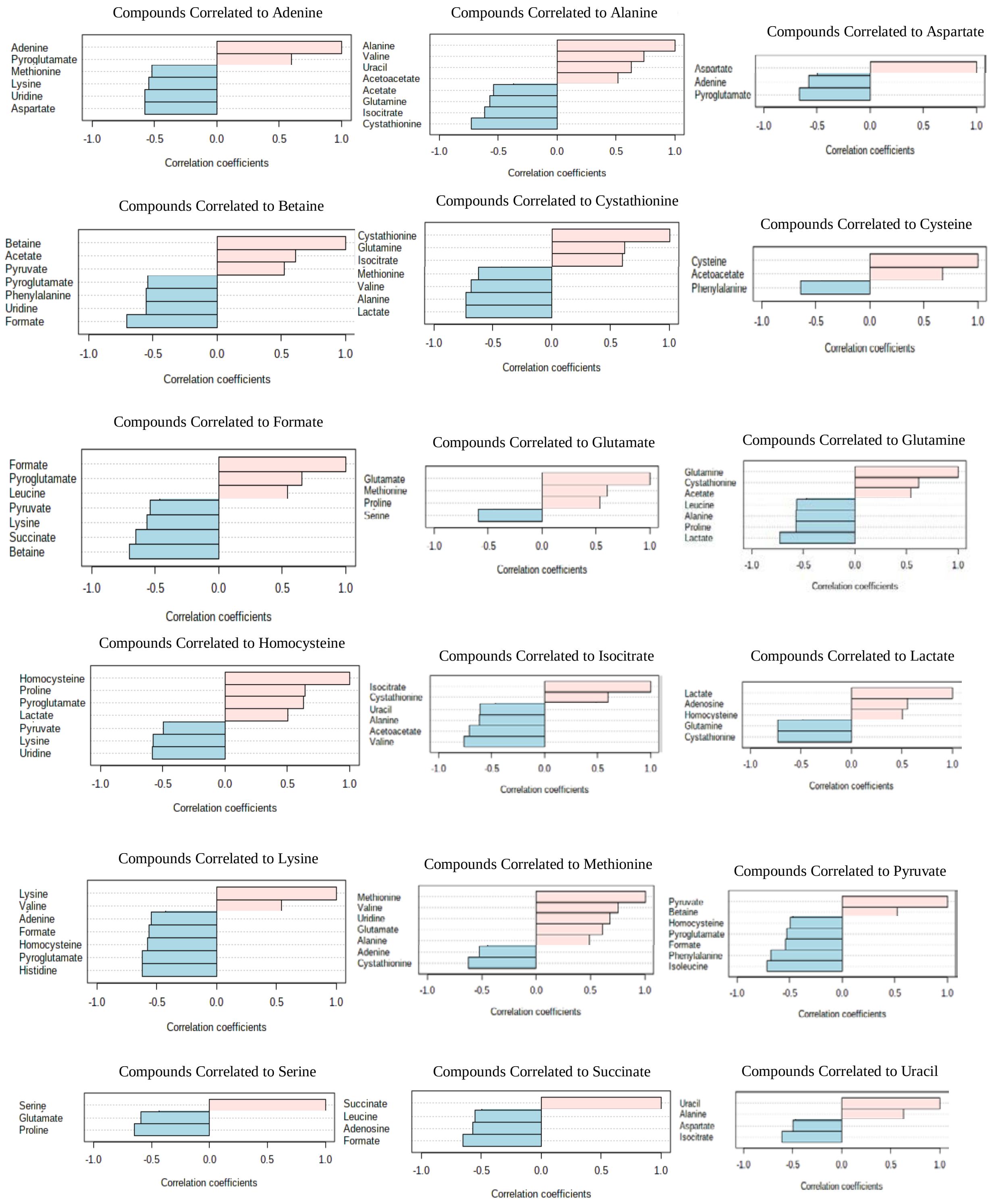

Figure S15. Pattern Hunter Correlations between Metabolites Observed in the Mid Log Phase Comparison of Challenged Wild Type and Unchallenged Wild Type Samples.

Compounds Correlated to Adenine

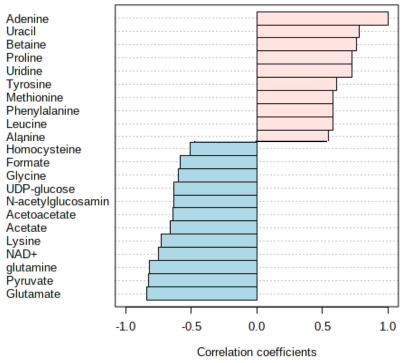

Compounds Correlated to Alanine

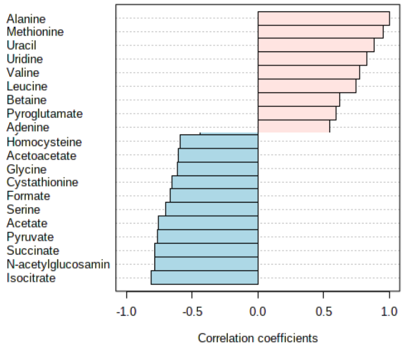

Compounds Correlated to AMP

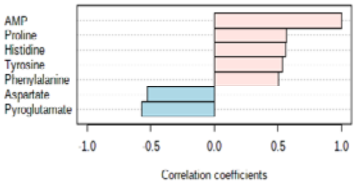

Compounds Correlated to Aspartate

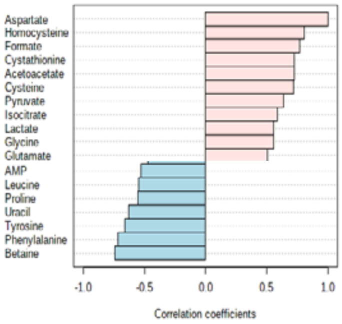

Compounds Correlated to Betaine

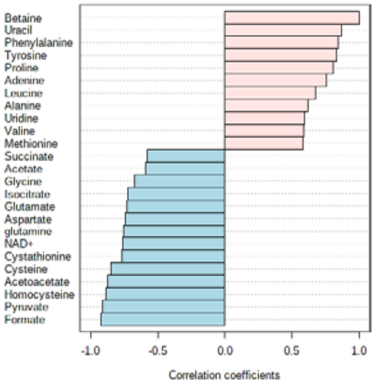

Compounds Correlated to Cystathionine

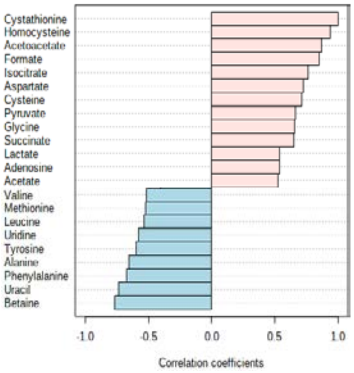

Compounds Correlated to Cysteine

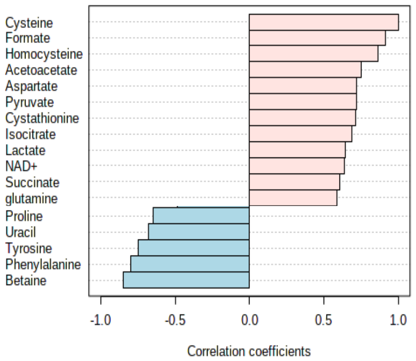

Compounds Correlated to Formate

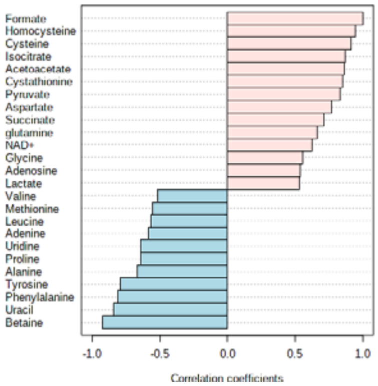

Compounds Correlated to Glutamate

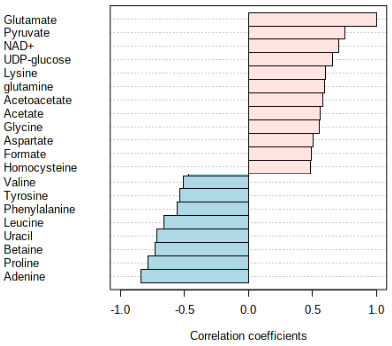

Compounds Correlated to Glutamine

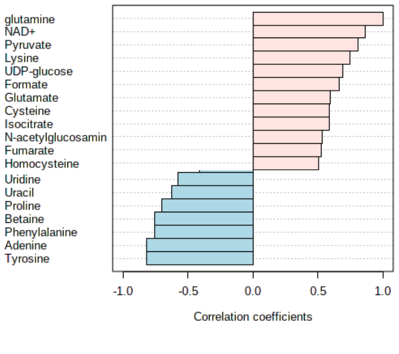

Compounds Correlated to Glycine

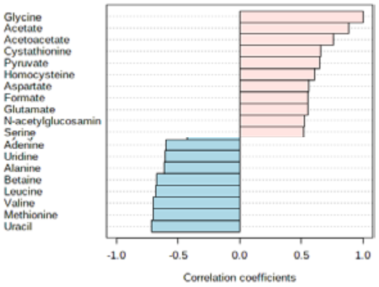

Compounds Correlated to Homocysteine

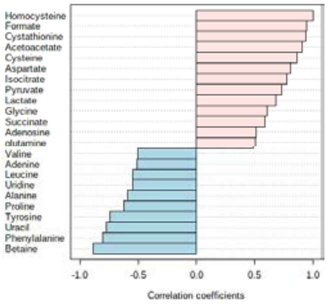

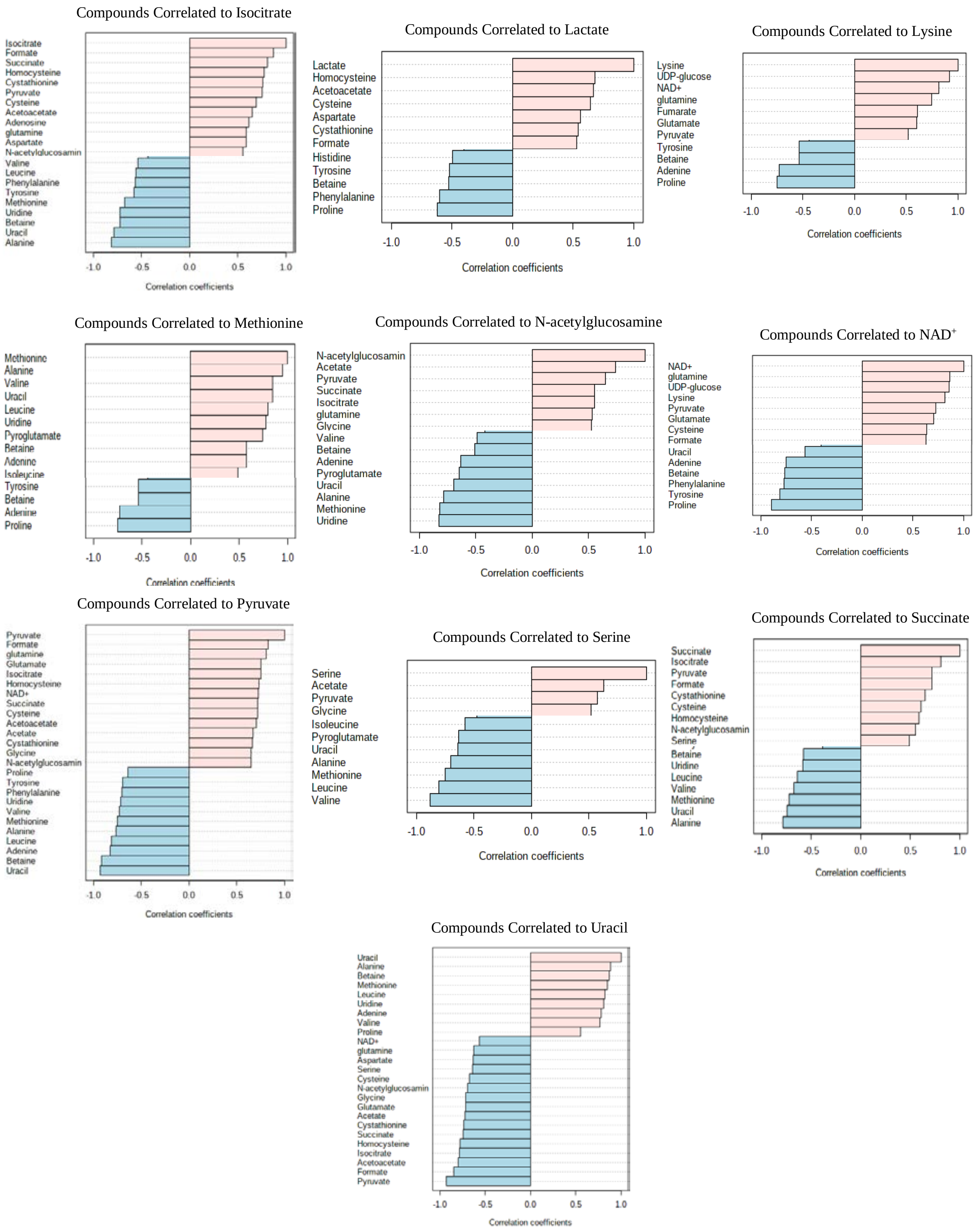

Figure S16. Pattern Hunter Correlations between Metabolites Observed in the Mid Log Phase Comparison of Challenged Mutant and Unchallenged Mutant Samples.

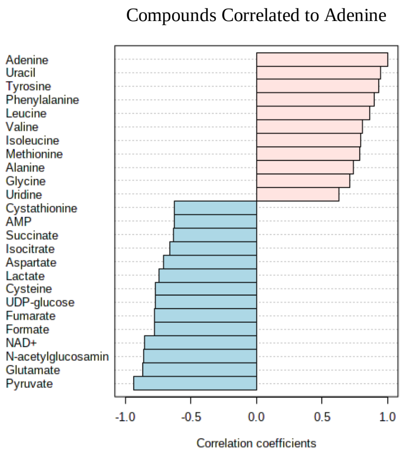
Compounds Correlated to Adenine
Adenine
Uracil
Tyrosine
Phenylalanine
Leucine
Valine
Isoleucine
Methionine
Alanine
Glycine
Uridine
Cystathionine
AMP
Succinate
Isocitrate
Aspartate
Lactate
Cysteine
UDP-glucose
Fumarate
Formate
NAD+
N-acetylglucosamin
Glutamate
Pyruvate
-1.0 -0.5 0.0 0.5 1.0
Correlation coefficients

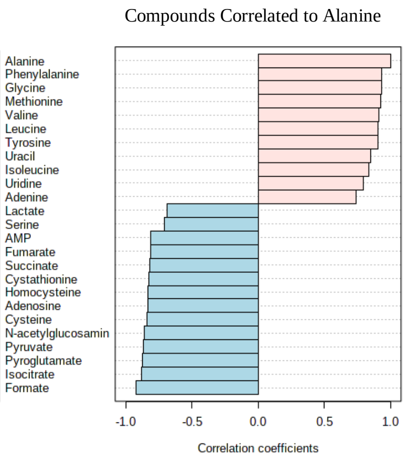
Compounds Correlated to Alanine
Alanine
Phenylalanine
Glycine
Methionine
Valine
Leucine
Tyrosine
Uracil
Isoleucine
Uridine
Adenine
Lactate
Serine
AMP
Fumarate
Succinate
Cystathionine
Homocysteine
Adenosine
Cysteine
N-acetylglucosamin
Pyruvate
Pyroglutamate
Isocitrate
Formate
-1.0 -0.5 0.0 0.5 1.0
Correlation coefficients

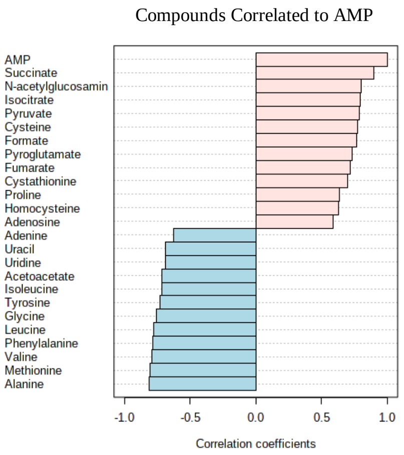
Compounds Correlated to AMP
AMP
Succinate
N-acetylglucosamin
Isocitrate
Pyruvate
Cysteine
Formate
Pyroglutamate
Fumarate
Cystathionine
Proline
Homocysteine
Adenosine
Adenine
Uracil
Uridine
Acetoacetate
Isoleucine
Tyrosine
Glycine
Leucine
Phenylalanine
Valine
Methionine
Alanine
-1.0 -0.5 0.0 0.5 1.0
Correlation coefficients

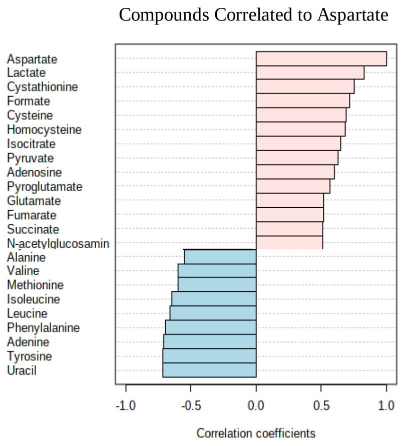
Compounds Correlated to Aspartate
Aspartate
Lactate
Cystathionine
Formate
Cysteine
Homocysteine
Isocitrate
Pyruvate
Adenosine
Pyroglutamate
Glutamate
Fumarate
Succinate
N-acetylglucosamin
Alanine
Valine
Methionine
Isoleucine
Leucine
Phenylalanine
Adenine
Tyrosine
Uracil
-1.0 -0.5 0.0 0.5 1.0
Correlation coefficients

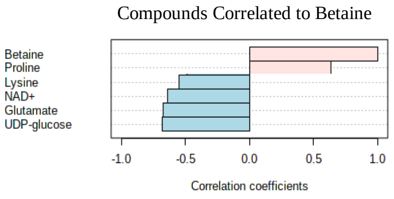
Compounds Correlated to Betaine
Betaine
Proline
Lysine
NAD+
Glutamate
UDP-glucose
-1.0 -0.5 0.0 0.5 1.0
Correlation coefficients

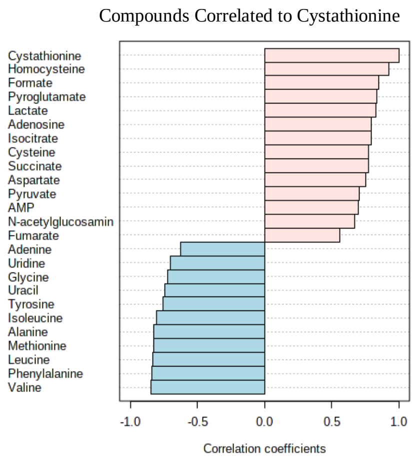
Compounds Correlated to Cystathionine
Cystathionine
Homocysteine
Formate
Pyroglutamate
Lactate
Adenosine
Isocitrate
Cysteine
Succinate
Aspartate
Pyruvate
AMP
N-acetylglucosamin
Fumarate
Adenine
Uridine
Glycine
Uracil
Tyrosine
Isoleucine
Alanine
Methionine
Leucine
Phenylalanine
Valine
-1.0 -0.5 0.0 0.5 1.0
Correlation coefficients

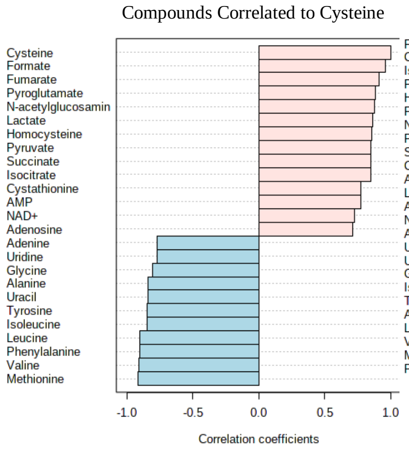
Compounds Correlated to Cysteine
Cysteine
Formate
Fumarate
Pyroglutamate
N-acetylglucosamin
Lactate
Homocysteine
Pyruvate
Succinate
Isocitrate
Cystathionine
AMP
NAD+
Adenosine
Adenine
Uridine
Glycine
Alanine
Uracil
Tyrosine
Isoleucine
Leucine
Phenylalanine
Valine
Methionine
-1.0 -0.5 0.0 0.5 1.0
Correlation coefficients

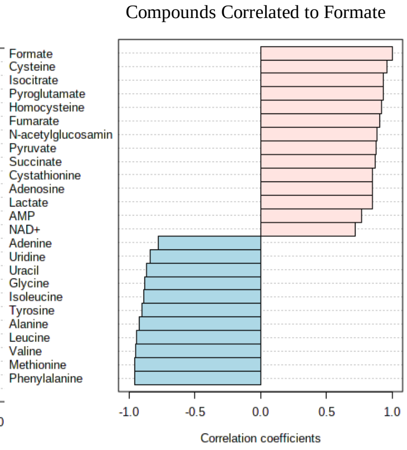
Compounds Correlated to Formate
Formate
Cysteine
Isocitrate
Pyroglutamate
Homocysteine
Fumarate
N-acetylglucosamin
Pyruvate
Succinate
Cystathionine
Adenosine
Lactate
AMP
NAD+
Adenine
Uridine
Uracil
Glycine
Isoleucine
Tyrosine
Alanine
Leucine
Valine
Methionine
Phenylalanine
-1.0 -0.5 0.0 0.5 1.0
Correlation coefficients

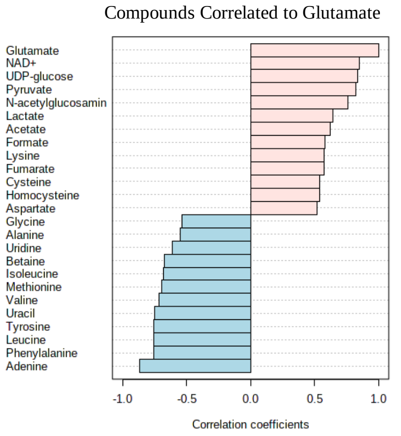
Compounds Correlated to Glutamate
Glutamate
NAD+
UDP-glucose
Pyruvate
N-acetylglucosamin
Lactate
Acetate
Formate
Lysine
Fumarate
Cysteine
Homocysteine
Aspartate
Glycine
Alanine
Uridine
Betaine
Isoleucine
Methionine
Valine
Uracil
Tyrosine
Leucine
Phenylalanine
Adenine
-1.0 -0.5 0.0 0.5 1.0
Correlation coefficients

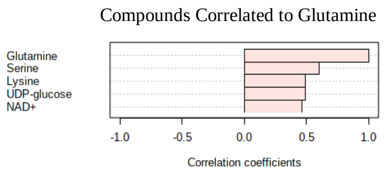
Compounds Correlated to Glutamine
Glutamine
Serine
Lysine
UDP-glucose
NAD+
-1.0 -0.5 0.0 0.5 1.0
Correlation coefficients

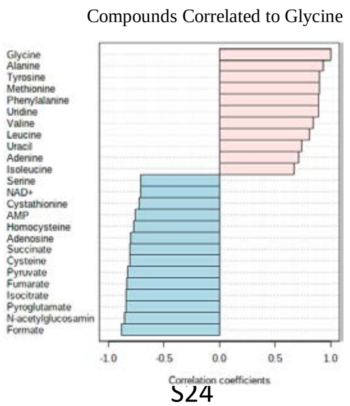
Compounds Correlated to Glycine
Glycine
Alanine
Tyrosine
Methionine
Phenylalanine
Uridine
Valine
Leucine
Uracil
Adenine
Isoleucine
Serine
NAD+
Cystathionine
AMP
Homocysteine
Adenosine
Succinate
Cysteine
Pyruvate
Fumarate
Isocitrate
Pyroglutamate
N-acetylglucosamin
Formate
-1.0 -0.5 0.0 0.5 1.0
Correlation coefficients

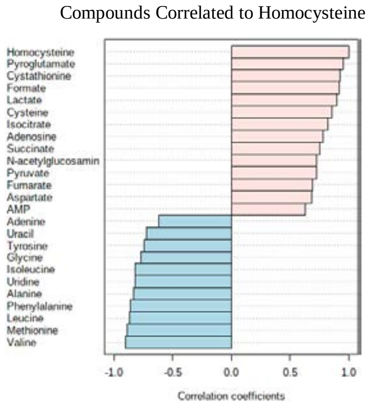
Compounds Correlated to Homocysteine
Homocysteine
Pyroglutamate
Cystathionine
Formate
Lactate
Cysteine
Isocitrate
Adenosine
Succinate
N-acetylglucosamin
Pyruvate
Fumarate
Aspartate
AMP
Adenine
Uracil
Tyrosine
Glycine
Isoleucine
Uridine
Alanine
Phenylalanine
Leucine
Methionine
Valine
-1.0 -0.5 0.0 0.5 1.0
Correlation coefficients

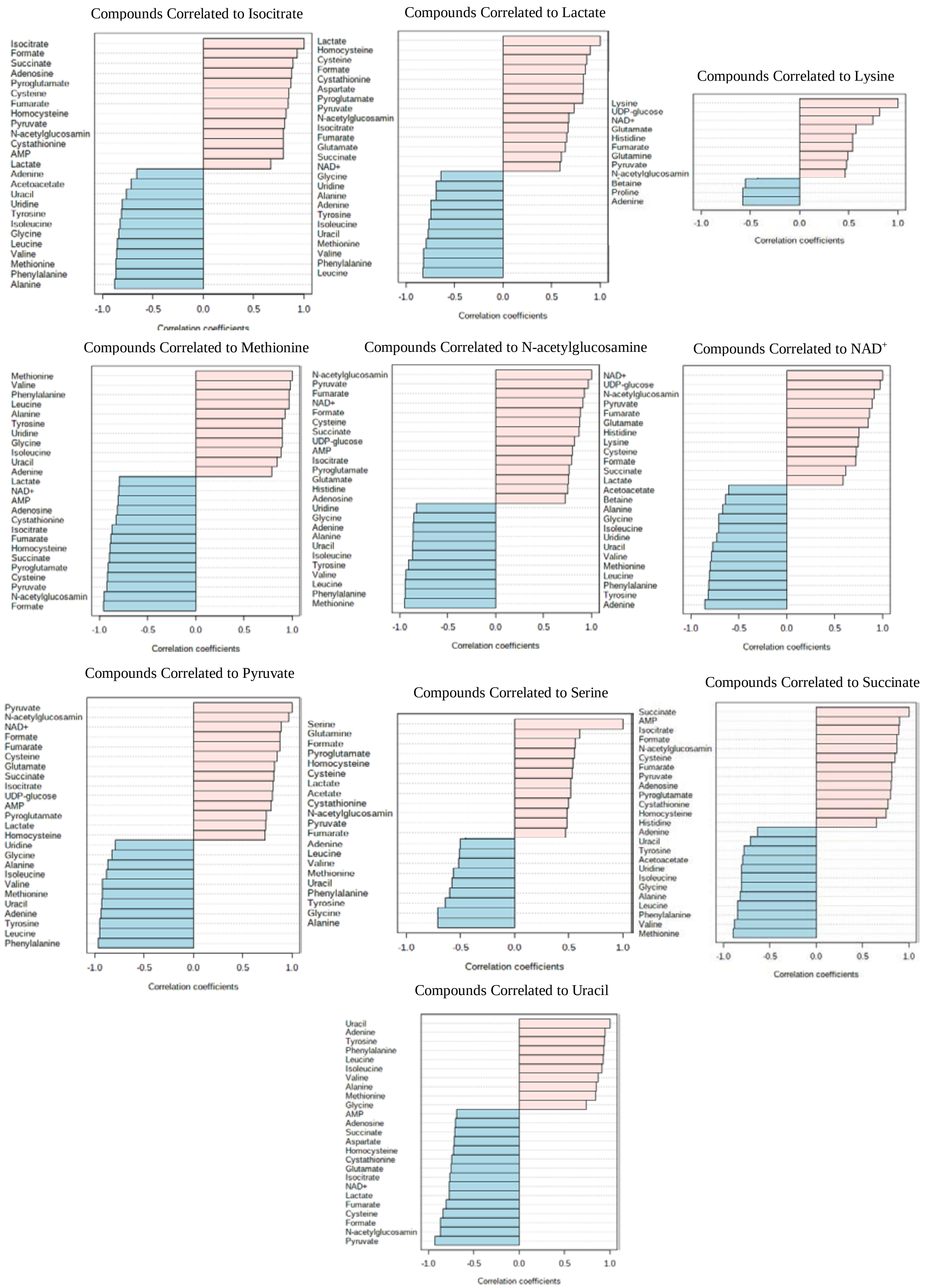

Figure S17. Pattern Hunter Correlations between Metabolites Observed in the Mid Log Phase Comparison of Challenged Mutant and Unchallenged Wild Type Samples.

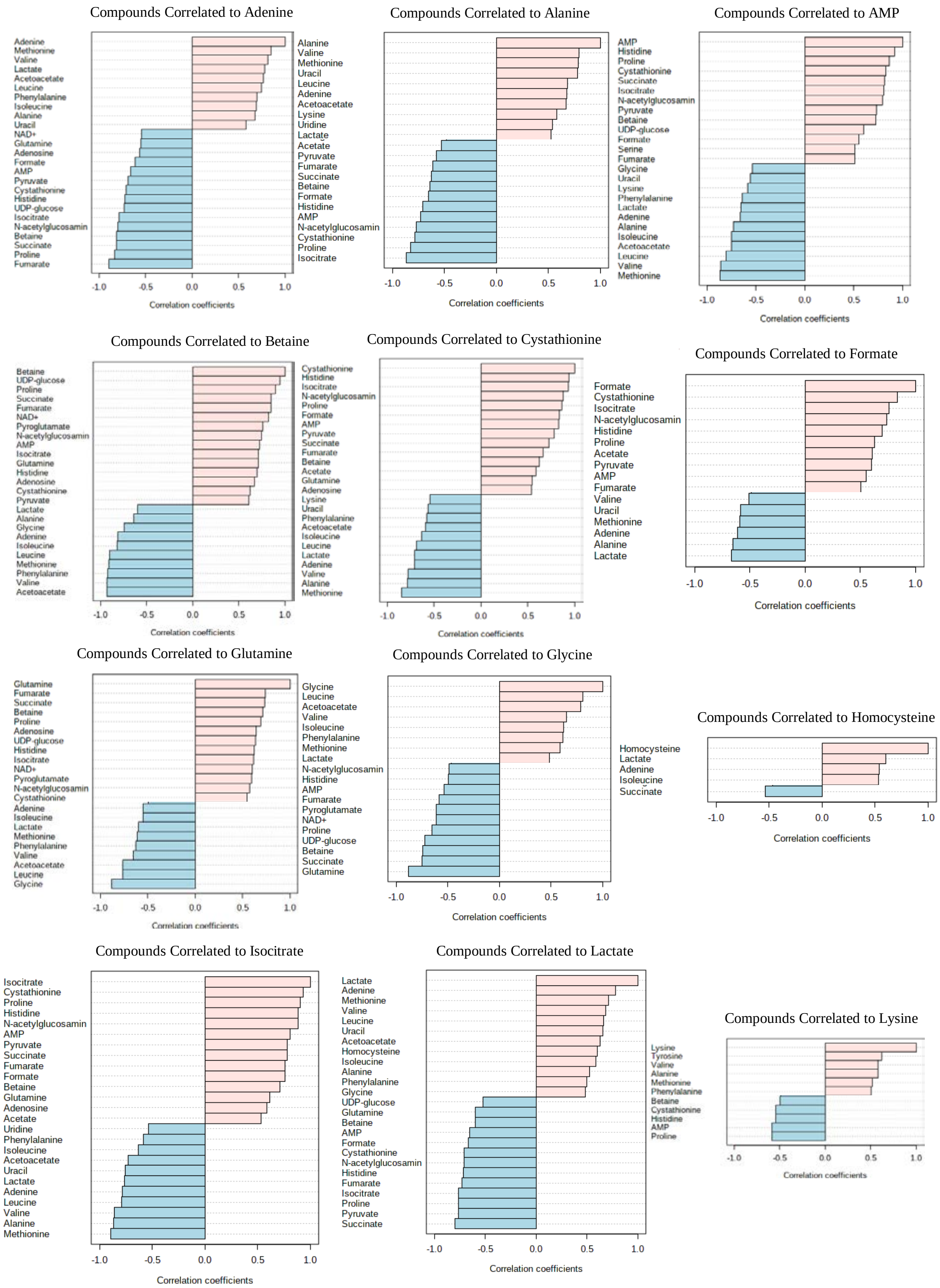
Compounds Correlated to Adenine
Adenine
Methionine
Valine
Lactate
Acetoacetate
Leucine
Phenylalanine
Isoleucine
Alanine
Uracil
NAD+
Glutamine
Adenosine
Formate
AMP
Pyruvate
Cystathionine
Histidine
UDP-glucose
Isocitrate
N-acetylglucosamin
Betaine
Succinate
Proline
Fumarate
Alanine
Valine
Methionine
Uracil
Leucine
Adenine
Acetoacetate
Lysine
Uridine
Lactate
Acetate
Pyruvate
Fumarate
Succinate
Betaine
Formate
Histidine
AMP
N-acetylglucosamin
Cystathionine
Proline
Isocitrate
-1.0
-0.5
0.0
0.5
1.0
Correlation coefficients
Compounds Correlated to Alanine
AMP
Histidine
Proline
Cystathionine
Succinate
Isocitrate
N-acetylglucosamin
Pyruvate
Betaine
UDP-glucose
Formate
Serine
Fumarate
Glycine
Uracil
Lysine
Phenylalanine
Lactate
Adenine
Alanine
Isoleucine
Acetoacetate
Leucine
Valine
Methionine
-1.0
-0.5
0.0
0.5
1.0
Correlation coefficients
Compounds Correlated to AMP
-1.0
-0.5
0.0
0.5
1.0
Correlation coefficients
Compounds Correlated to Betaine
Betaine
UDP-glucose
Proline
Succinate
Fumarate
NAD+
Pyroglutamate
N-acetylglucosamin
AMP
Isocitrate
Glutamine
Histidine
Adenosine
Cystathionine
Pyruvate
Lactate
Alanine
Glycine
Adenine
Isoleucine
Leucine
Methionine
Phenylalanine
Valine
Acetoacetate
-1.0
-0.5
0.0
0.5
1.0
Correlation coefficients
Compounds Correlated to Cystathionine
Cystathionine
Histidine
Isocitrate
N-acetylglucosamin
Proline
Formate
AMP
Pyruvate
Succinate
Fumarate
Betaine
Acetate
Glutamine
Adenosine
Lysine
Uracil
Phenylalanine
Acetoacetate
Isoleucine
Leucine
Lactate
Adenine
Valine
Alanine
Methionine
-1.0
-0.5
0.0
0.5
1.0
Correlation coefficients
Compounds Correlated to Formate
Formate
Cystathionine
Isocitrate
N-acetylglucosamin
Histidine
Proline
Acetate
Pyruvate
AMP
Fumarate
Valine
Uracil
Methionine
Adenine
Alanine
Lactate
-1.0
-0.5
0.0
0.5
1.0
Correlation coefficients
Compounds Correlated to Glutamine
Glutamine
Fumarate
Succinate
Betaine
Proline
Adenosine
UDP-glucose
Histidine
Isocitrate
NAD+
Pyroglutamate
N-acetylglucosamin
Cystathionine
Adenine
Isoleucine
Lactate
Methionine
Phenylalanine
Valine
Acetoacetate
Leucine
Glycine
-1.0
-0.5
0.0
0.5
1.0
Correlation coefficients
Compounds Correlated to Glycine
Glycine
Leucine
Acetoacetate
Valine
Isoleucine
Phenylalanine
Methionine
Lactate
N-acetylglucosamin
Histidine
AMP
Fumarate
Pyroglutamate
NAD+
Proline
UDP-glucose
Betaine
Succinate
Glutamine
-1.0
-0.5
0.0
0.5
1.0
Correlation coefficients
Compounds Correlated to Homocysteine
Homocysteine
Lactate
Adenine
Isoleucine
Succinate
-1.0
-0.5
0.0
0.5
1.0
Correlation coefficients
Compounds Correlated to Isocitrate
Isocitrate
Cystathionine
Proline
Histidine
N-acetylglucosamin
AMP
Pyruvate
Succinate
Fumarate
Formate
Betaine
Glutamine
Adenosine
Acetate
Uridine
Phenylalanine
Isoleucine
Acetoacetate
Uracil
Lactate
Adenine
Leucine
Valine
Alanine
Methionine
-1.0
-0.5
0.0
0.5
1.0
Correlation coefficients
Compounds Correlated to Lactate
Lactate
Adenine
Methionine
Valine
Leucine
Uracil
Acetoacetate
Homocysteine
Isoleucine
Alanine
Phenylalanine
Glycine
UDP-glucose
Glutamine
Betaine
AMP
Formate
Cystathionine
N-acetylglucosamin
Histidine
Fumarate
Isocitrate
Proline
Pyruvate
Succinate
-1.0
-0.5
0.0
0.5
1.0
Correlation coefficients
Compounds Correlated to Lysine
Lysine
Tyrosine
Valine
Alanine
Methionine
Phenylalanine
Betaine
Cystathionine
Histidine
AMP
Proline
-1.0
-0.5
0.0
0.5
1.0
Correlation coefficients

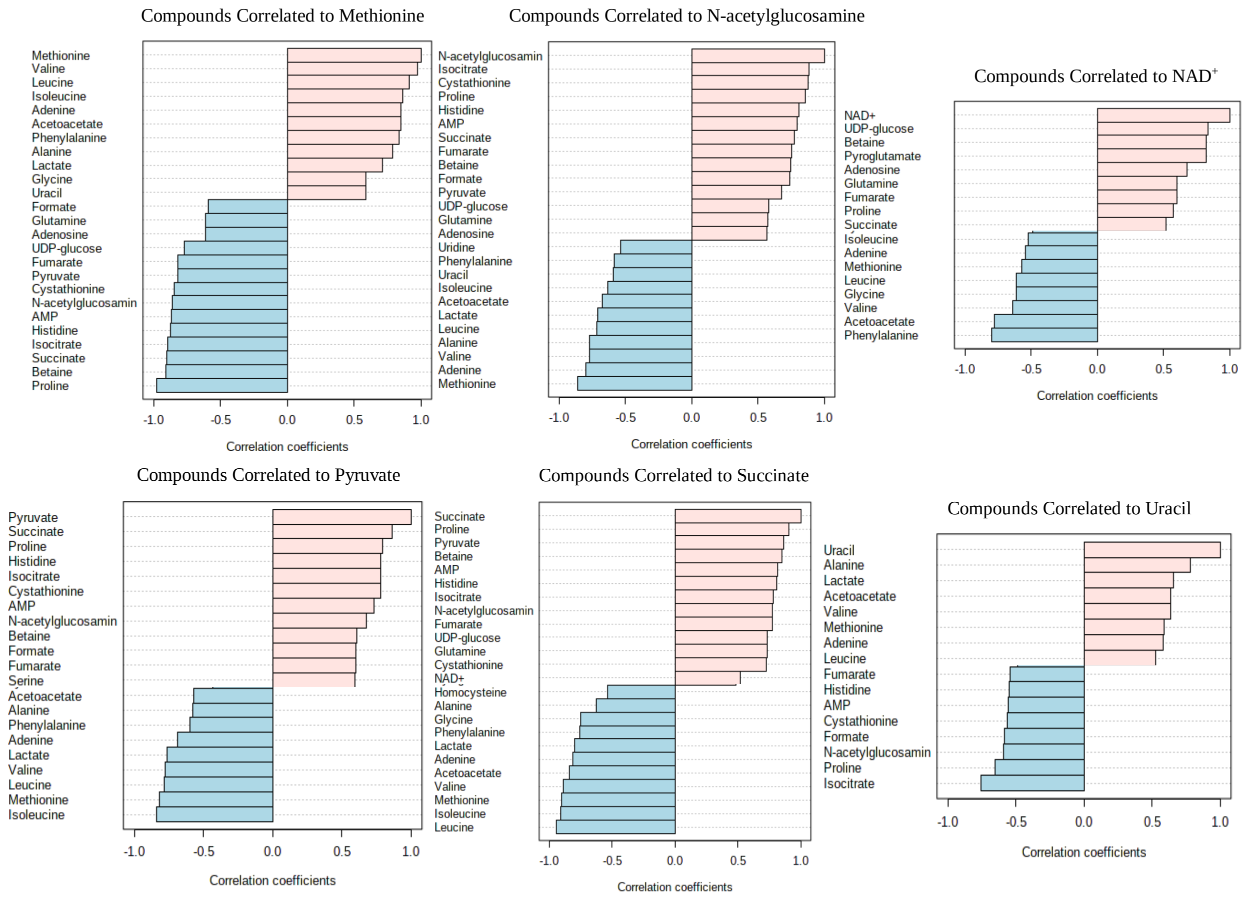

Figure S18. Pattern Hunter Correlations between Metabolites Observed in the Mid Log Phase Comparison of Unchallenged Mutant and Challenged Wild Type Samples.

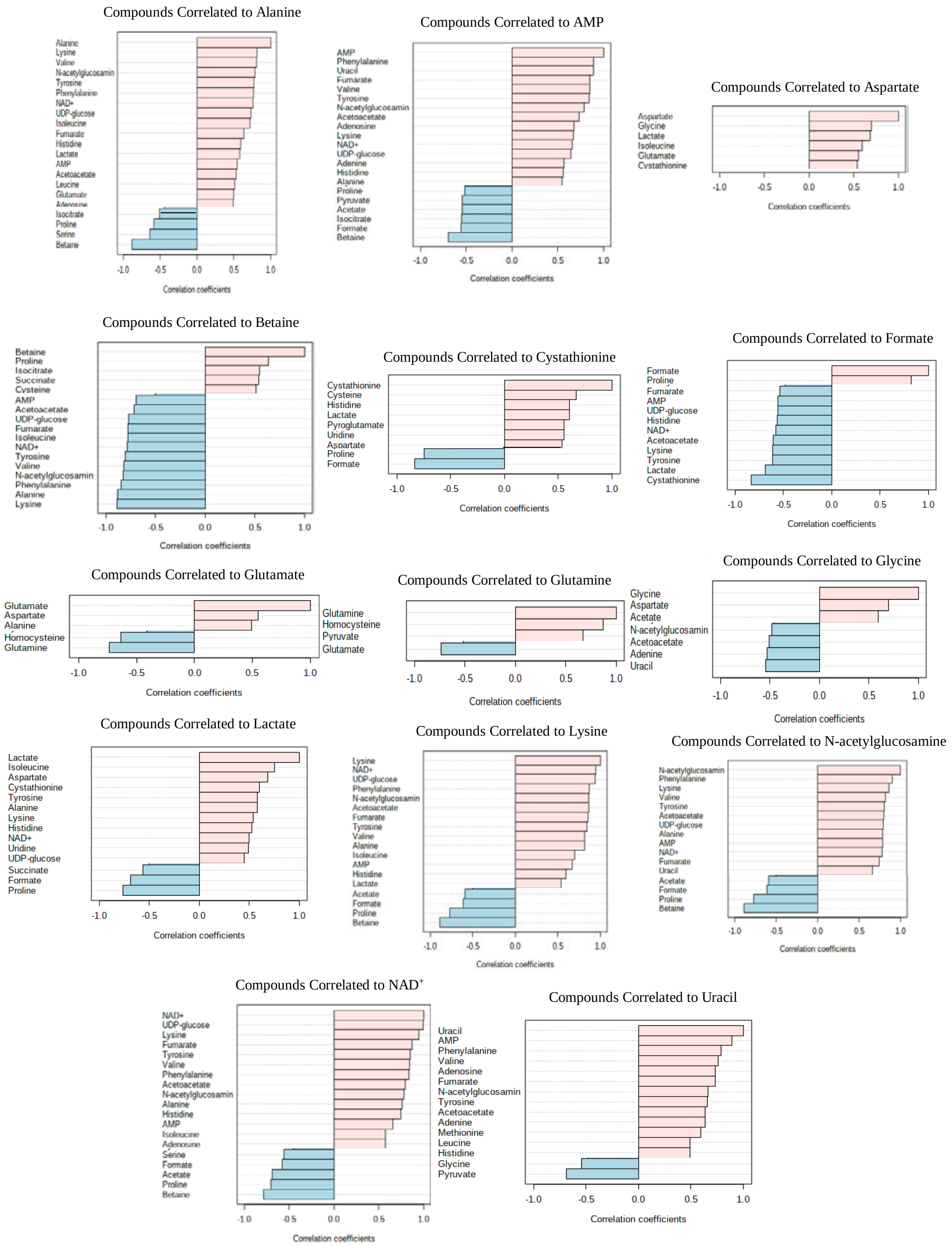

Figure S19. Pattern Hunter Correlations between Metabolites Observed in the Stationary Phase Comparison of Unchallenged Mutant and Unchallenged Wild Type Samples.

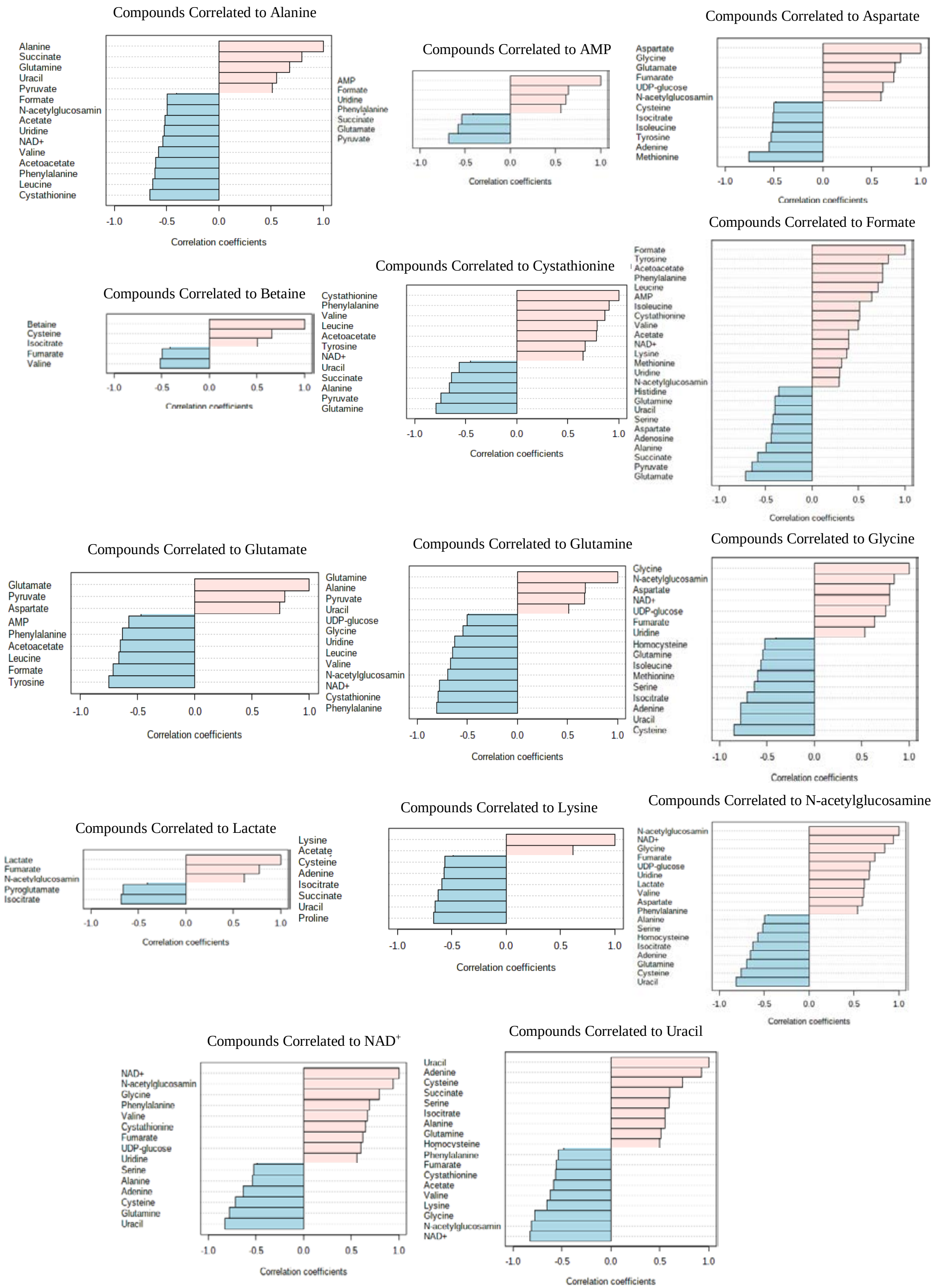

Figure S20. Pattern Hunter Correlations between Metabolites Observed in the Stationary Phase Comparison of Challenged Mutant and Challenged Wild Type Samples.

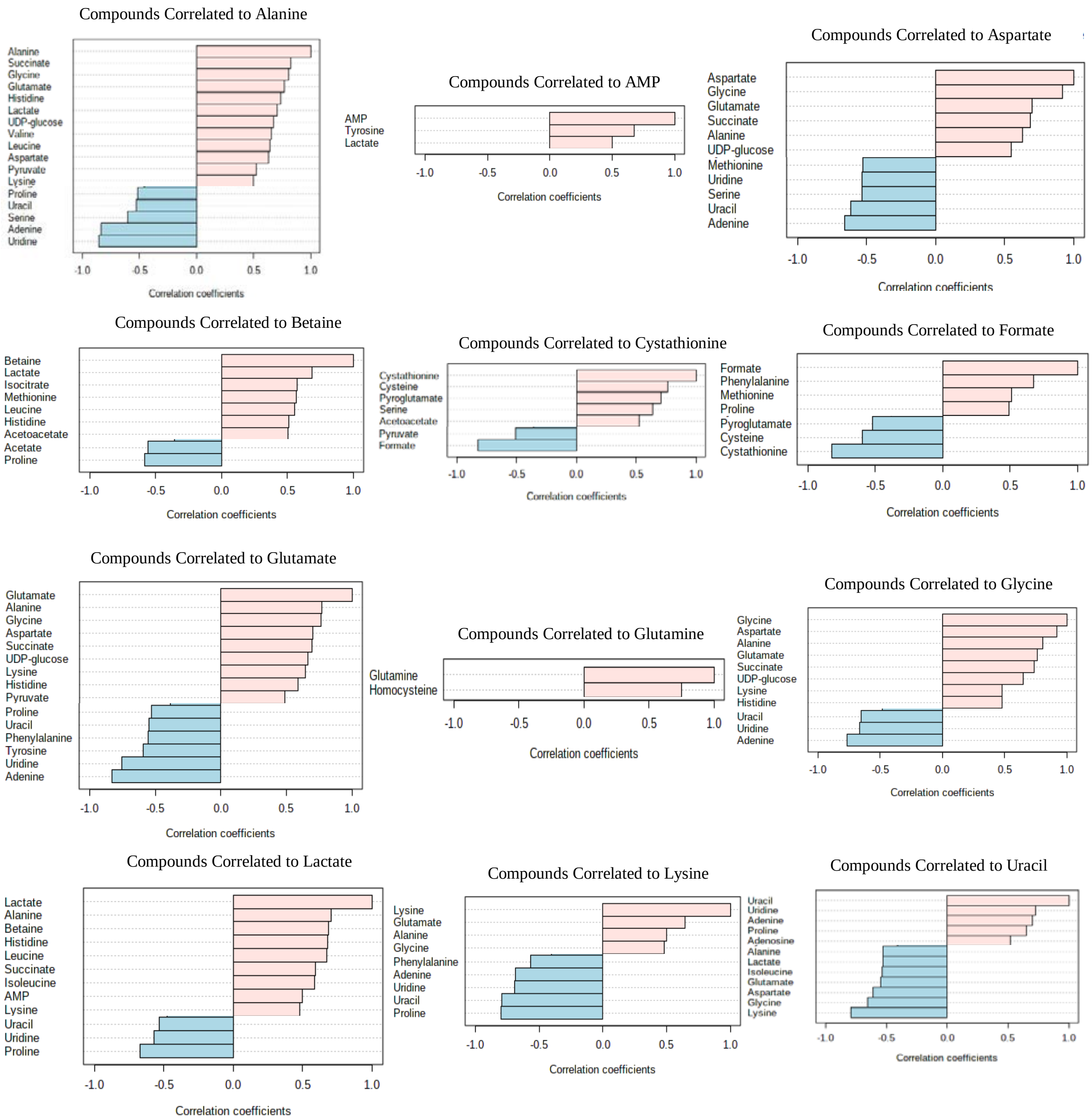

Figure S21. Pattern Hunter Correlations between Metabolites Observed in the Stationary Phase Comparison of Challenged and Unchallenged Wild Type Samples.

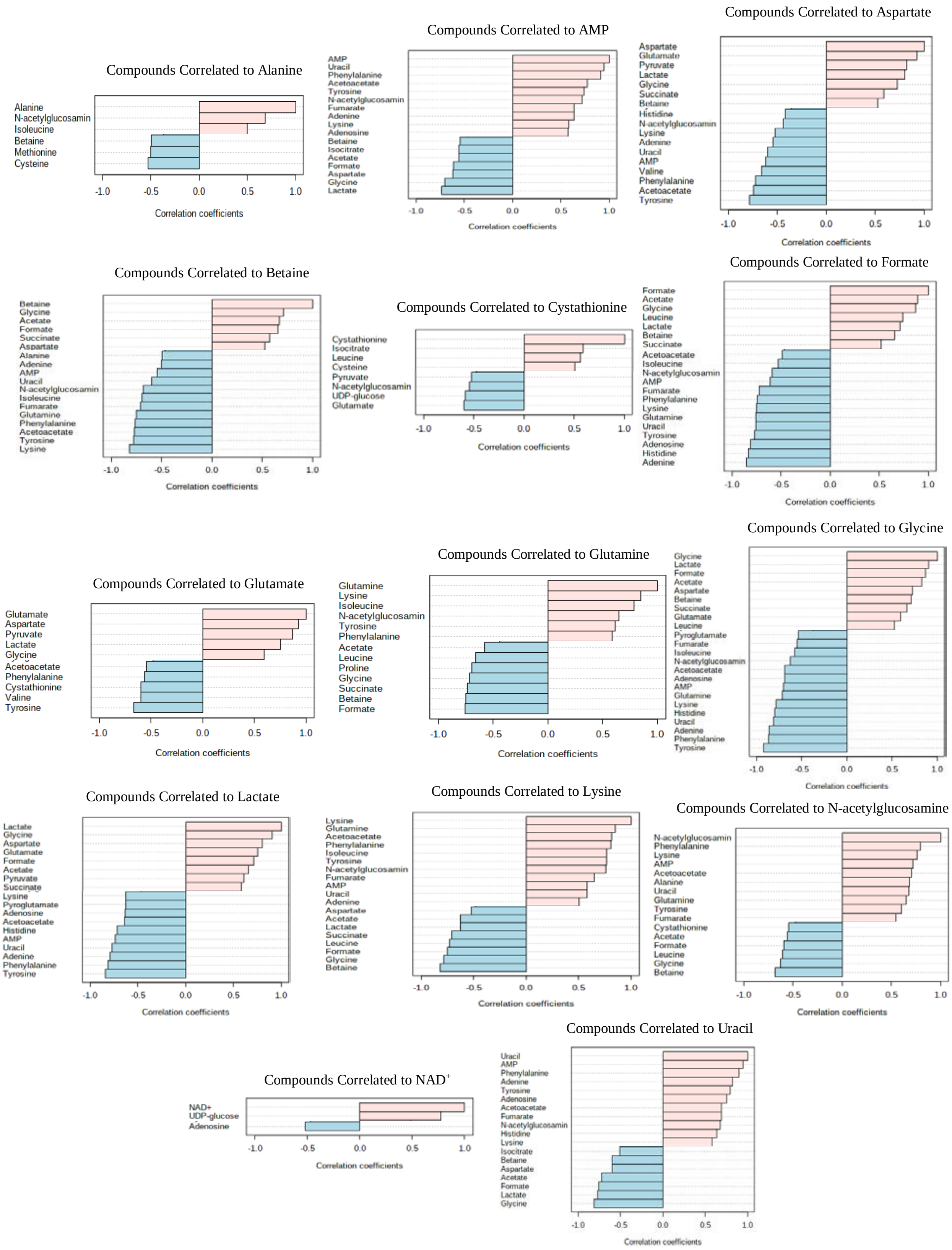

Figure S22. Pattern Hunter Correlations between Metabolites Observed in the Stationary Phase Comparison of Challenged and Unchallenged Mutant Samples.

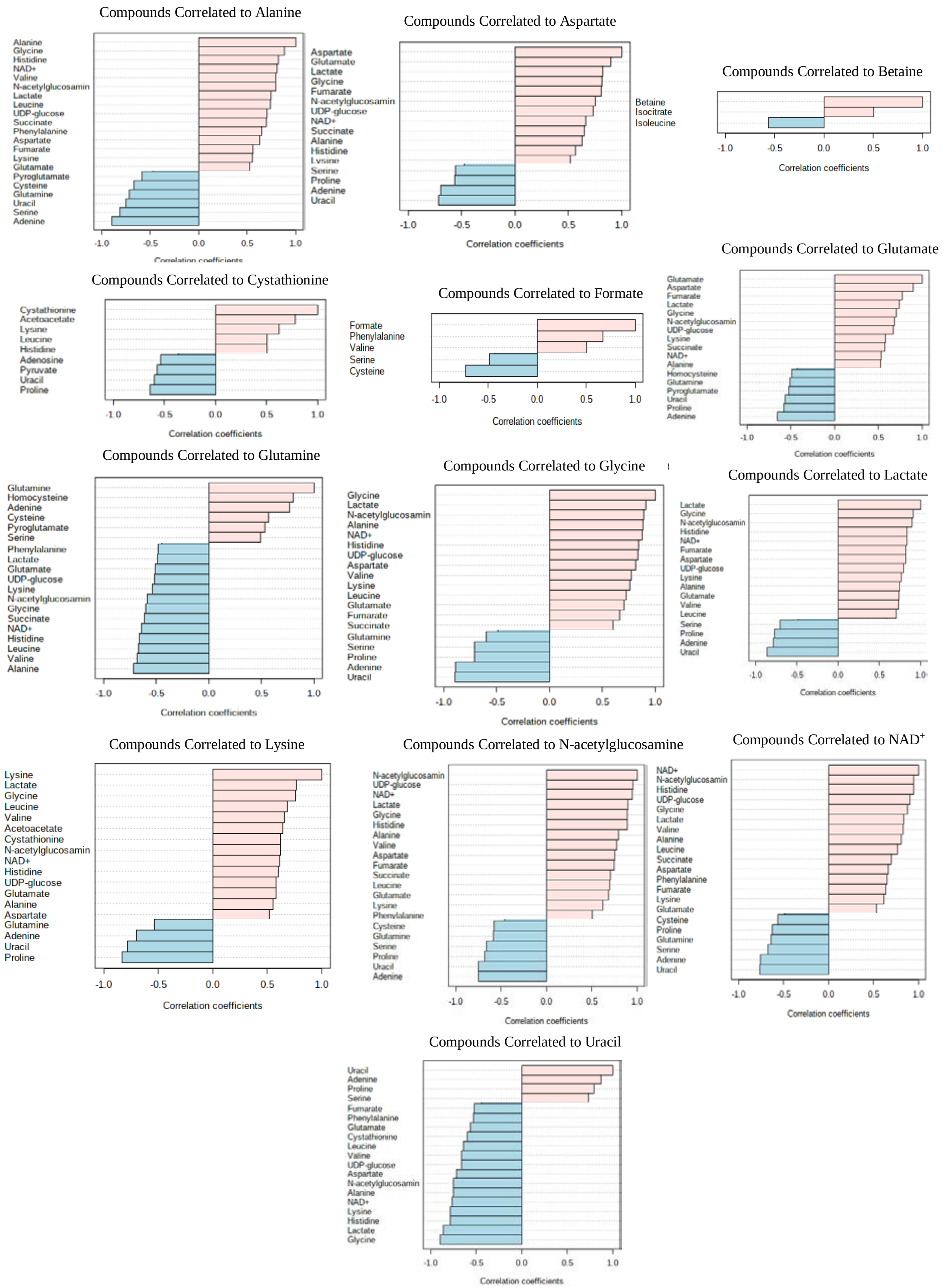

Figure S23. Pattern Hunter Correlations between Metabolites Observed in the Stationary Phase Comparison of Challenged Mutant and Unchallenged Wild Type Samples.

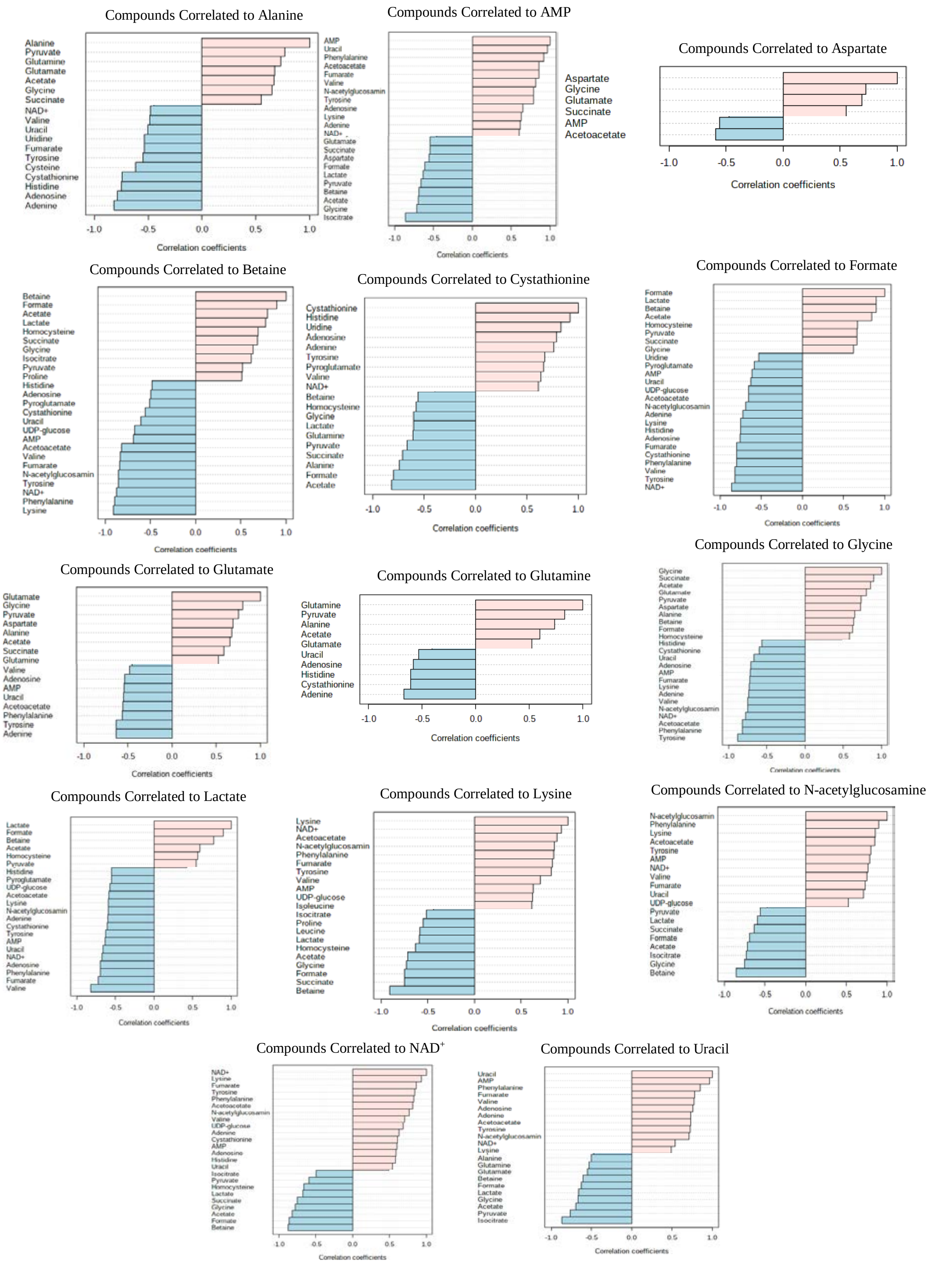

Figure S24. Pattern Hunter Correlations between Metabolites Observed in the Stationary Phase Comparison of Challenged Wild Type and Unchallenged Mutant Samples.

Table S4. The Identification Number Corresponding to Pathways Found in Pathway Analysis for Figures S25 – S36.

Identity Number	Pathways
1	Aminoacyl-tRNA Biosynthesis
2	Alanine Metabolism
3	Methionine Metabolism
4	Glycine Metabolism
5	Nucleotide Metabolism
6	Glutamine and Glutamate Metabolism
7	Valine, Leucine, and Isoleucine Biosynthesis
8	Acetyl-CoA Biosynthesis
9	Pyruvate Metabolism
10	Citric Acid Cycle

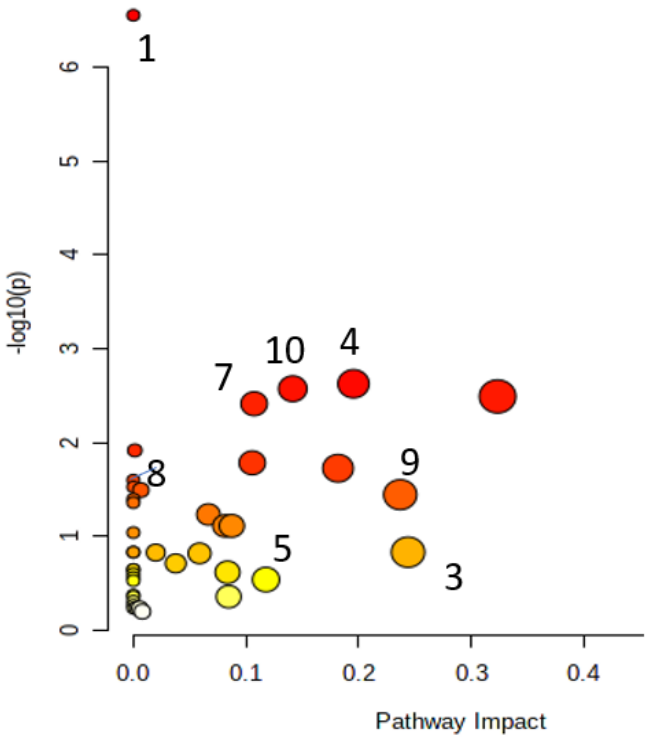

Figure S25. Top Impacted Pathways for Mid Log Phase Comparison of Unchallenged Mutant and Unchallenged Wild Type Samples. The larger and darker the circles are, the larger the impact the significant metabolites have on the listed pathway.

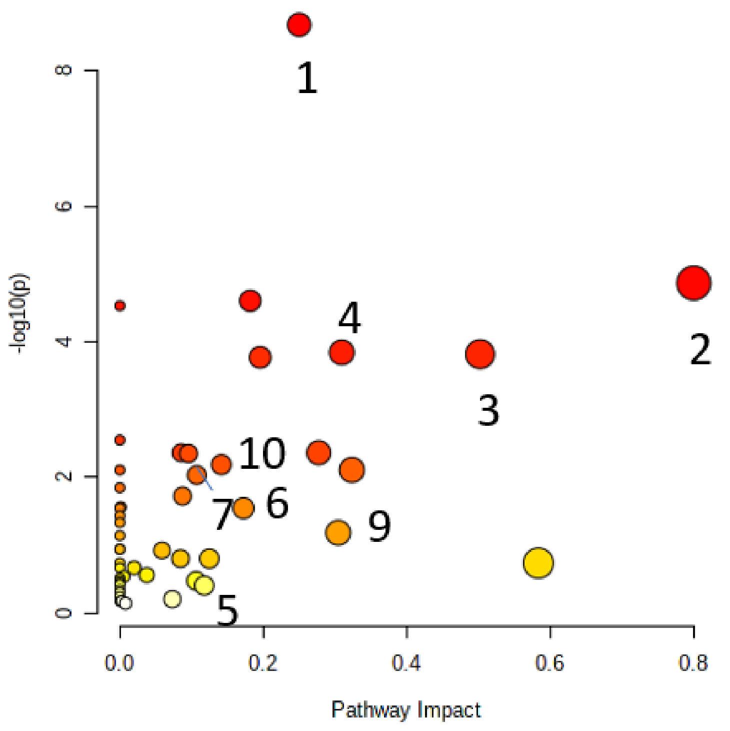

Figure S26. Top Impacted Pathways for Mid Log Phase Comparison of Challenged Mutant and Challenged Wild Type Samples. The larger and darker the circles are, the larger the impact the significant metabolites have on the listed pathway.

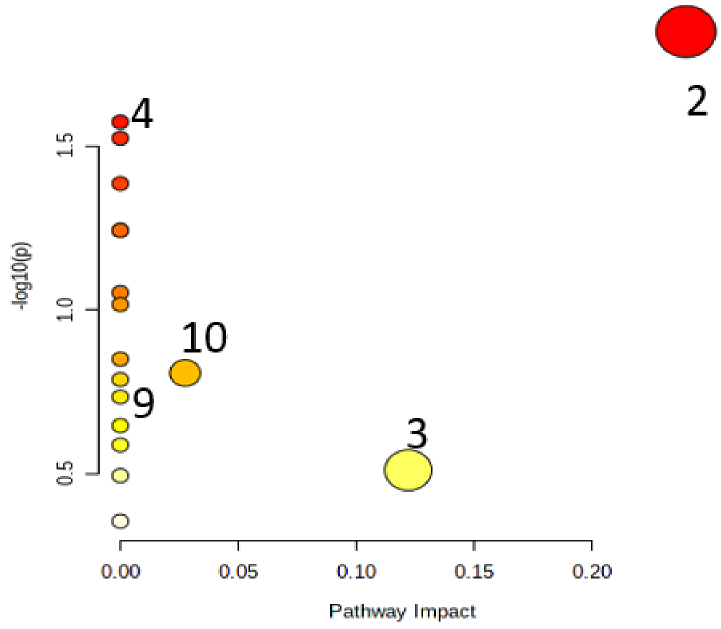

Figure S27. Top Impacted Pathways for Mid Log Phase Comparison of Challenged and Unchallenged Wild Type Samples. The larger and darker the circles are, the larger the impact the significant metabolites have on the listed pathway.

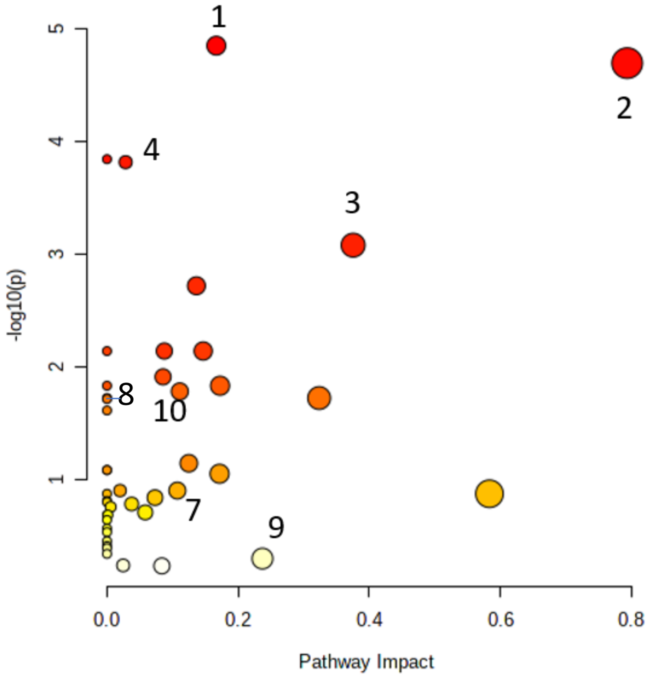

Figure S28. Top Impacted Pathways for Mid Log Phase Comparison of Challenged and Unchallenged Mutant Samples. The larger and darker the circles are, the larger the impact the significant metabolites have on the listed pathway.

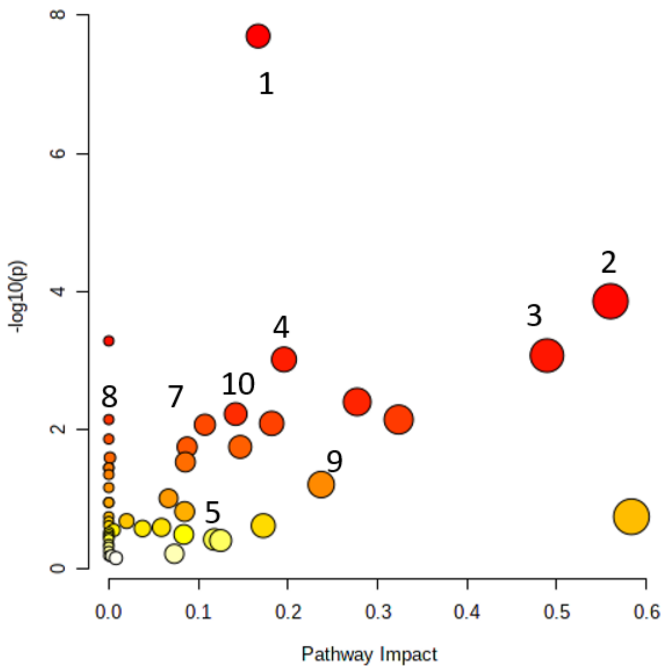

Figure S29. Top Impacted Pathways for Mid Log Phase Comparison of Challenged Mutant and Unchallenged Wild Type Samples. The larger and darker the circles are, the larger the impact the significant metabolites have on the listed pathway.

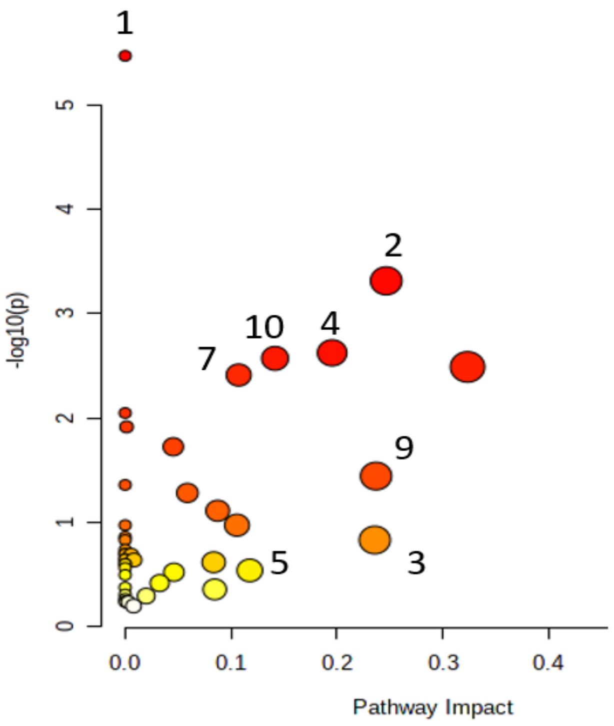

Figure S30. Top Impacted Pathways for Mid Log Phase Comparison of Challenged Wild Type and Unchallenged Mutant Samples. The larger and darker the circles are, the larger the impact the significant metabolites have on the listed pathway.

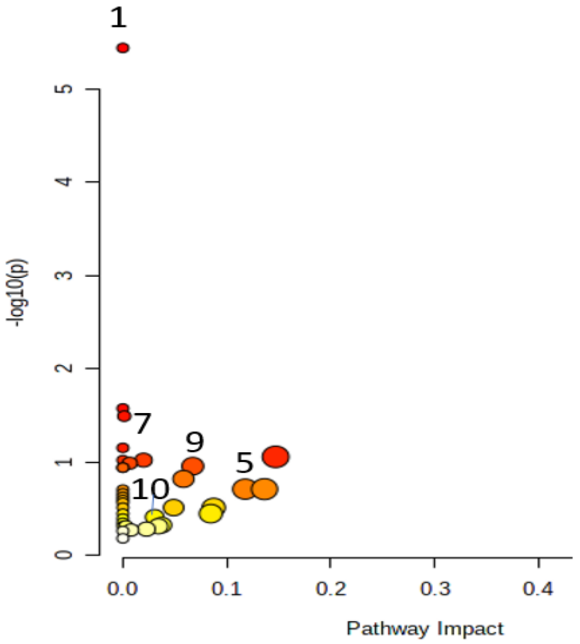

Figure S31. Top Impacted Pathways for Stationary Phase Comparison of Unchallenged Mutant and Unchallenged Wild Type Samples. The larger and darker the circles are, the larger the impact the significant metabolites have on the listed pathway.

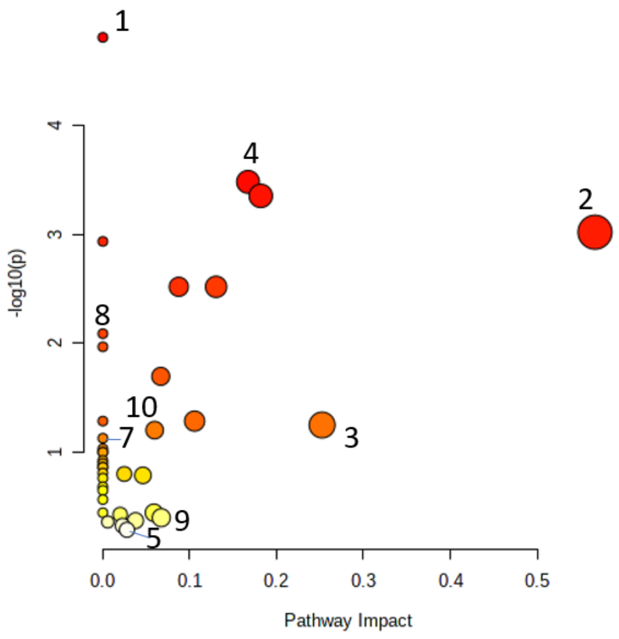

Figure S32. Top Impacted Pathways for Stationary Phase Comparison of Challenged Mutant and Challenged Wild Type Samples. The larger and darker the circles are, the larger the impact the significant metabolites have on the listed pathway.

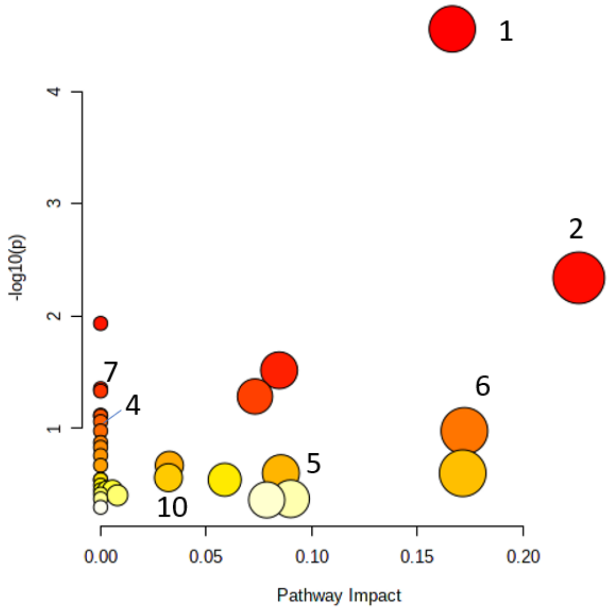

Figure S33. Top Impacted Pathways for Stationary Phase Comparison of Challenged and Unchallenged Wild Type Samples. The larger and darker the circles are, the larger the impact the significant metabolites have on the listed pathway.

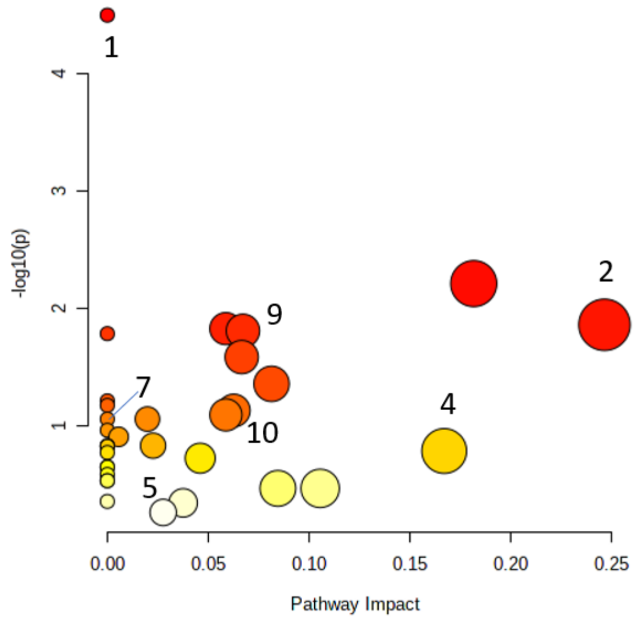

Figure S34. Top Impacted Pathways for Stationary Phase Comparison of Challenged and Unchallenged Mutant Samples. The larger and darker the circles are, the larger the impact the significant metabolites have on the listed pathway.

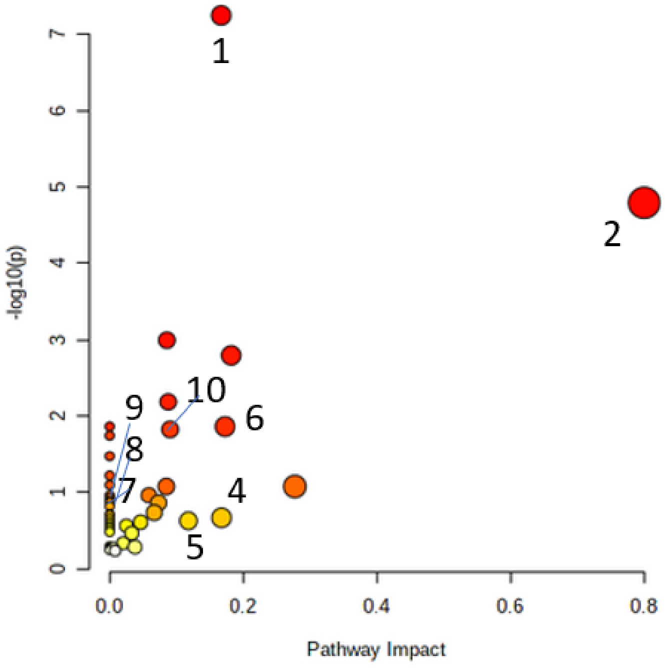

Figure S35. Top Impacted Pathways for Stationary Phase Comparison of Challenged Mutant and Unchallenged Wild Type Samples. The larger and darker the circles are, the larger the impact the significant metabolites have on the listed pathway.

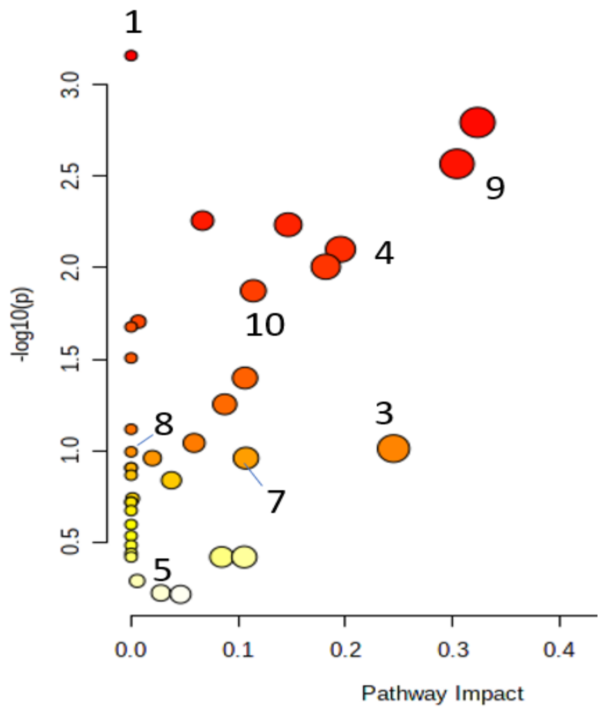

Figure S36. Top Impacted Pathways for Stationary Phase Comparison of Challenged Wild Type and Unchallenged Mutant Samples. The larger and darker the circles are, the larger the impact the significant metabolites have on the listed pathway.

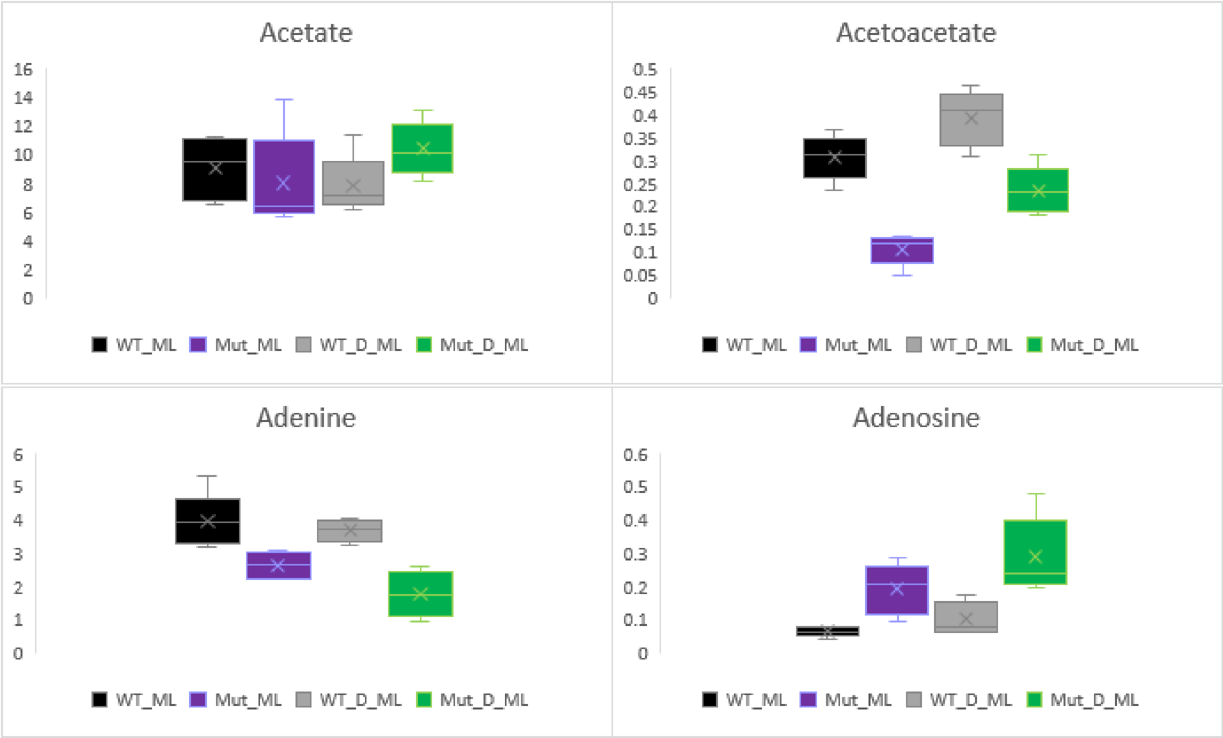

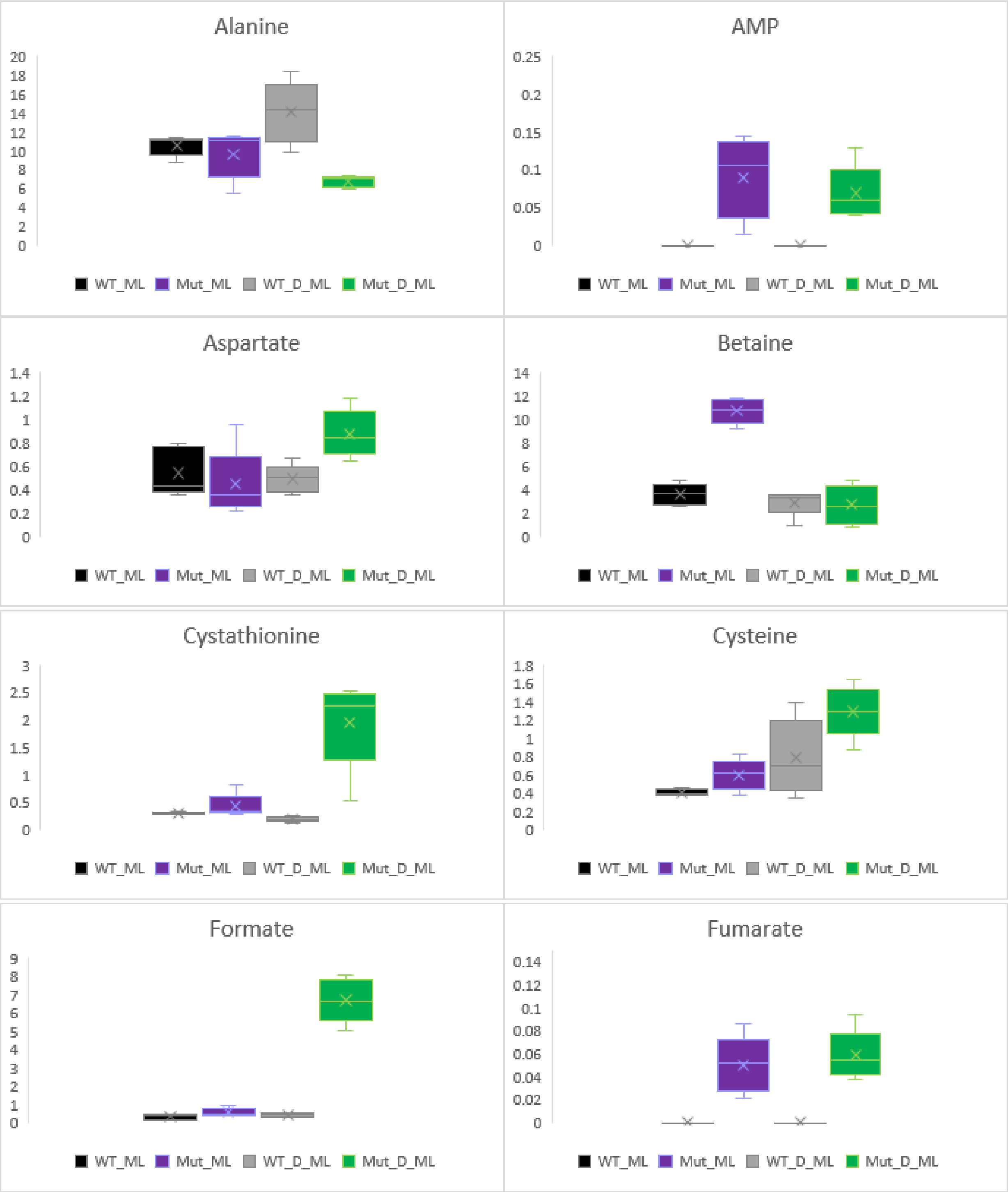
Alanine
20
18
16
14
12
10
8
6
4
2
0
WT_ML Mut_ML WT_D_ML Mut_D_ML
AMP
0.25
0.2
0.15
0.1
0.05
0
WT_ML Mut_ML WT_D_ML Mut_D_ML
Aspartate
1.4
1.2
1
0.8
0.6
0.4
0.2
0
WT_ML Mut_ML WT_D_ML Mut_D_ML
Betaine
14
12
10
8
6
4
2
0
WT_ML Mut_ML WT_D_ML Mut_D_ML
Cystathionine
3
2.5
2
1.5
1
0.5
0
WT_ML Mut_ML WT_D_ML Mut_D_ML
Cysteine
1.8
1.6
1.4
1.2
1
0.8
0.6
0.4
0.2
0
WT_ML Mut_ML WT_D_ML Mut_D_ML
Formate
9
8
7
6
5
4
3
2
1
0
WT_ML Mut_ML WT_D_ML Mut_D_ML
Fumarate
0.14
0.12
0.1
0.08
0.06
0.04
0.02
0
WT_ML Mut_ML WT_D_ML Mut_D_ML

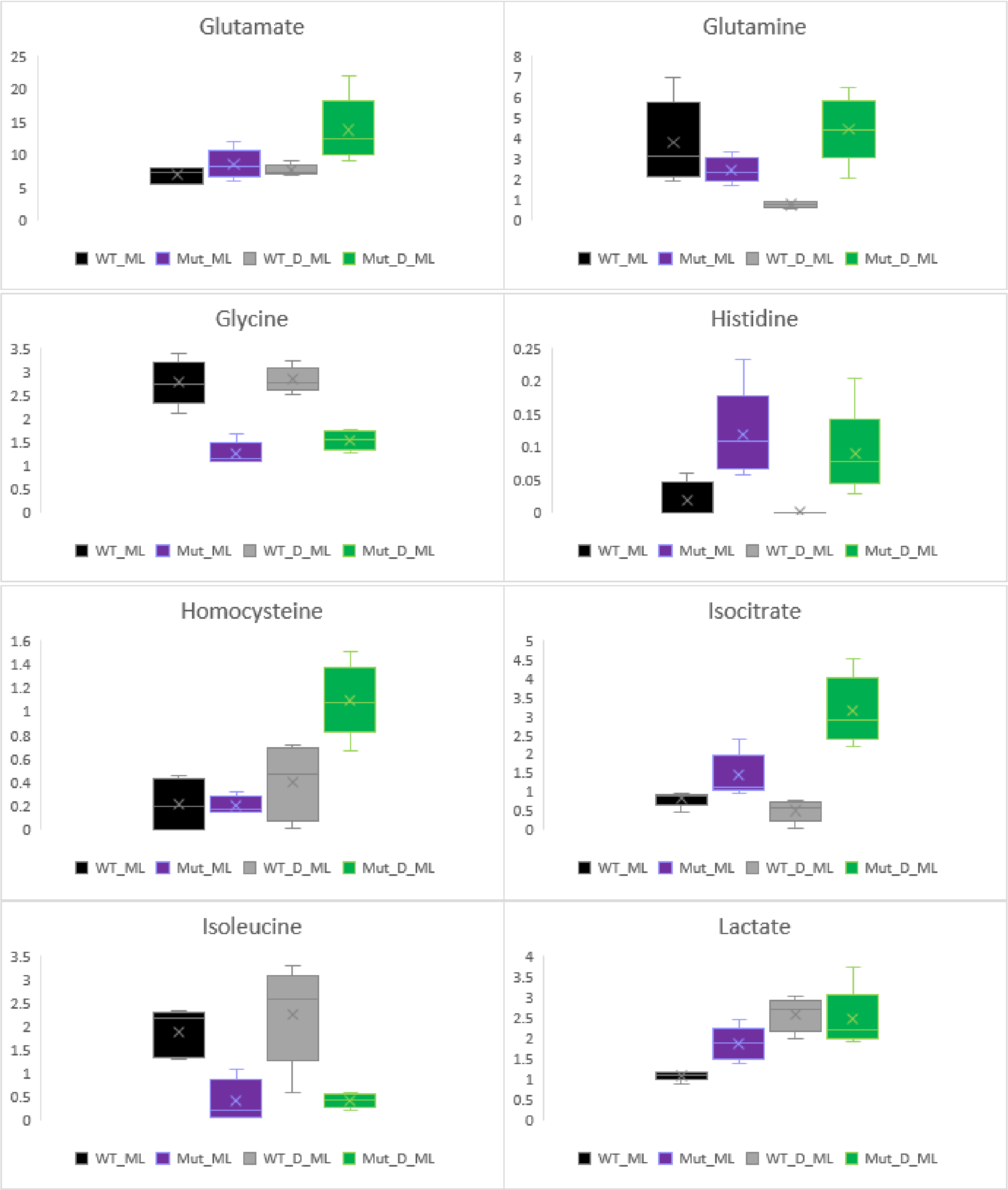
Glutamate
25
20
15
10
5
0
WT_ML
Mut_ML
WT_D_ML
Mut_D_ML
Glutamine
8
7
6
5
4
3
2
1
0
WT_ML
Mut_ML
WT_D_ML
Mut_D_ML
Glycine
3.5
3
2.5
2
1.5
1
0.5
0
WT_ML
Mut_ML
WT_D_ML
Mut_D_ML
Histidine
0.25
0.2
0.15
0.1
0.05
0
WT_ML
Mut_ML
WT_D_ML
Mut_D_ML
Homocysteine
1.6
1.4
1.2
1
0.8
0.6
0.4
0.2
0
WT_ML
Mut_ML
WT_D_ML
Mut_D_ML
Isocitrate
5
4.5
4
3.5
3
2.5
2
1.5
1
0.5
0
WT_ML
Mut_ML
WT_D_ML
Mut_D_ML
Isoleucine
3.5
3
2.5
2
1.5
1
0.5
0
WT_ML
Mut_ML
WT_D_ML
Mut_D_ML
Lactate
4
3.5
3
2.5
2
1.5
1
0.5
0
WT_ML
Mut_ML
WT_D_ML
Mut_D_ML

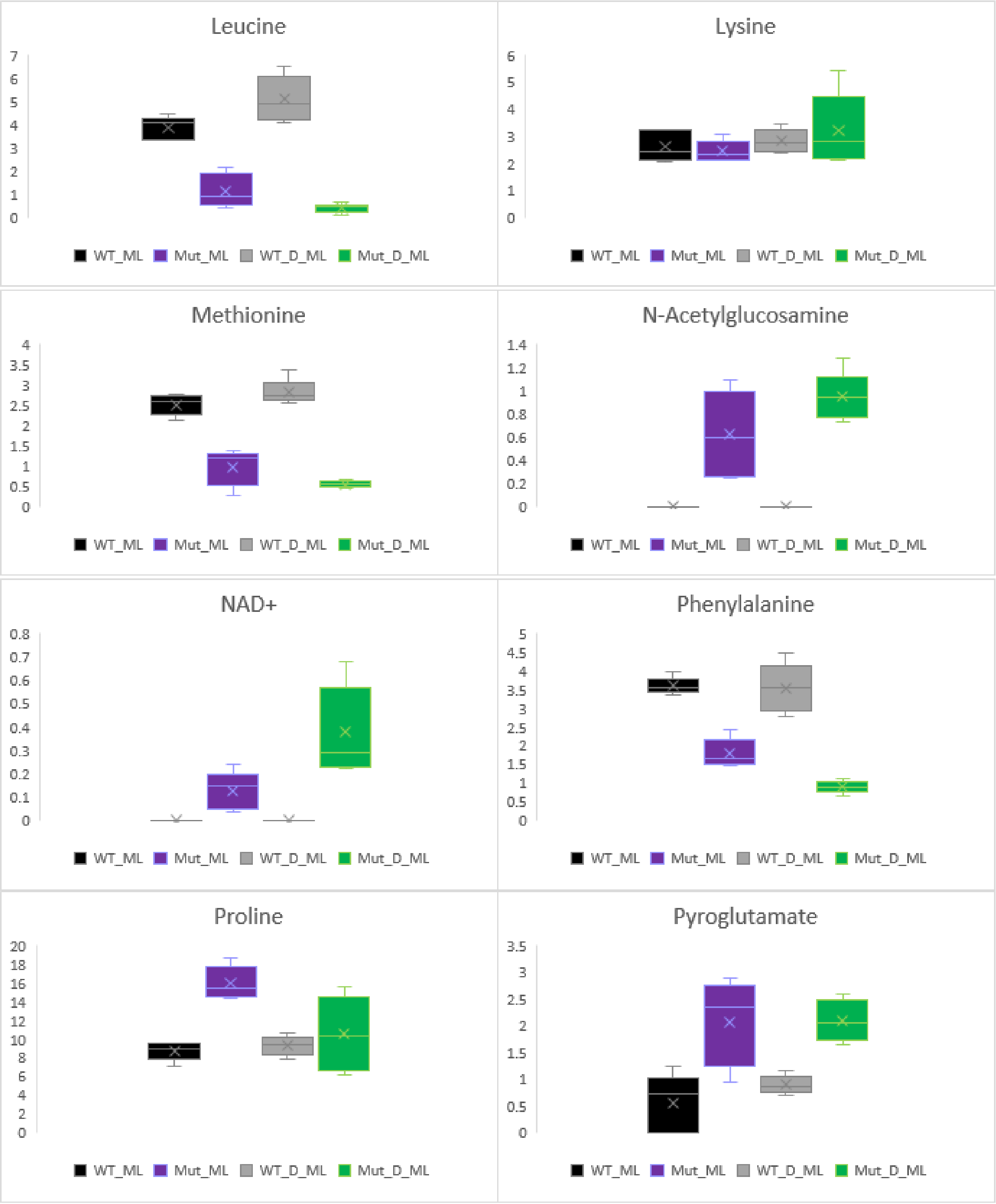
Leucine
7
6
5
4
3
2
1
0
WT_ML Mut_ML WT_D_ML Mut_D_ML
Lysine
6
5
4
3
2
1
0
WT_ML Mut_ML WT_D_ML Mut_D_ML
Methionine
4
3.5
3
2.5
2
1.5
1
0.5
0
WT_ML Mut_ML WT_D_ML Mut_D_ML
N-Acetylglucosamine
1.4
1.2
1
0.8
0.6
0.4
0.2
0
WT_ML Mut_ML WT_D_ML Mut_D_ML
NAD+
0.8
0.7
0.6
0.5
0.4
0.3
0.2
0.1
0
WT_ML Mut_ML WT_D_ML Mut_D_ML
Phenylalanine
5
4.5
4
3.5
3
2.5
2
1.5
1
0.5
0
WT_ML Mut_ML WT_D_ML Mut_D_ML
Proline
20
18
16
14
12
10
8
6
4
2
0
WT_ML Mut_ML WT_D_ML Mut_D_ML
Pyroglutamate
3.5
3
2.5
2
1.5
1
0.5
0
WT_ML Mut_ML WT_D_ML Mut_D_ML

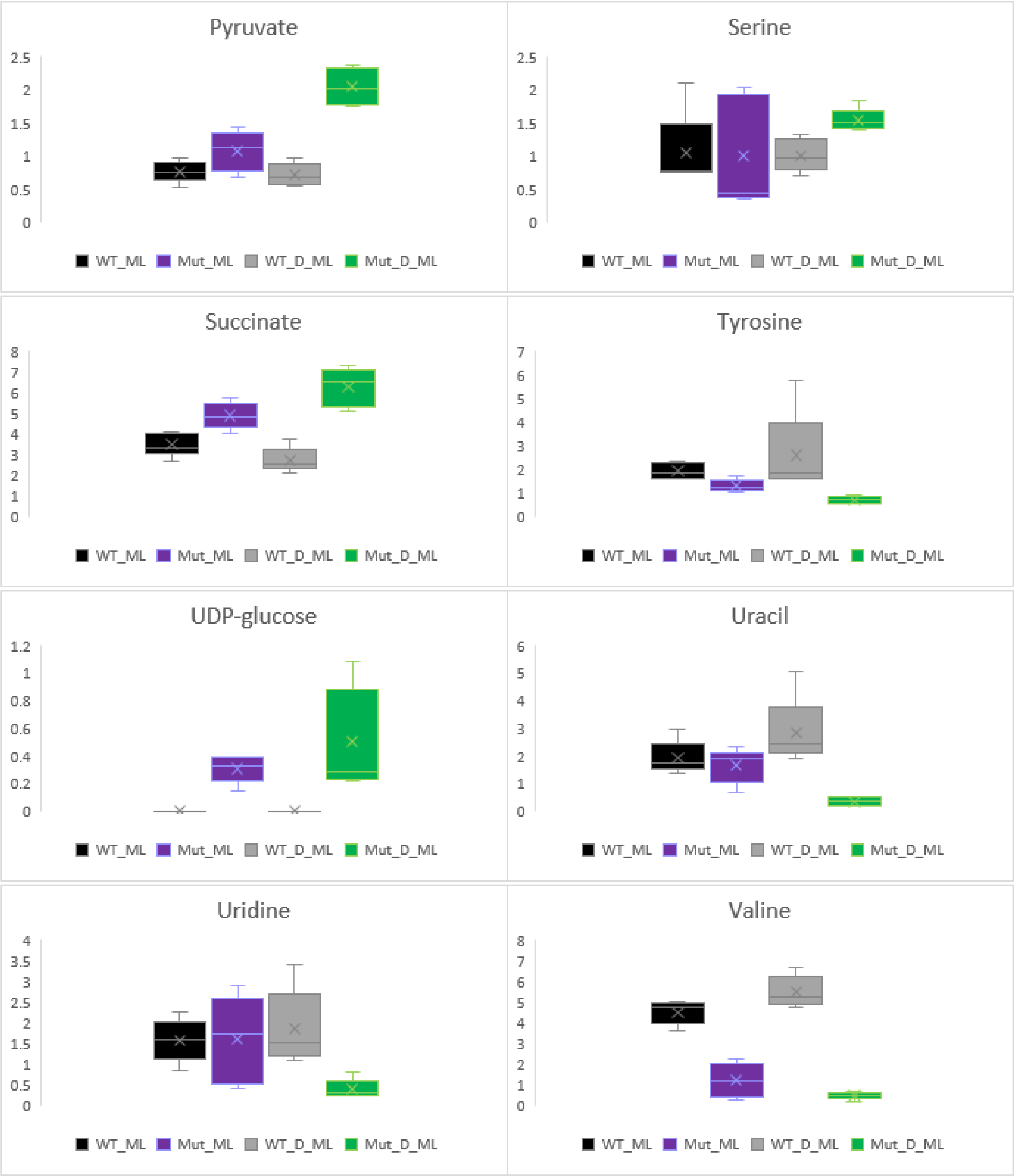

Figure S37. Mid Log Phase Metabolite Box and Whisker Plots. The ends of the whiskers are the minimum and maximum values, the center line is the median, the x is the mean, and the colored middle "box" encompasses the middle 50% of scores for the group.

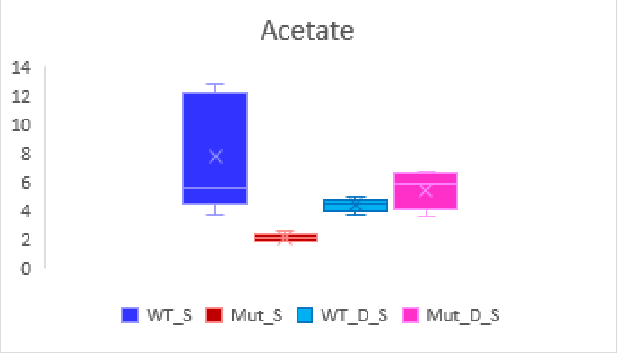
Acetate
14
12
10
8
6
4
2
0
WT_S
Mut_S
WT_D_S
Mut_D_S

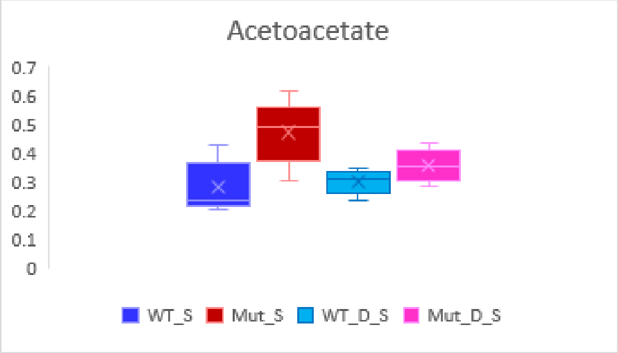
Acetoacetate
0.7
0.6
0.5
0.4
0.3
0.2
0.1
0
WT_S
Mut_S
WT_D_S
Mut_D_S

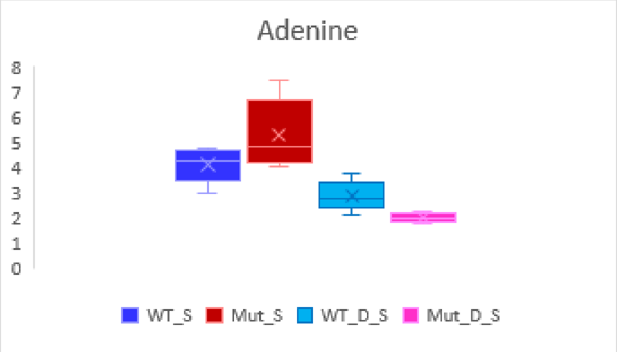
Adenine
8
7
6
5
4
3
2
1
0
WT_S
Mut_S
WT_D_S
Mut_D_S

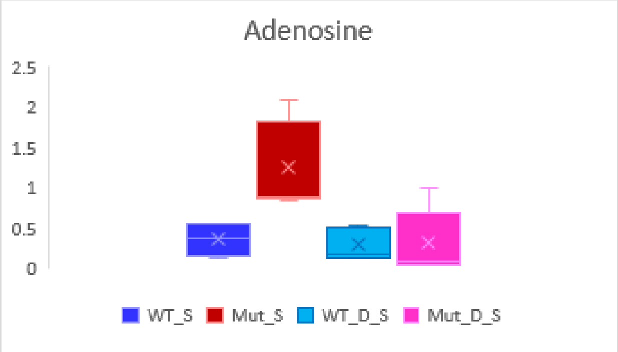
Adenosine
2.5
2
1.5
1
0.5
0
WT_S
Mut_S
WT_D_S
Mut_D_S

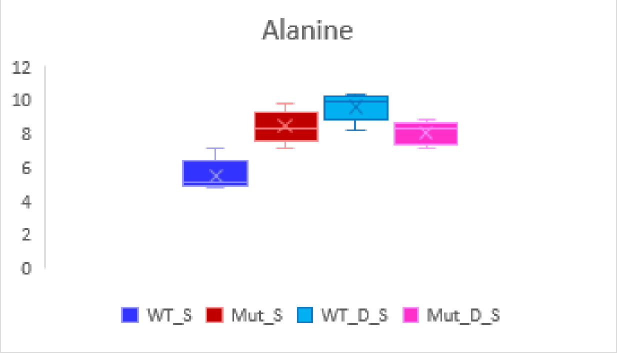
Alanine
12
10
8
6
4
2
0
WT_S
Mut_S
WT_D_S
Mut_D_S

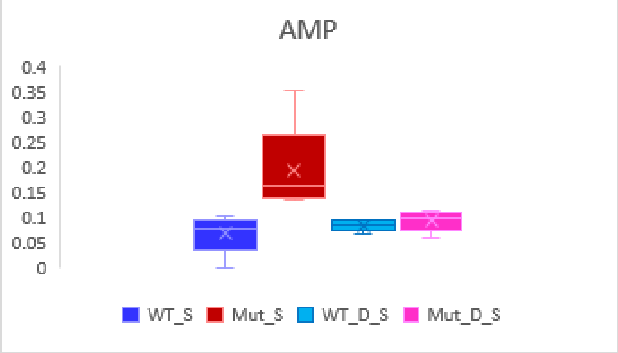
AMP
0.4
0.35
0.3
0.25
0.2
0.15
0.1
0.05
0
WT_S
Mut_S
WT_D_S
Mut_D_S

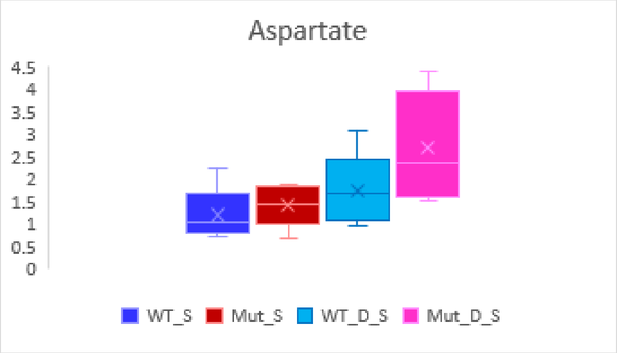
Aspartate
4.5
4
3.5
3
2.5
2
1.5
1
0.5
0
WT_S
Mut_S
WT_D_S
Mut_D_S

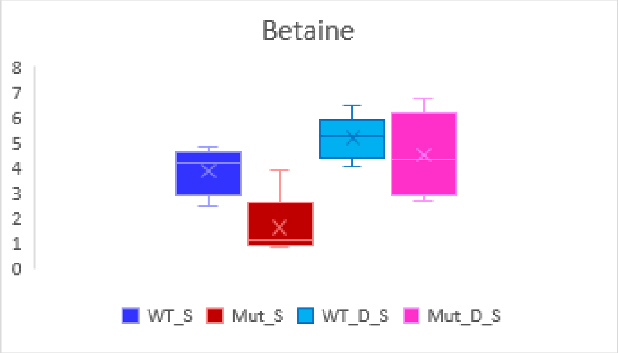
Betaine
8
7
6
5
4
3
2
1
0
WT_S
Mut_S
WT_D_S
Mut_D_S

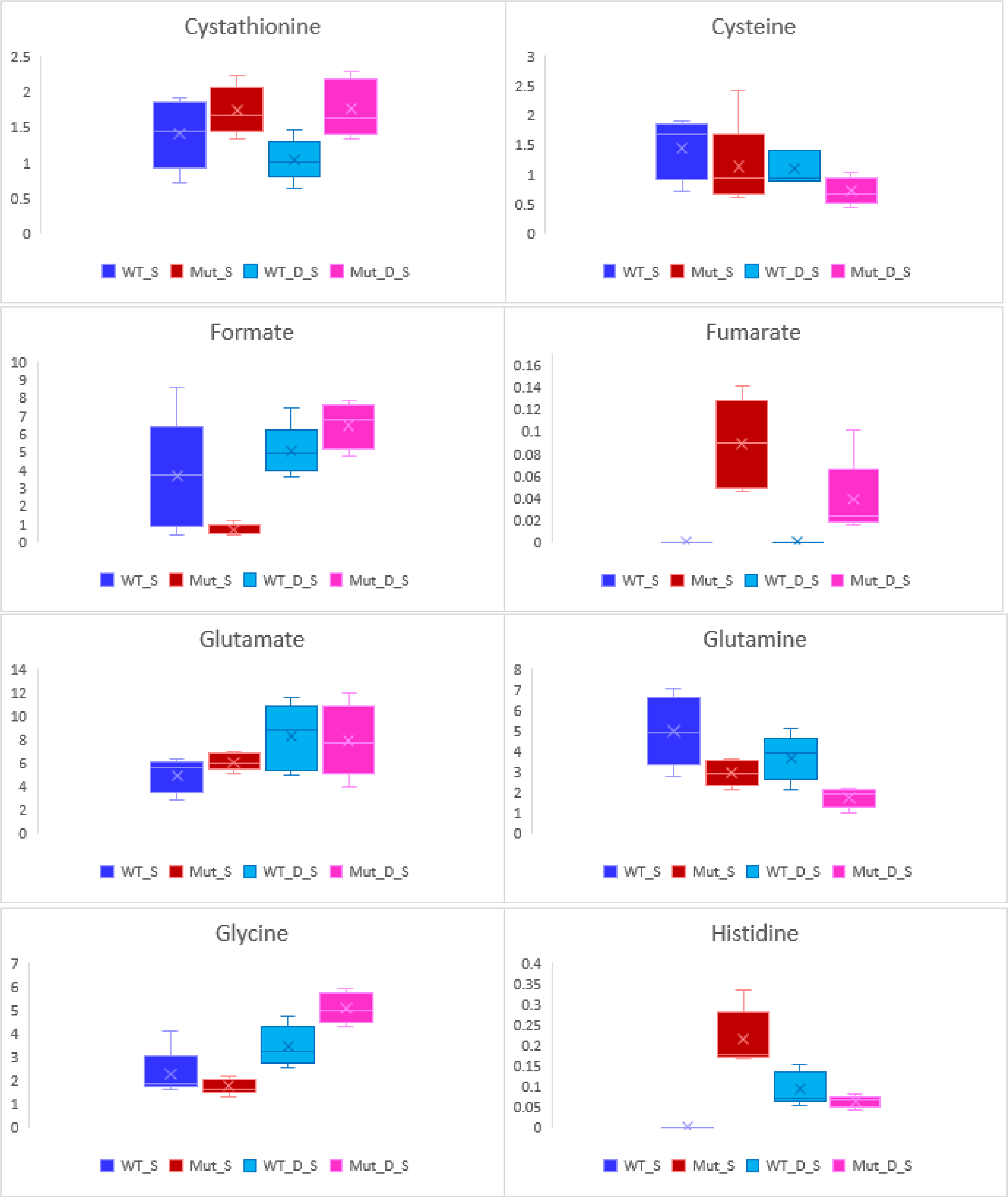
Cystathionine
2.5
2
1.5
1
0.5
0
WT_S Mut_S WT_D_S Mut_D_S
Cysteine
3
2.5
2
1.5
1
0.5
0
WT_S Mut_S WT_D_S Mut_D_S
Formate
10
9
8
7
6
5
4
3
2
1
0
WT_S Mut_S WT_D_S Mut_D_S
Fumarate
0.16
0.14
0.12
0.1
0.08
0.06
0.04
0.02
0
WT_S Mut_S WT_D_S Mut_D_S
Glutamate
14
12
10
8
6
4
2
0
WT_S Mut_S WT_D_S Mut_D_S
Glutamine
8
7
6
5
4
3
2
1
0
WT_S Mut_S WT_D_S Mut_D_S
Glycine
7
6
5
4
3
2
1
0
WT_S Mut_S WT_D_S Mut_D_S
Histidine
0.4
0.35
0.3
0.25
0.2
0.15
0.1
0.05
0
WT_S Mut_S WT_D_S Mut_D_S

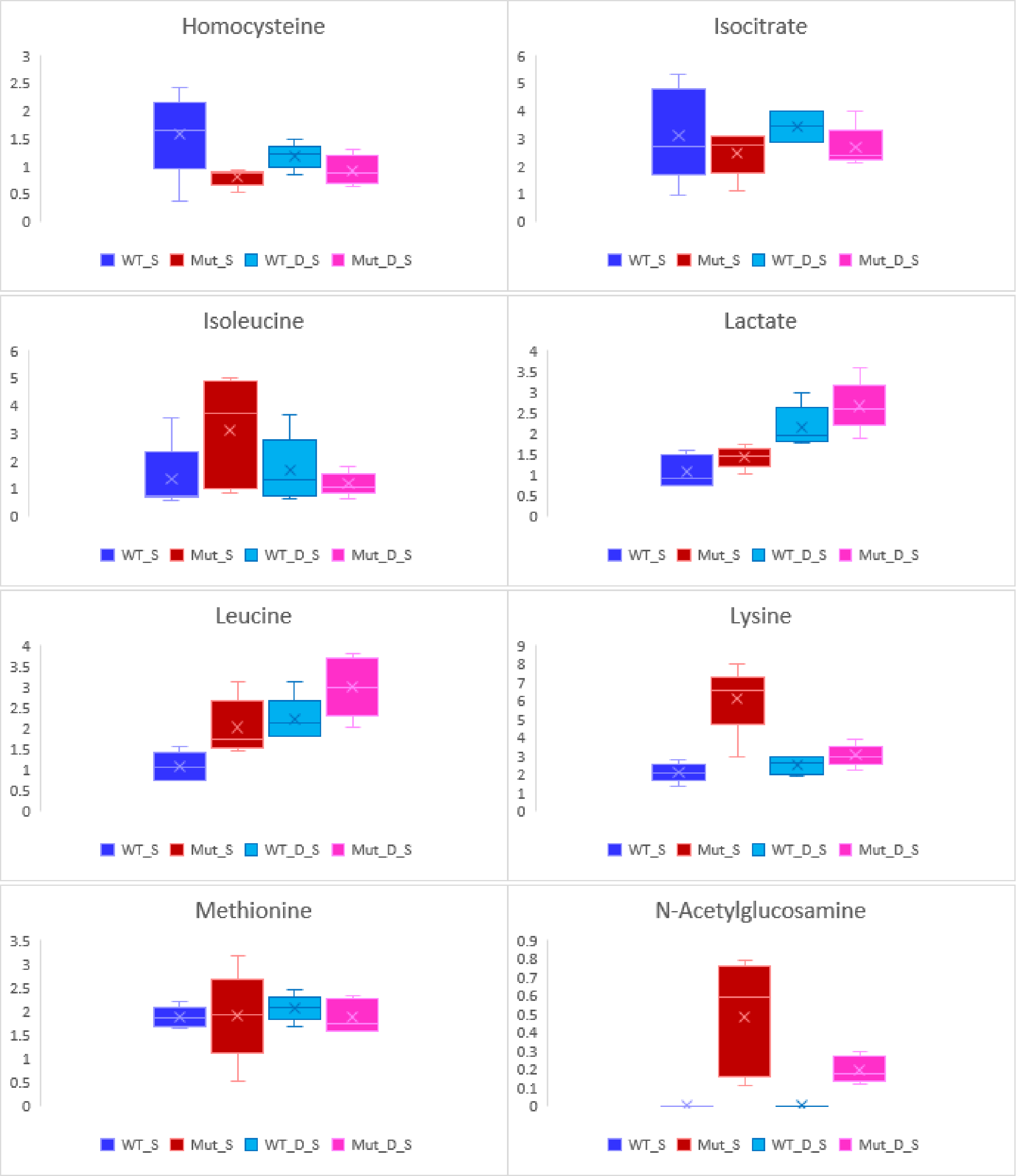
Homocysteine
3
2.5
2
1.5
1
0.5
0
WT_S Mut_S WT_D_S Mut_D_S
Isocitrate
6
5
4
3
2
1
0
WT_S Mut_S WT_D_S Mut_D_S
Isoleucine
6
5
4
3
2
1
0
WT_S Mut_S WT_D_S Mut_D_S
Lactate
4
3.5
3
2.5
2
1.5
1
0.5
0
WT_S Mut_S WT_D_S Mut_D_S
Leucine
4
3.5
3
2.5
2
1.5
1
0.5
0
WT_S Mut_S WT_D_S Mut_D_S
Lysine
9
8
7
6
5
4
3
2
1
0
WT_S Mut_S WT_D_S Mut_D_S
Methionine
3.5
3
2.5
2
1.5
1
0.5
0
WT_S Mut_S WT_D_S Mut_D_S
N-Acetylglucosamine
0.9
0.8
0.7
0.6
0.5
0.4
0.3
0.2
0.1
0
WT_S Mut_S WT_D_S Mut_D_S

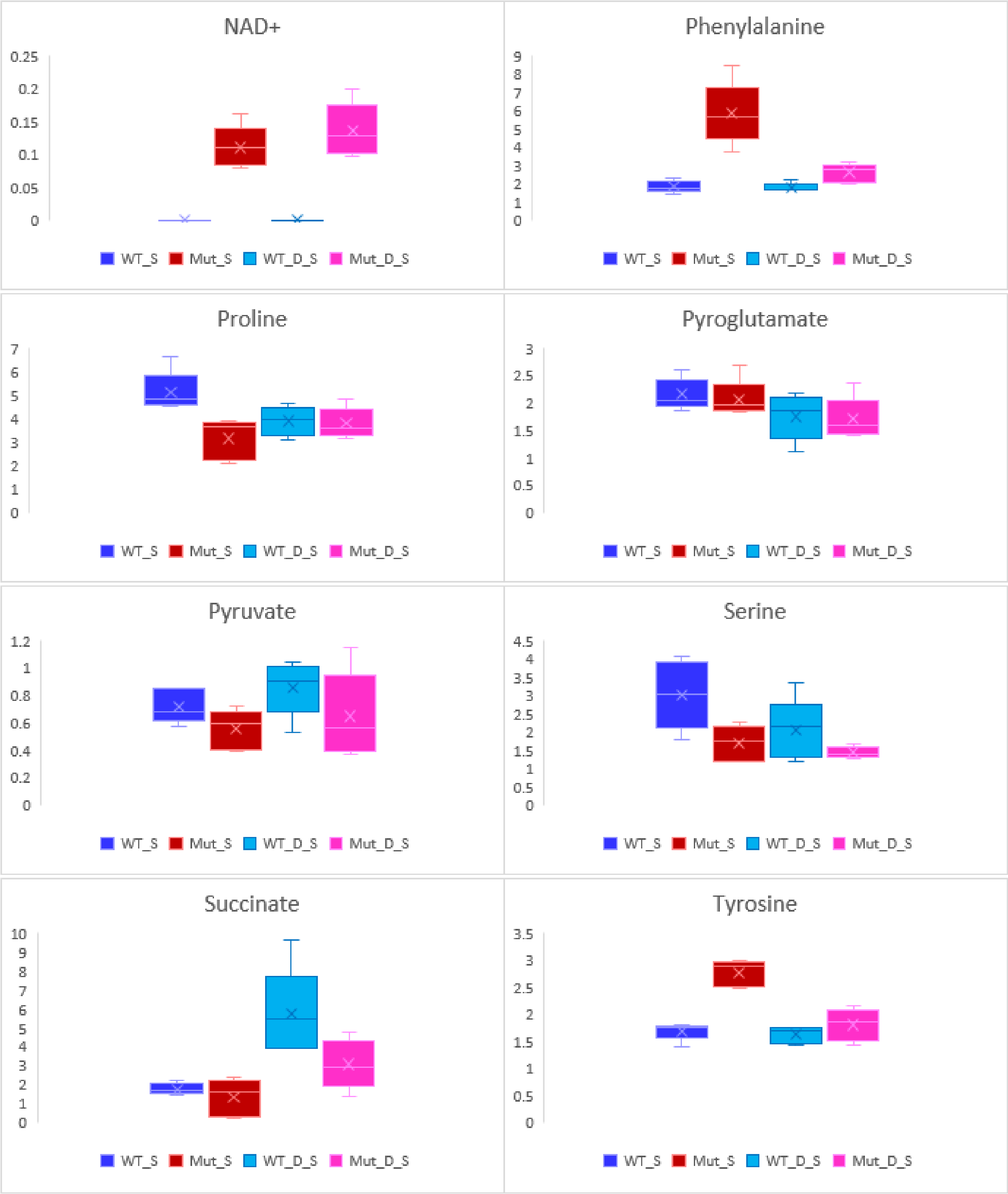
NAD+
0.25
0.2
0.15
0.1
0.05
0
WT_S Mut_S WT_D_S Mut_D_S
Phenylalanine
9
8
7
6
5
4
3
2
1
0
WT_S Mut_S WT_D_S Mut_D_S
Proline
7
6
5
4
3
2
1
0
WT_S Mut_S WT_D_S Mut_D_S
Pyroglutamate
3
2.5
2
1.5
1
0.5
0
WT_S Mut_S WT_D_S Mut_D_S
Pyruvate
1.2
1
0.8
0.6
0.4
0.2
0
WT_S Mut_S WT_D_S Mut_D_S
Serine
4.5
4
3.5
3
2.5
2
1.5
1
0.5
0
WT_S Mut_S WT_D_S Mut_D_S
Succinate
10
9
8
7
6
5
4
3
2
1
0
WT_S Mut_S WT_D_S Mut_D_S
Tyrosine
3.5
3
2.5
2
1.5
1
0.5
0
WT_S Mut_S WT_D_S Mut_D_S

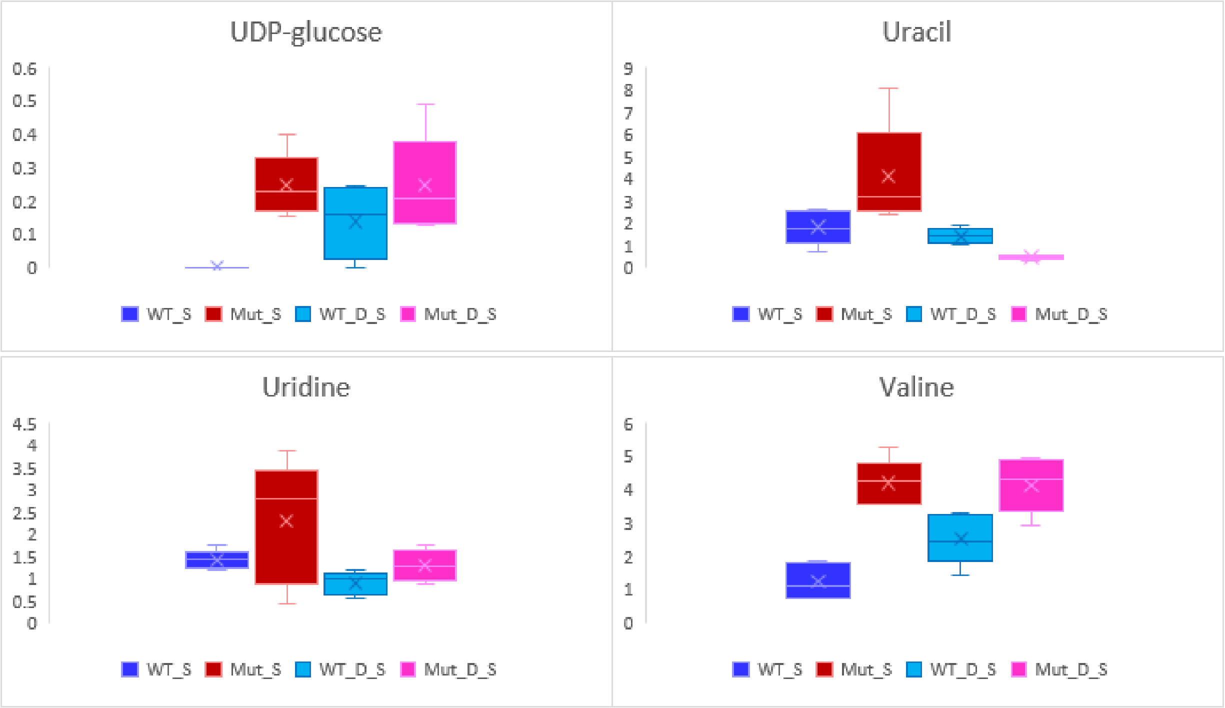

Figure S38. Stationary Phase Metabolite Box and Whisker Plots. The ends of the whiskers are the minimum and maximum values, the center line is the median, the x is the mean, and the colored middle "box" encompasses the middle 50% of scores for the group.

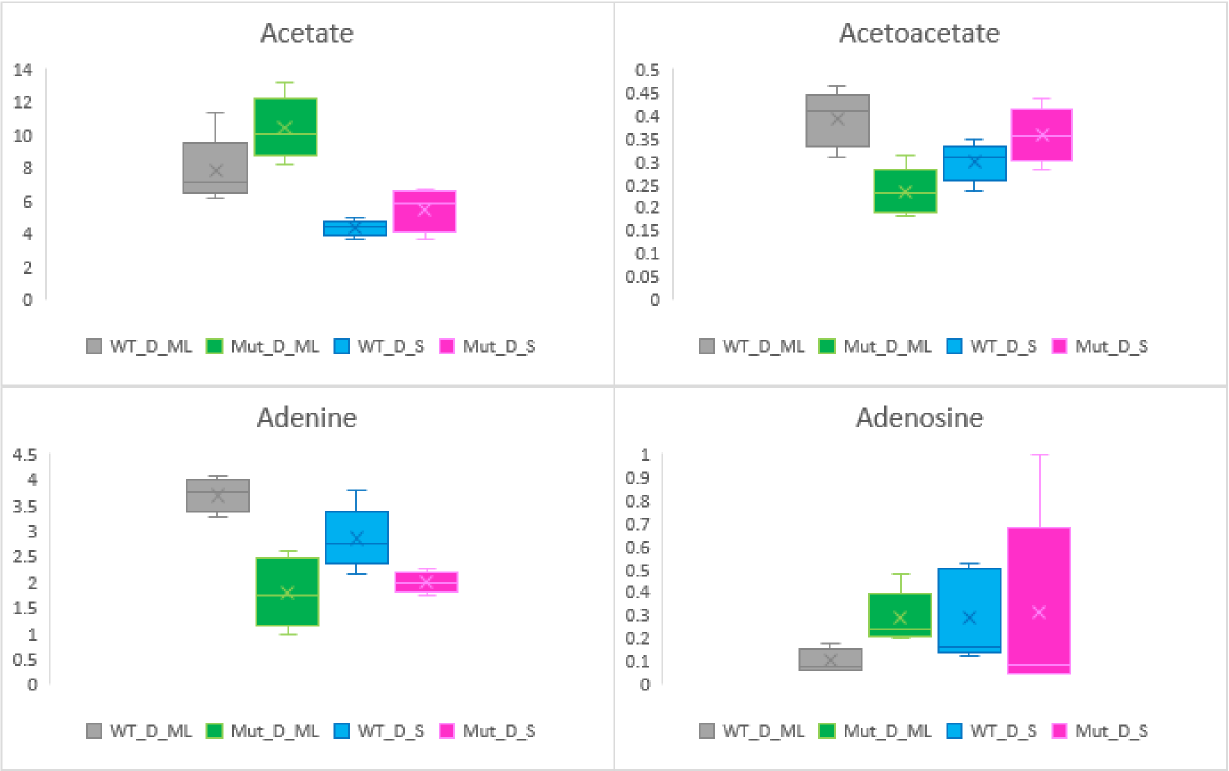

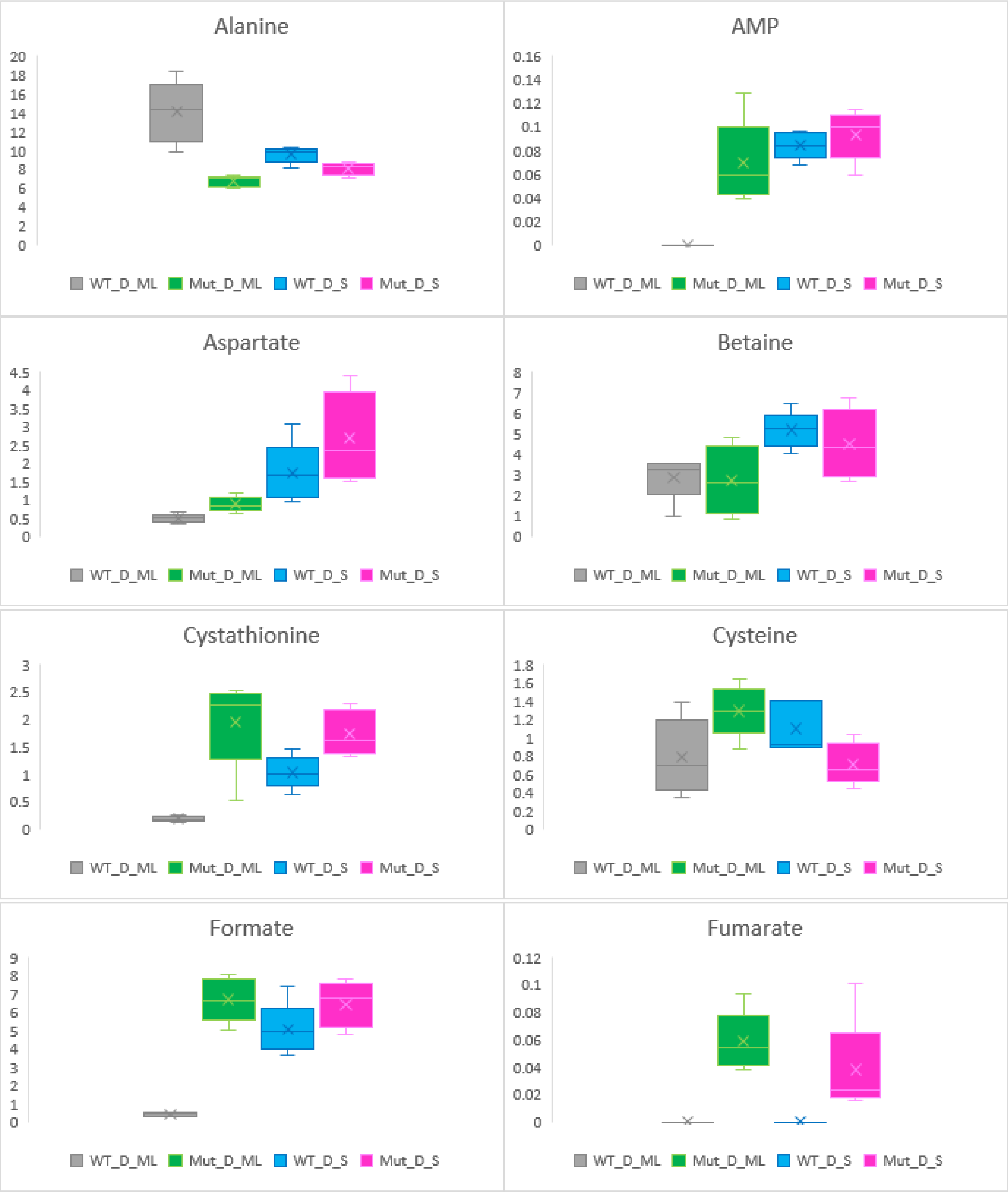
Alanine
20
18
16
14
12
10
8
6
4
2
0
WT_D_ML
Mut_D_ML
WT_D_S
Mut_D_S
AMP
0.16
0.14
0.12
0.1
0.08
0.06
0.04
0.02
0
WT_D_ML
Mut_D_ML
WT_D_S
Mut_D_S
Aspartate
4.5
4
3.5
3
2.5
2
1.5
1
0.5
0
WT_D_ML
Mut_D_ML
WT_D_S
Mut_D_S
Betaine
8
7
6
5
4
3
2
1
0
WT_D_ML
Mut_D_ML
WT_D_S
Mut_D_S
Cystathionine
3
2.5
2
1.5
1
0.5
0
WT_D_ML
Mut_D_ML
WT_D_S
Mut_D_S
Cysteine
1.8
1.6
1.4
1.2
1
0.8
0.6
0.4
0.2
0
WT_D_ML
Mut_D_ML
WT_D_S
Mut_D_S
Formate
9
8
7
6
5
4
3
2
1
0
WT_D_ML
Mut_D_ML
WT_D_S
Mut_D_S
Fumarate
0.12
0.1
0.08
0.06
0.04
0.02
0
WT_D_ML
Mut_D_ML
WT_D_S
Mut_D_S

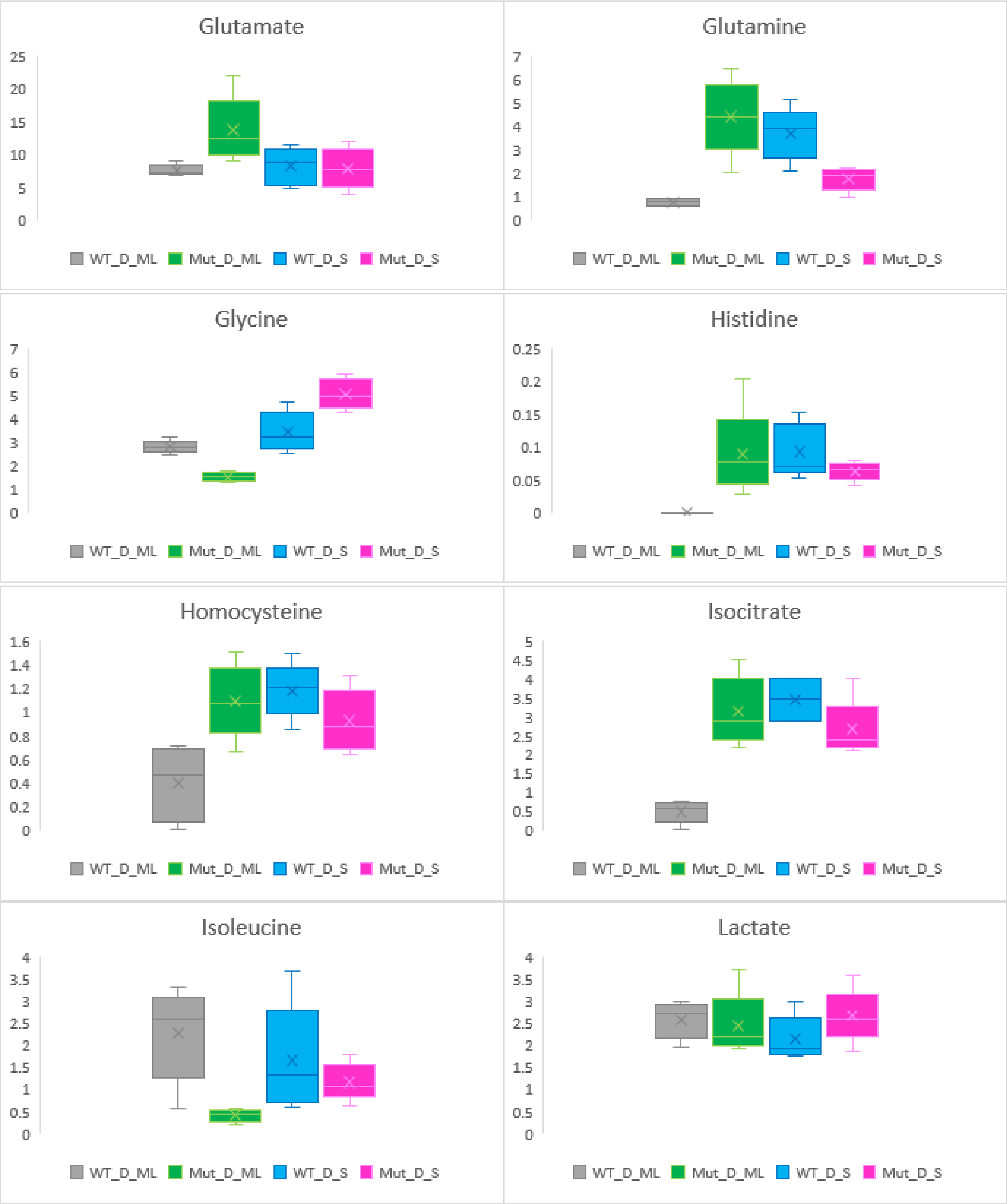
Glutamate
25
20
15
10
5
0
WT_D_ML
Mut_D_ML
WT_D_S
Mut_D_S
Glutamine
7
6
5
4
3
2
1
0
WT_D_ML
Mut_D_ML
WT_D_S
Mut_D_S
Glycine
7
6
5
4
3
2
1
0
WT_D_ML
Mut_D_ML
WT_D_S
Mut_D_S
Histidine
0.25
0.2
0.15
0.1
0.05
0
WT_D_ML
Mut_D_ML
WT_D_S
Mut_D_S
Homocysteine
1.6
1.4
1.2
1
0.8
0.6
0.4
0.2
0
WT_D_ML
Mut_D_ML
WT_D_S
Mut_D_S
Isocitrate
5
4.5
4
3.5
3
2.5
2
1.5
1
0.5
0
WT_D_ML
Mut_D_ML
WT_D_S
Mut_D_S
Isoleucine
4
3.5
3
2.5
2
1.5
1
0.5
0
WT_D_ML
Mut_D_ML
WT_D_S
Mut_D_S
Lactate
4
3.5
3
2.5
2
1.5
1
0.5
0
WT_D_ML
Mut_D_ML
WT_D_S
Mut_D_S

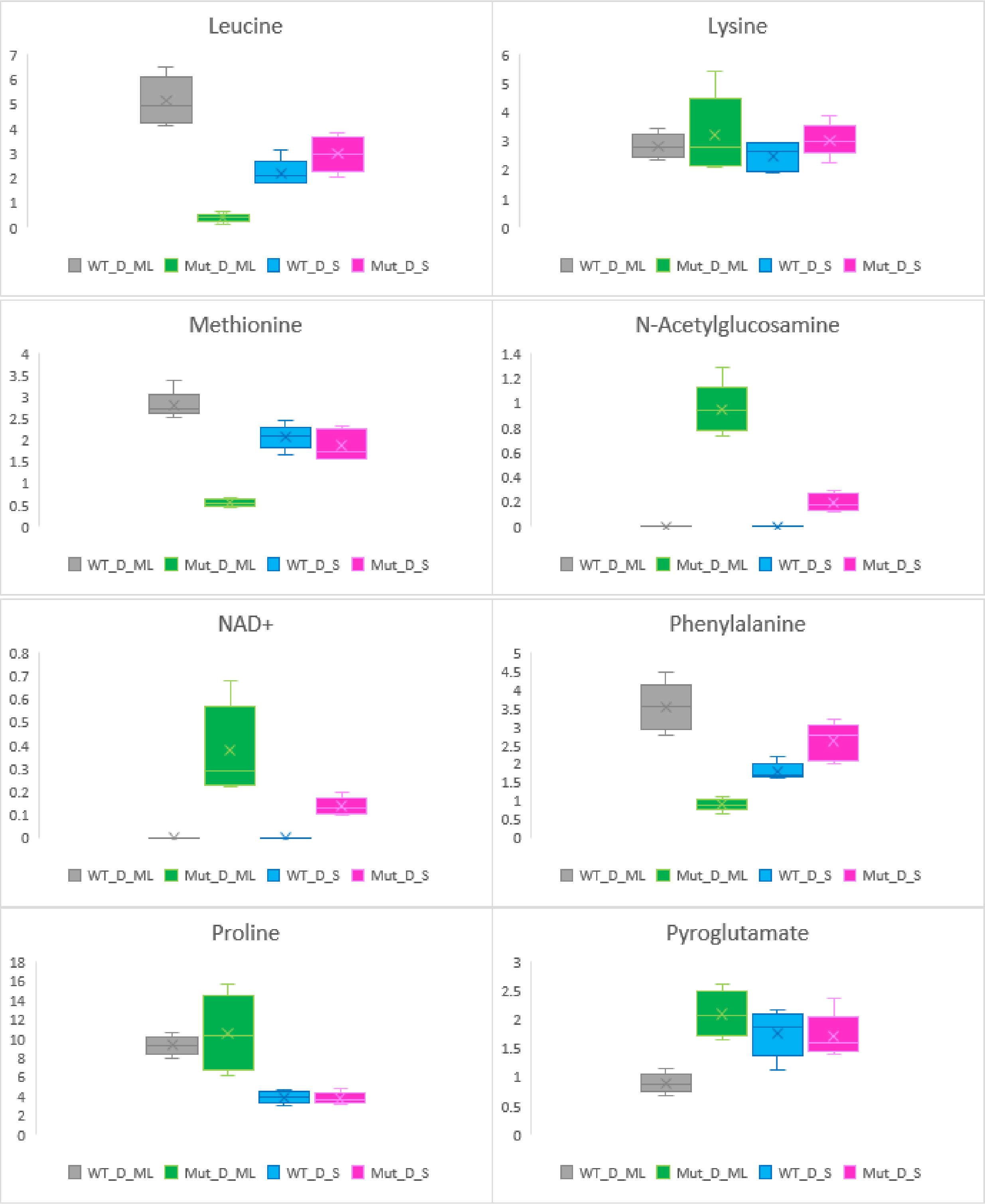
Leucine
7
6
5
4
3
2
1
0
WT_D_ML
Mut_D_ML
WT_D_S
Mut_D_S
Lysine
6
5
4
3
2
1
0
WT_D_ML
Mut_D_ML
WT_D_S
Mut_D_S
Methionine
4
3.5
3
2.5
2
1.5
1
0.5
0
WT_D_ML
Mut_D_ML
WT_D_S
Mut_D_S
N-Acetylglucosamine
1.4
1.2
1
0.8
0.6
0.4
0.2
0
WT_D_ML
Mut_D_ML
WT_D_S
Mut_D_S
NAD+
0.8
0.7
0.6
0.5
0.4
0.3
0.2
0.1
0
WT_D_ML
Mut_D_ML
WT_D_S
Mut_D_S
Phenylalanine
5
4.5
4
3.5
3
2.5
2
1.5
1
0.5
0
WT_D_ML
Mut_D_ML
WT_D_S
Mut_D_S
Proline
18
16
14
12
10
8
6
4
2
0
WT_D_ML
Mut_D_ML
WT_D_S
Mut_D_S
Pyroglutamate
3
2.5
2
1.5
1
0.5
0
WT_D_ML
Mut_D_ML
WT_D_S
Mut_D_S

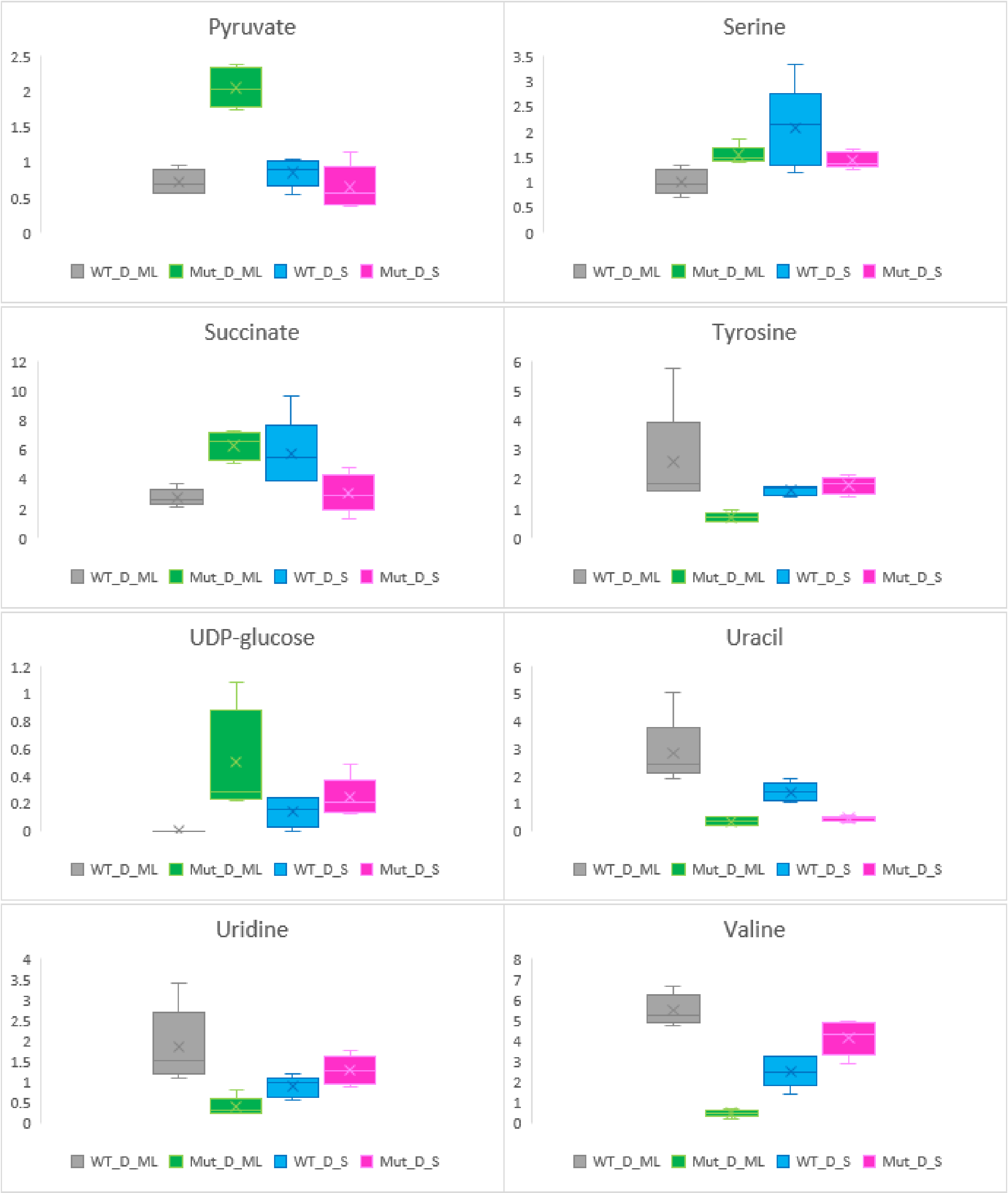

Figure S39. Challenged Sample Types Metabolite Box and Whisker Plots. The ends of the whiskers are the minimum and maximum values, the center line is the median, the x is the mean, and the colored middle “box” encompasses the middle 50% of scores for the group.

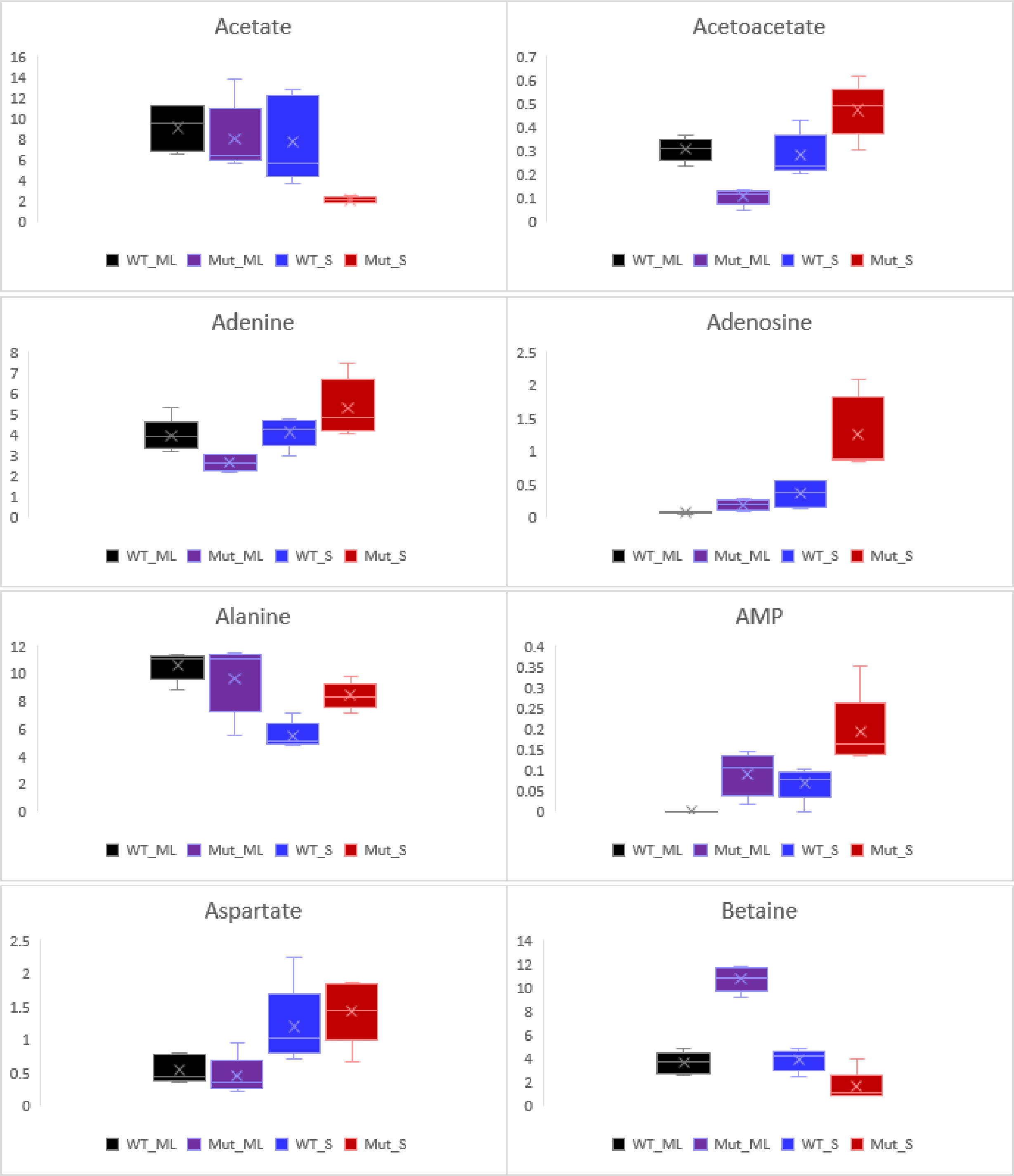
Acetate
16
14
12
10
8
6
4
2
0
WT_ML Mut_ML WT_S Mut_S
Acetoacetate
0.7
0.6
0.5
0.4
0.3
0.2
0.1
0
WT_ML Mut_ML WT_S Mut_S
Adenine
8
7
6
5
4
3
2
1
0
WT_ML Mut_ML WT_S Mut_S
Adenosine
2.5
2
1.5
1
0.5
0
WT_ML Mut_ML WT_S Mut_S
Alanine
12
10
8
6
4
2
0
WT_ML Mut_ML WT_S Mut_S
AMP
0.4
0.35
0.3
0.25
0.2
0.15
0.1
0.05
0
WT_ML Mut_ML WT_S Mut_S
Aspartate
2.5
2
1.5
1
0.5
0
WT_ML Mut_ML WT_S Mut_S
Betaine
14
12
10
8
6
4
2
0
WT_ML Mut_ML WT_S Mut_S

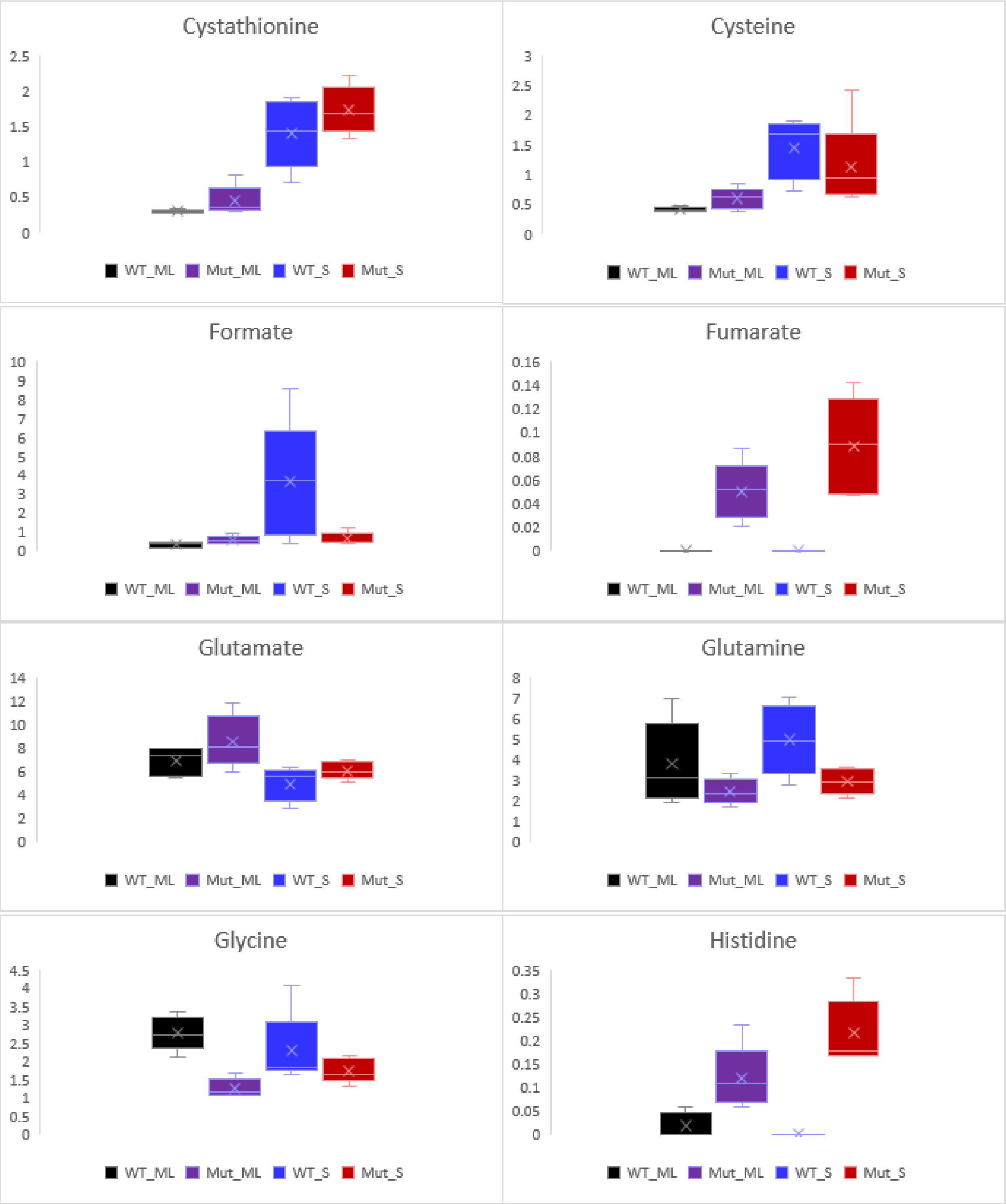
Cystathionine
2.5
2
1.5
1
0.5
0
WT_ML Mut_ML WT_S Mut_S
Cysteine
3
2.5
2
1.5
1
0.5
0
WT_ML Mut_ML WT_S Mut_S
Formate
10
9
8
7
6
5
4
3
2
1
0
WT_ML Mut_ML WT_S Mut_S
Fumarate
0.16
0.14
0.12
0.1
0.08
0.06
0.04
0.02
0
WT_ML Mut_ML WT_S Mut_S
Glutamate
14
12
10
8
6
4
2
0
WT_ML Mut_ML WT_S Mut_S
Glutamine
8
7
6
5
4
3
2
1
0
WT_ML Mut_ML WT_S Mut_S
Glycine
4.5
4
3.5
3
2.5
2
1.5
1
0.5
0
WT_ML Mut_ML WT_S Mut_S
Histidine
0.35
0.3
0.25
0.2
0.15
0.1
0.05
0
WT_ML Mut_ML WT_S Mut_S

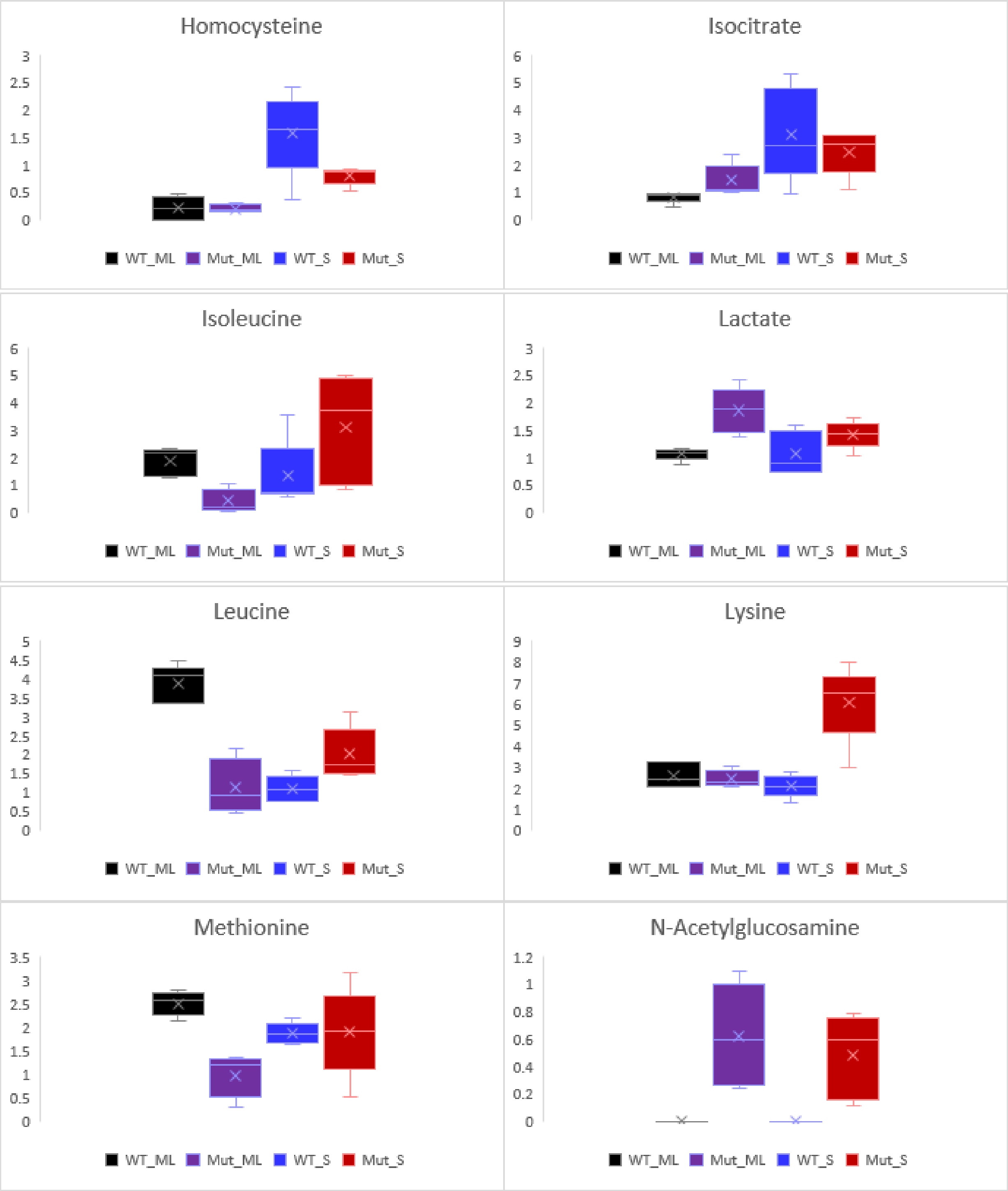
Homocysteine
3
2.5
2
1.5
1
0.5
0
WT_ML Mut_ML WT_S Mut_S
Isocitrate
6
5
4
3
2
1
0
WT_ML Mut_ML WT_S Mut_S
Isoleucine
6
5
4
3
2
1
0
WT_ML Mut_ML WT_S Mut_S
Lactate
3
2.5
2
1.5
1
0.5
0
WT_ML Mut_ML WT_S Mut_S
Leucine
5
4.5
4
3.5
3
2.5
2
1.5
1
0.5
0
WT_ML Mut_ML WT_S Mut_S
Lysine
9
8
7
6
5
4
3
2
1
0
WT_ML Mut_ML WT_S Mut_S
Methionine
3.5
3
2.5
2
1.5
1
0.5
0
WT_ML Mut_ML WT_S Mut_S
N-Acetylglucosamine
1.2
1
0.8
0.6
0.4
0.2
0
WT_ML Mut_ML WT_S Mut_S

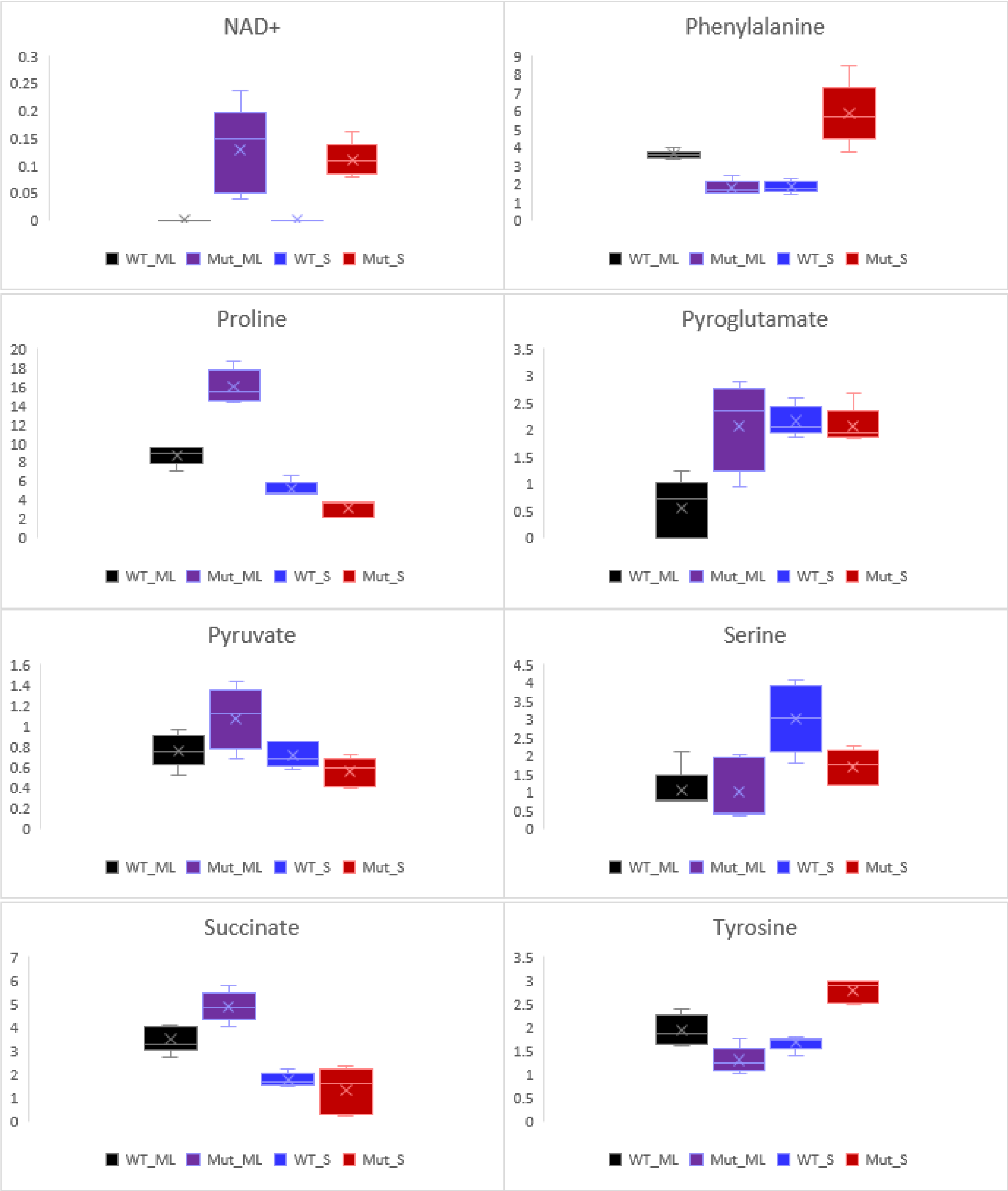
NAD+
0.3
0.25
0.2
0.15
0.1
0.05
0
WT_ML Mut_ML WT_S Mut_S
Phenylalanine
9
8
7
6
5
4
3
2
1
0
WT_ML Mut_ML WT_S Mut_S
Proline
20
18
16
14
12
10
8
6
4
2
0
WT_ML Mut_ML WT_S Mut_S
Pyroglutamate
3.5
3
2.5
2
1.5
1
0.5
0
WT_ML Mut_ML WT_S Mut_S
Pyruvate
1.6
1.4
1.2
1
0.8
0.6
0.4
0.2
0
WT_ML Mut_ML WT_S Mut_S
Serine
4.5
4
3.5
3
2.5
2
1.5
1
0.5
0
WT_ML Mut_ML WT_S Mut_S
Succinate
7
6
5
4
3
2
1
0
WT_ML Mut_ML WT_S Mut_S
Tyrosine
3.5
3
2.5
2
1.5
1
0.5
0
WT_ML Mut_ML WT_S Mut_S

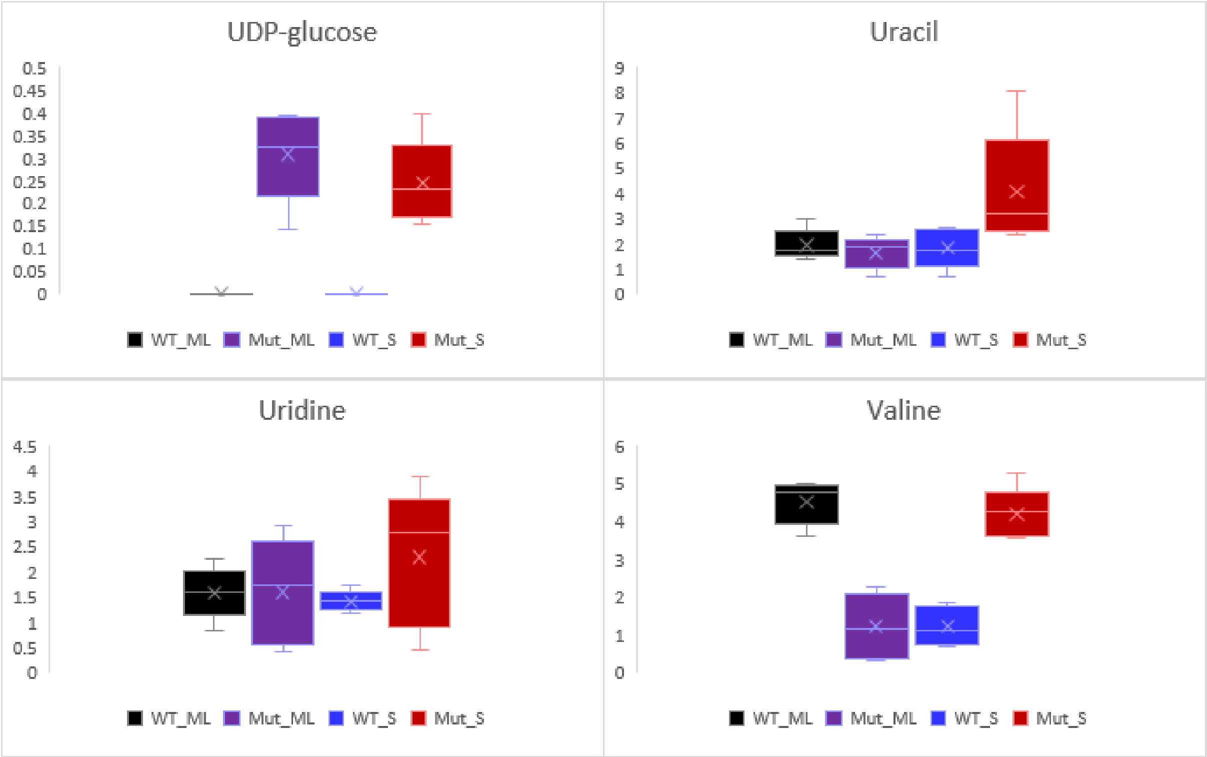

Figure S40. Unchallenged Sample Types Metabolite Box and Whisker Plots. The ends of the whiskers are the minimum and maximum values, the center line is the median, the x is the mean, and the colored middle "box" encompasses the middle 50% of scores for the group.

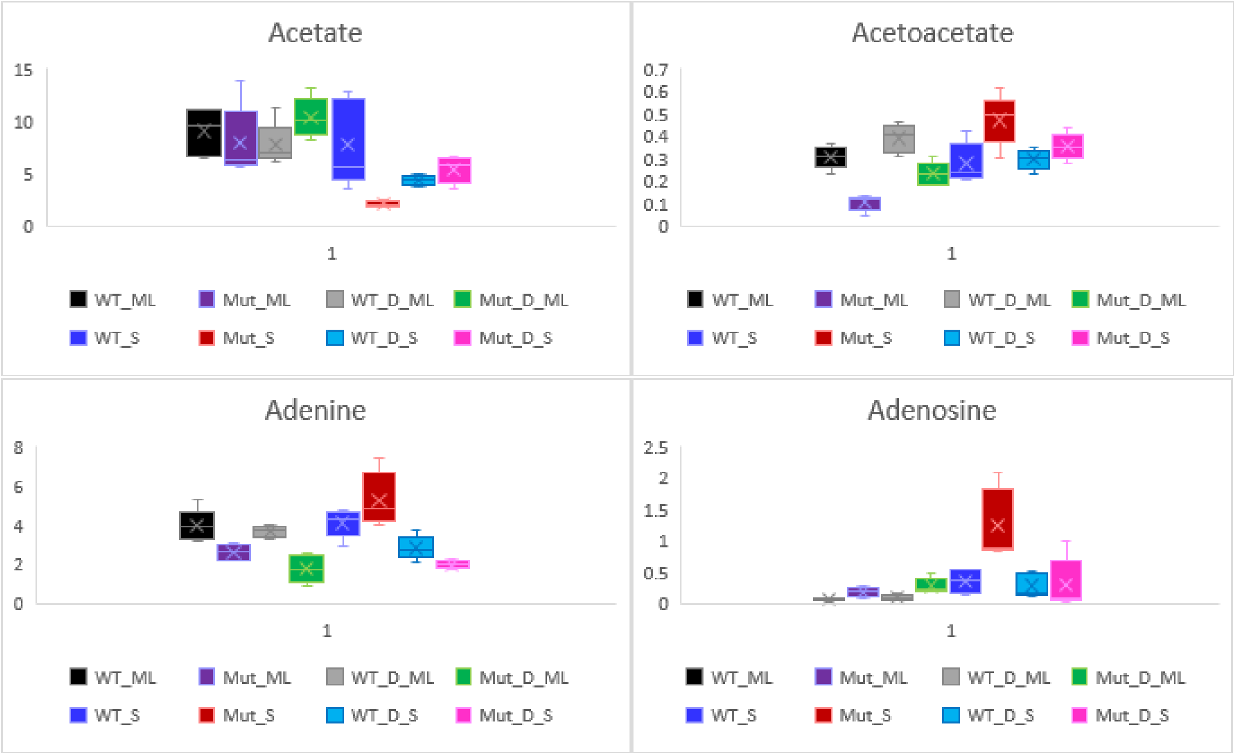

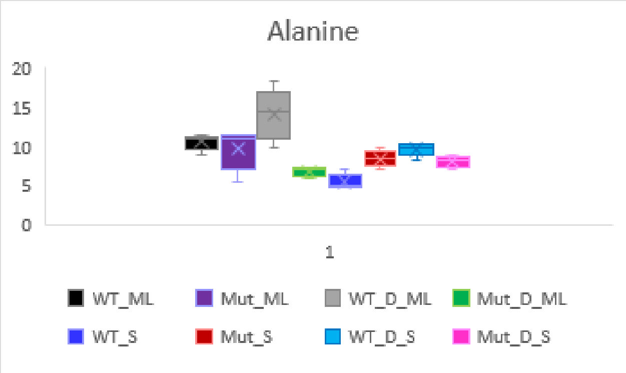
Alanine
20
15
10
5
0
1
WT_ML
Mut_ML
WT_D_ML
Mut_D_ML
WT_S
Mut_S
WT_D_S
Mut_D_S

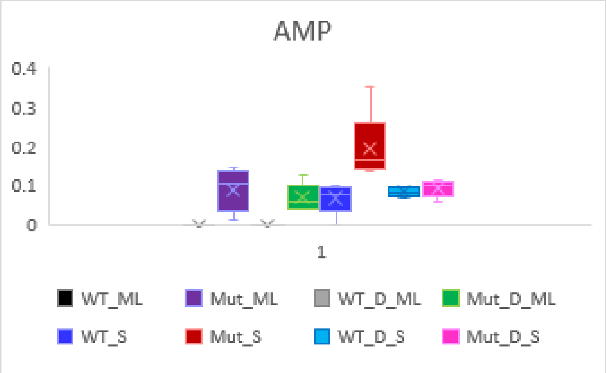
AMP
0.4
0.3
0.2
0.1
0
1
WT_ML
Mut_ML
WT_D_ML
Mut_D_ML
WT_S
Mut_S
WT_D_S
Mut_D_S

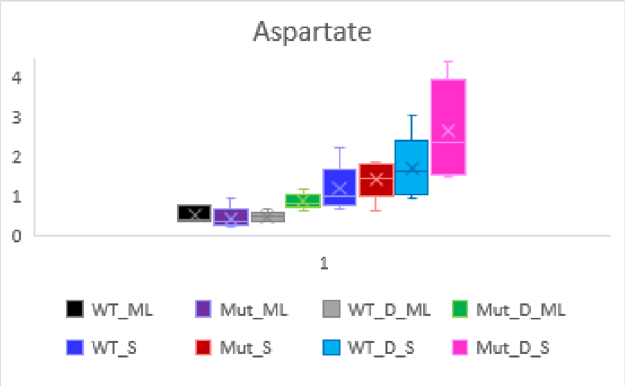
Aspartate
4
3
2
1
0
1
WT_ML
Mut_ML
WT_D_ML
Mut_D_ML
WT_S
Mut_S
WT_D_S
Mut_D_S

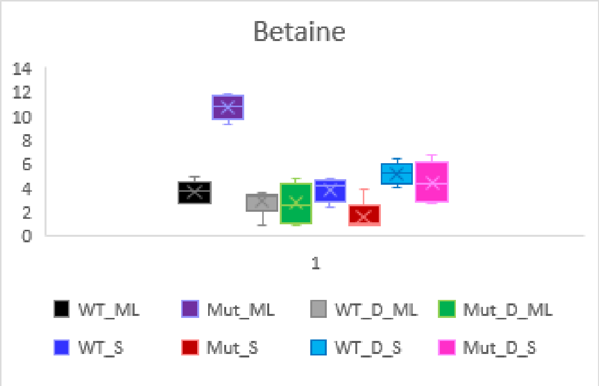
Betaine
14
12
10
8
6
4
2
0
1
WT_ML
Mut_ML
WT_D_ML
Mut_D_ML
WT_S
Mut_S
WT_D_S
Mut_D_S

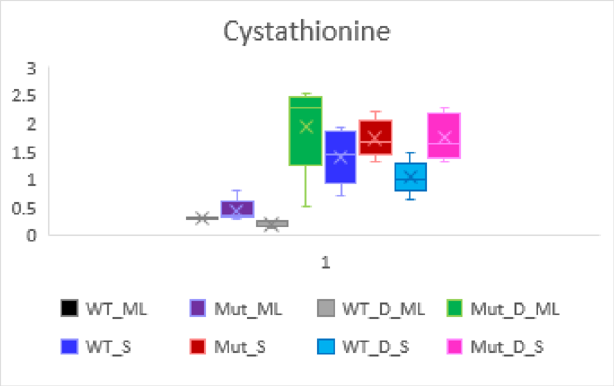
Cystathionine
3
2.5
2
1.5
1
0.5
0
1
WT_ML
Mut_ML
WT_D_ML
Mut_D_ML
WT_S
Mut_S
WT_D_S
Mut_D_S

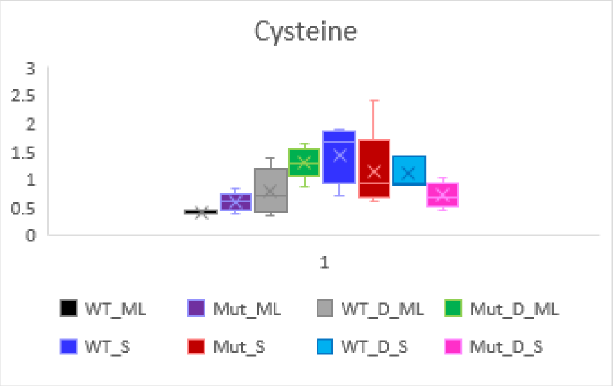
Cysteine
3
2.5
2
1.5
1
0.5
0
1
WT_ML
Mut_ML
WT_D_ML
Mut_D_ML
WT_S
Mut_S
WT_D_S
Mut_D_S

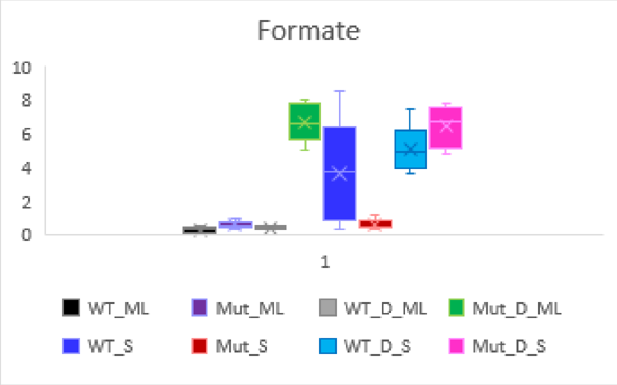
Formate
10
8
6
4
2
0
1
WT_ML
Mut_ML
WT_D_ML
Mut_D_ML
WT_S
Mut_S
WT_D_S
Mut_D_S

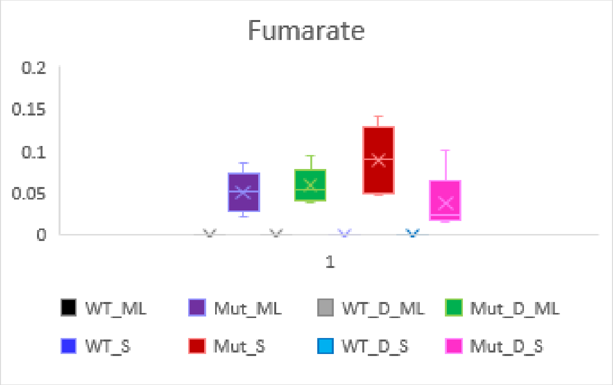
Fumarate
0.2
0.15
0.1
0.05
0
1
WT_ML
Mut_ML
WT_D_ML
Mut_D_ML
WT_S
Mut_S
WT_D_S
Mut_D_S

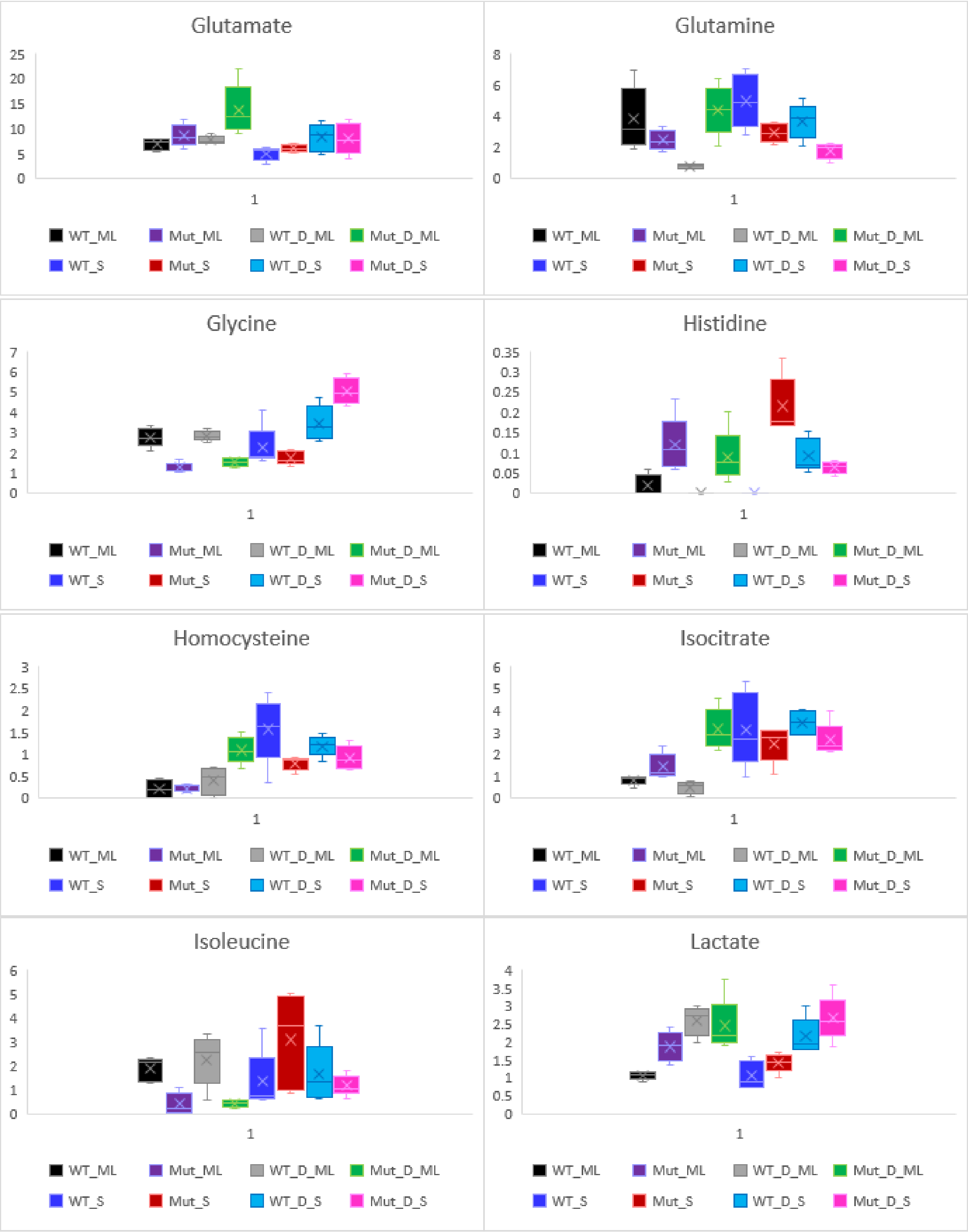
Glutamate
25
20
15
10
5
0
1
WT_ML
Mut_ML
WT_D_ML
Mut_D_ML
WT_S
Mut_S
WT_D_S
Mut_D_S
Glutamine
8
6
4
2
0
1
WT_ML
Mut_ML
WT_D_ML
Mut_D_ML
WT_S
Mut_S
WT_D_S
Mut_D_S
Glycine
7
6
5
4
3
2
1
0
1
WT_ML
Mut_ML
WT_D_ML
Mut_D_ML
WT_S
Mut_S
WT_D_S
Mut_D_S
Histidine
0.35
0.3
0.25
0.2
0.15
0.1
0.05
0
1
WT_ML
Mut_ML
WT_D_ML
Mut_D_ML
WT_S
Mut_S
WT_D_S
Mut_D_S
Homocysteine
3
2.5
2
1.5
1
0.5
0
1
WT_ML
Mut_ML
WT_D_ML
Mut_D_ML
WT_S
Mut_S
WT_D_S
Mut_D_S
Isocitrate
6
5
4
3
2
1
0
1
WT_ML
Mut_ML
WT_D_ML
Mut_D_ML
WT_S
Mut_S
WT_D_S
Mut_D_S
Isoleucine
6
5
4
3
2
1
0
1
WT_ML
Mut_ML
WT_D_ML
Mut_D_ML
WT_S
Mut_S
WT_D_S
Mut_D_S
Lactate
4
3.5
3
2.5
2
1.5
1
0.5
0
1
WT_ML
Mut_ML
WT_D_ML
Mut_D_ML
WT_S
Mut_S
WT_D_S
Mut_D_S

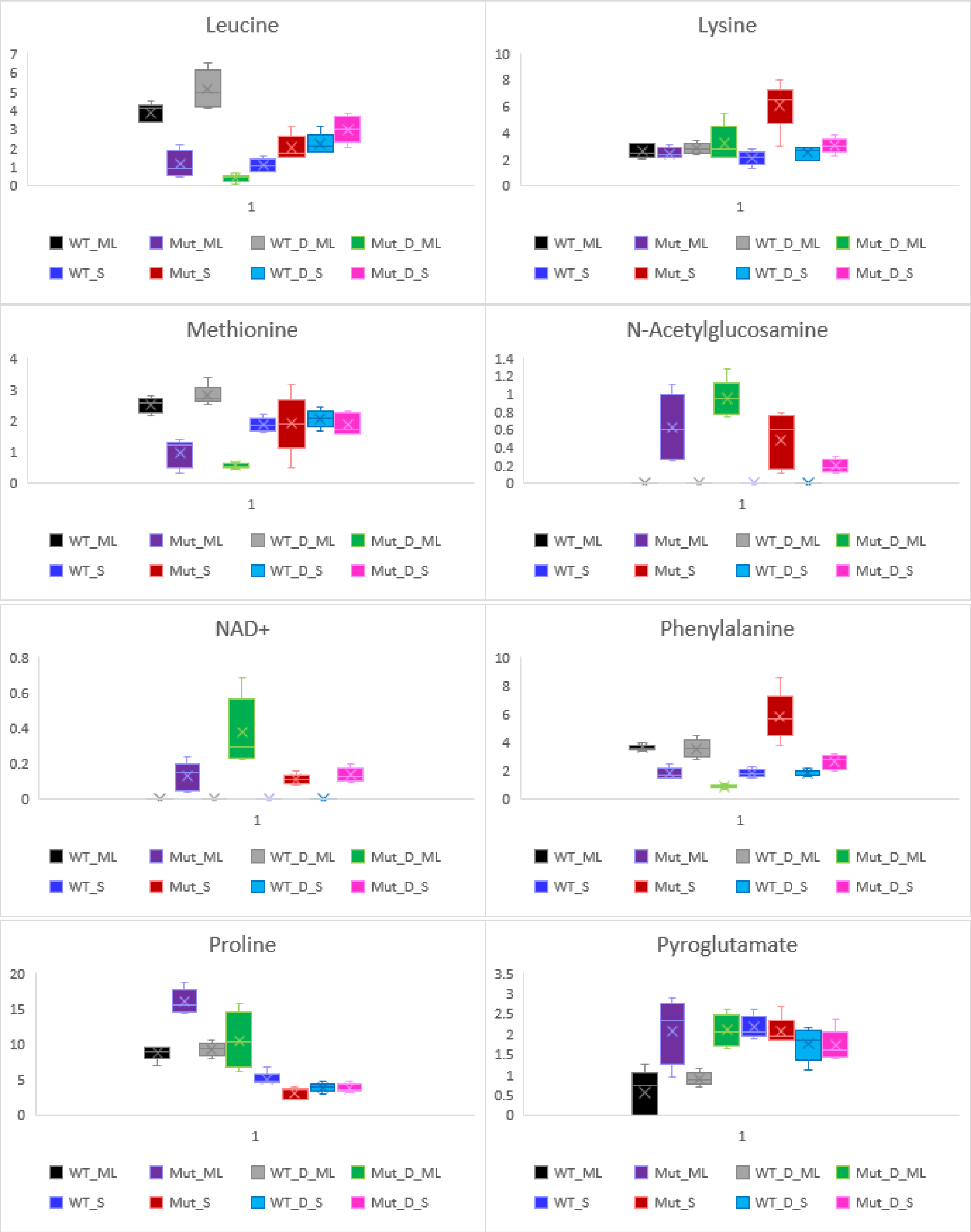
Leucine
7
6
5
4
3
2
1
0
1
WT_ML
Mut_ML
WT_D_ML
Mut_D_ML
WT_S
Mut_S
WT_D_S
Mut_D_S
Lysine
10
8
6
4
2
0
1
WT_ML
Mut_ML
WT_D_ML
Mut_D_ML
WT_S
Mut_S
WT_D_S
Mut_D_S
Methionine
4
3
2
1
0
1
WT_ML
Mut_ML
WT_D_ML
Mut_D_ML
WT_S
Mut_S
WT_D_S
Mut_D_S
N-Acetylglucosamine
1.4
1.2
1
0.8
0.6
0.4
0.2
0
1
WT_ML
Mut_ML
WT_D_ML
Mut_D_ML
WT_S
Mut_S
WT_D_S
Mut_D_S
NAD+
0.8
0.6
0.4
0.2
0
1
WT_ML
Mut_ML
WT_D_ML
Mut_D_ML
WT_S
Mut_S
WT_D_S
Mut_D_S
Phenylalanine
10
8
6
4
2
0
1
WT_ML
Mut_ML
WT_D_ML
Mut_D_ML
WT_S
Mut_S
WT_D_S
Mut_D_S
Proline
20
15
10
5
0
1
WT_ML
Mut_ML
WT_D_ML
Mut_D_ML
WT_S
Mut_S
WT_D_S
Mut_D_S
Pyroglutamate
3.5
3
2.5
2
1.5
1
0.5
0
1
WT_ML
Mut_ML
WT_D_ML
Mut_D_ML
WT_S
Mut_S
WT_D_S
Mut_D_S

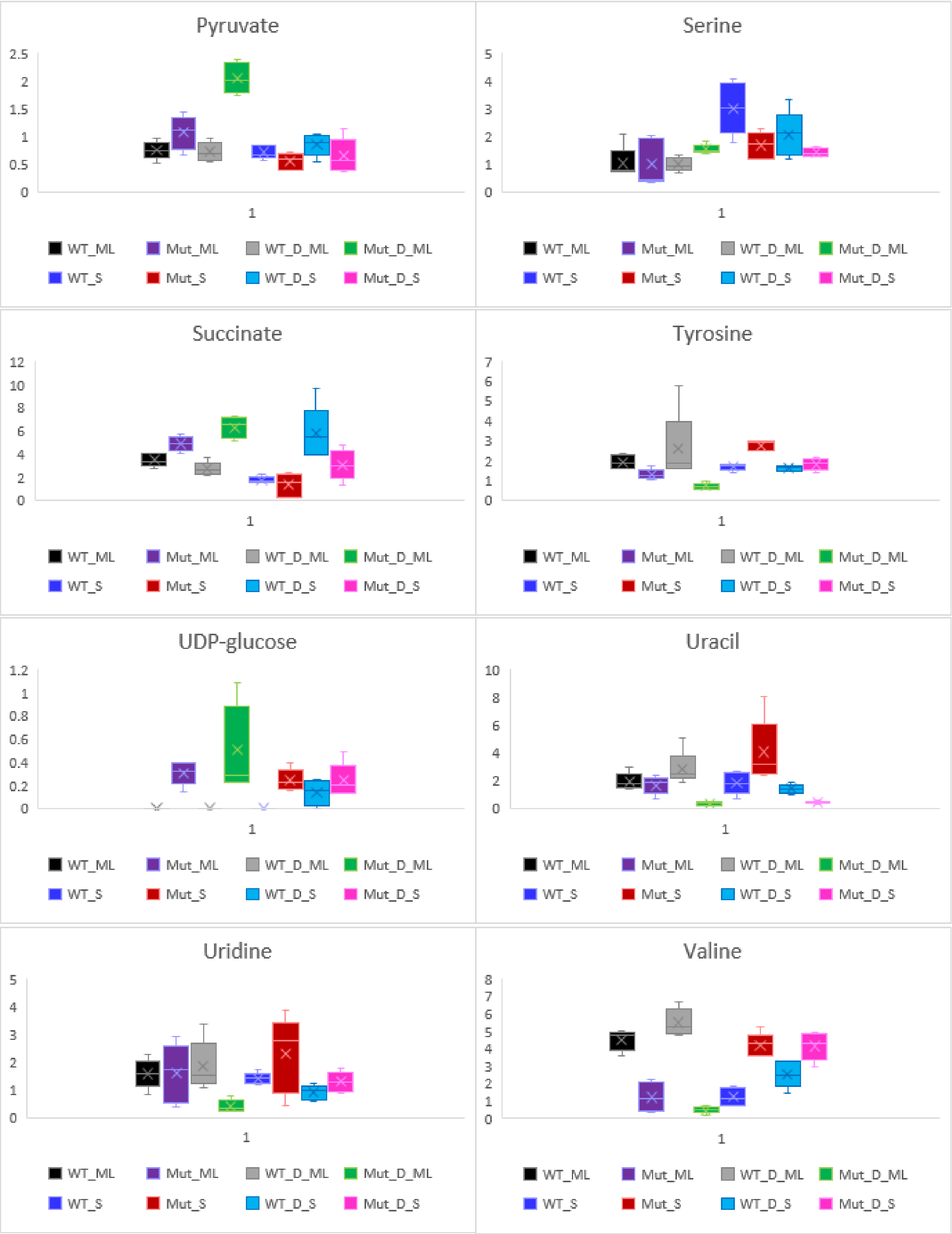

Figure S41. All Sample Types Metabolite Box and Whisker Plots. The ends of the whiskers are the minimum and maximum values, the center line is the median, the x is the mean, and the colored middle "box" encompasses the middle 50% of scores for the group.

References

Ammons, MC. B., Tripet, B. P., Carlson, R. P., Kirker, K. R., Gross, M. A., Stanisich, J. J., Copié, V. Quantitative NMR Metabolite Profiling of Methicillin-Resistant and Methicillin-Susceptible Staphylococcus aureus Discriminates between Biofilm and Planktonic Phenotypes. *J. Proteome Res.* **2014**, *13*, 2973–2985.

Aries, M.L., Cloninger, M.J. NMR Metabolomic Analysis of Bacterial Resistance Pathways Using Multivalent Quaternary Ammonium Functionalized Macromolecules**.** *Metabolomics* **2020**, *16*, 1–11. doi.org/10.1007/s11306-020-01702-1

Wang, N. S. [Online] Experiment No. 9 ºC Measurements of Cell Biomass Concentration. Department of Chemical and Biomolecular Engineering, University of Maryland, MD. http://www.eng.umd.edu/~nsw/ench485/lab9c.htm. (accessed 24 November 2019).
